# Dengue virus NS1 secretion is regulated via importin-subunit β1 controlling expression of the chaperone GRp78 and targeted by the clinical drug ivermectin

**DOI:** 10.1128/mbio.01441-23

**Published:** 2023-09-13

**Authors:** Solène Denolly, Hongbo Guo, Miriam Martens, Anna Płaszczyca, Pietro Scaturro, Vibhu Prasad, Kessiri Kongmanas, Nuntaya Punyadee, Adisak Songjaeng, Dumrong Mairiang, Andreas Pichlmair, Panisadee Avirutnan, Ralf Bartenschlager

**Affiliations:** 1 Department of Infectious Diseases, Molecular Virology, Center for Integrative Infectious Disease Research, Heidelberg University, Medical Faculty Heidelberg, Heidelberg, Germany; 2 Technical University of Munich, School of Medicine, Institute of Virology, Munich, Germany; 3 Leibniz Institute of Virology, Hamburg, Germany; 4 Division of Dengue Hemorrhagic Fever Research, Department of Research and Development, Faculty of Medicine Siriraj Hospital, Mahidol University, Bangkok, Thailand; 5 Siriraj Center of Research Excellence in Dengue and Emerging Pathogens, Faculty of Medicine Siriraj Hospital, Mahidol University, Bangkok, Thailand; 6 Molecular Biology of Dengue and Flaviviruses Research Team, National Center for Genetic Engineering and Biotechnology, National Science and Technology Development Agency, Pathumthani, Thailand; 7 Department of Immunology, Faculty of Medicine Siriraj Hospital, Mahidol University, Bangkok, Thailand; University of Pennsylvania, Philadelphia, Pennsylvania, USA; The University of Texas Southwestern Medical Center, Dallas, Texas, USA

**Keywords:** flavivirus, nuclear transport, protein secretion, nonstructural protein, host targeting therapy, glycoproteins

## Abstract

Dengue virus (DENV) is a major human pathogen. An important pathogenicity factor is non-structural protein 1 (NS1) required for viral replication and secreted from infected cells. A clinical study indicated that the anti-parasitic drug ivermectin lowers NS1 blood levels without affecting viremia. Ivermectin targets nuclear transport by binding to importin-α, but how NS1 secretion in patients is suppressed by this drug is unknown. We show that ivermectin impairs folding and secretion of endoplasmic reticulum-luminal glycoproteins, including NS1. Proteomic analysis identified chaperones interacting with NS1, including GRp78 (78-kDa glucose-regulated protein, also known as HSPA5 or BIP). This chaperone increased in abundance on DENV infection via activation of the unfolded protein response (UPR). Ivermectin blocked the nuclear transport of transcription factors required for UPR, thus impairing GRp78 upregulation and NS1 secretion. Reduction of GRp78 and NS1 secretion was also observed in patients treated with ivermectin. These results link nuclear transport and its inhibition by ivermectin to folding and secretion of luminal glycoproteins, including DENV NS1.

**IMPORTANCE:**

Dengue virus (DENV) is a major human pathogen that can cause hemorrhagic fever and shock syndrome. One important factor of DENV pathogenicity is non-structural protein 1 (NS1), a glycoprotein that is secreted from infected cells. Here we study the mode of action of the widely used drug ivermectin, used to treat parasitic infections and recently shown to lower NS1 blood levels in DENV-infected patients. We found that ivermectin blocks the nuclear transport of transcription factors required for the expression of chaperones that support the folding and secretion of glycoproteins, including NS1. Impairing nuclear transport of these transcription factors by ivermectin or depleting them from infected cells dampens NS1 folding and thus its secretion. These results reveal a novel mode of action of ivermectin that might apply to other flaviviruses as well.

## INTRODUCTION

Dengue virus (DENV) is the causative agent of dengue fever, which is the most prevalent mosquito-borne viral disease worldwide with an estimated ~100 million symptomatic infections each year and ~20,000 deaths (1). Based on climatic and socioeconomic projections, it is estimated that 60% of the world’s population will be at risk of dengue in 2080 (2).

DENV belongs to the Flavivirus genus in the *Flaviviridae* family. It is an enveloped virus with a single-stranded positive-sense RNA genome that encodes three structural proteins [capsid (C), envelope (E), and premembrane (prM)] and seven non-structural proteins (NS1, NS2A, NS2B, NS3, NS4A, NS4B, and NS5) (3). Among these proteins, NS1 plays multiple roles in the viral life cycle. On the one hand, it is involved in RNA replication and assembly/secretion of viral particles (4 – 6); on the other hand, it is secreted from infected cells and plays a critical role in immune evasion by counteracting the complement system (7, 8). Furthermore, NS1 contributes to dengue pathogenesis by triggering the release of cytokines contributing to vascular leakage (9). In infected cells, NS1 is synthesized as endoplasmic reticulum (ER) luminal glycoprotein that forms stable dimers (10). Specifically, NS1 contains two conserved *N*-glycosylation sites (10 – 12). The monomer consists of three structural domains: the β-roll dimerization domain, a wing domain, and a β-ladder domain (13). In addition, the monomer is stabilized by six intramolecular disulfide bonds. The NS1 dimer is formed by homotypic interaction between two β-roll domains, with three dimers undergoing trimerization resulting in ring-like NS1 hexamers that are secreted from infected cells (12, 14, 15).

**Fig 1:**
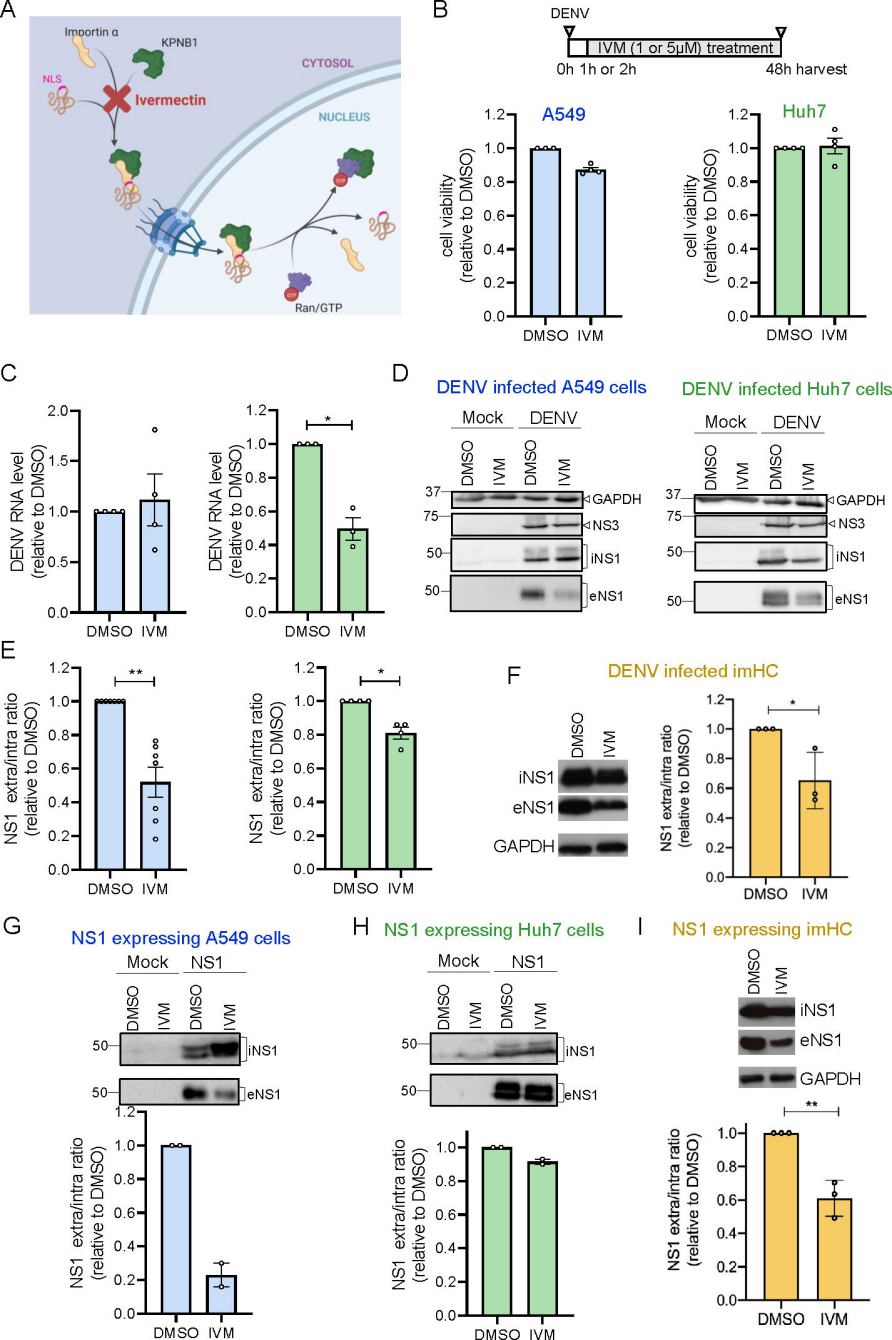
Ivermectin impairs DENV NS1 secretion. (**A**) Importin-α/KPNB1-mediated nuclear transport and inhibition by ivermectin (IVM). Created with BioRender.com. (**B**) Timing of the experimental approach is shown at the top. A549 or Huh7 cells were infected with DENV (MOI = 2) and 1 h later, cells were washed and treated with IVM (1 µM) or dimethyl sulfoxide (DMSO) for 48 h. Stem cell-derived immortalized hepatocyte-like cells (imHC) were infected with DENV at an MOI = 0.1, and 2 h later, cells were washed and treated with IVM (5 µM) or DMSO for 48 h. Bottom panels: viability of A549 (blue) and Huh7 cells (green) on IVM treatment by using CellTiter-Glo assay. Values were normalized to those of DMSO-treated cells. *N* = 4 biological replicates. (**C**) Amount of intracellular DENV RNA after infection of A549 (blue) or Huh7 cells (green) as determined by RT-qPCR. Values were normalized to those of DMSO-treated cells. *N* = 4 and *N* = 3 biological replicates for A549 and Huh7 cells, respectively. (**D**) Lysates and culture supernatants from mock and DENV-infected A549 or Huh7 cells, treated as in panel **B**, were analyzed by western blot using antibodies with specificities given on the right. GAPDH served as a loading control. iNS1, intracellular NS1; eNS1, extracellular NS1. (**E**) Quantification of NS1 signal in lysate and culture supernatant of A549 (blue) or Huh7 cells (green), displayed as the ratio of NS1 in supernatant/lysate and normalized to DMSO-treated cells. *N* = 7 biological replicates for A549 and *N* = 4 biological replicates for Huh7 cells. (**F**) Lysates and culture supernatants from DENV-infected imHC, treated as in panel **B**, were analyzed by western blot (left). Quantification of NS1 signal in lysate and culture supernatant of imHC displayed as the ratio of NS1 in supernatant/lysate and normalized to DMSO-treated cells (right). *N* = 3 biological replicates. (**G**) Lysates and supernatants of naive cells (mock) or NS1-expressing A549 cells treated for 48 h with DMSO or IVM were analyzed by western blot using NS1-specific antiserum (top). NS1 signal intensities detected in supernatant and lysate were quantified, and the ratio was normalized to values of DMSO-treated cells (bottom). *N* = 2 biological replicates. iNS1 and eNS1, intra- and extracellular NS1, respectively. (**H**) Same as panel **G** but using Huh7 cells. *N* = 2 biological replicates. (**I**) Same as panel **G** but using imHC. *N* = 3 biological replicates. Data are represented as mean ± SEM. For all graphs, each dot corresponds to the value of an individual experiment. Statistical significance was determined by one-sample *t*-test. *P < 0.05, **P < 0.01, ***P < 0.001

Currently, there are no approved antiviral drugs for the prevention or treatment of dengue; however, several strategies targeting viral proteins are pursued (16). In addition, host-targeting approaches (HTAs) are undertaken aiming for a pan-flavivirus treatment (17). In that respect, a recent clinical trial showed that treatment of DENV-infected patients with ivermectin, an antiparasitic and U.S. Food and Drug Administration-approved drug, led to accelerated clearance of NS1 from the blood without changing viral loads (18). Ivermectin is known to impair nuclear transport by binding to importin-α (19) (Fig. 1A). Cargo containing a nuclear localization signal (NLS) can bind to importin-α associated with the importin-β family member karyopherin-β1 (KPNB1, also called importin-subunit β1). The latter can mediate the transport of the cargo/importin-α/KPNB1 complexes into the nucleus via the nuclear pore complex. Within the nucleus, the complexes dissociate when KPNB1 binds to RanGTP that is present at high concentrations in this compartment (20).

Since ivermectin dampens the secretion of DENV NS1, which is a key pathogenicity factor, we sought to determine how nuclear transport could regulate NS1 secretion. We report that ivermectin-impaired nuclear transport is required for the expression of the chaperone 78-kDa glucose-regulated protein (GRP-78; also called binding immunoglobulin protein [BiP] or heat shock 70-kDa protein 5 [HSPA5]), which in turn is responsible for the folding and secretion of NS1 *in vitro* and *in vivo*.

## RESULTS

### Ivermectin treatment prevents NS1 secretion from DENV-infected cells

Since ivermectin (IVM) treatment of DENV-infected patients reduces NS1 levels in the blood with no significant effect on viremia (18), we first sought to determine if the same holds true *in vitro*. Hence, we infected A549 and Huh7 cells (to exclude cell linespecific effects) with DENV and after 1 hour, removed the inoculum and added IVM at a non-cytotoxic dose (1 µM) (Fig. 1B). At 48 h post-infection, cells and culture supernatants were harvested and intracellular viral RNA levels were quantified by quantitative reverse transcription PCR (RT-qPCR). As shown in Fig. 1C, amounts of viral RNA were not affected in A549 cells and reduced only around 2-fold in Huh7 cells arguing for only minor effect of IVM on DENV replication. A consistent subtle effect on levels of NS3 was detected by western blot (Fig. 1D). Interestingly, while there was no effect on DENV replication in A549 cells, we observed an around 2-fold decrease of NS1 secretion, as deduced from the ratio of extra- vs intracellular NS1 amounts in these cells (Fig. 1D and E, left panels), suggesting that IVM reduced NS1 secretion also *in vitro*. In Huh7 cells, we could also detect a slight reduction of NS1 secretion, in addition to impaired virus replication (Fig. 1D and E, right panels). This reduction of NS1 secretion became even more obvious when quantifying NS1 amounts by enzyme linked immunosorbent assay (ELISA)-based assay (Fig. S1A through C). Of note, we could confirm the effect of IVM treatment on NS1 secretion using a 2-h treatment with a higher dose (25 µM), corroborating an NS1 secretion inhibition independent from effects of the drug on DENV replication Fig. S1D through H. In addition, we confirmed the results in stem cell-derived immortalized hepatocyte-like cells (imHC), which is a robust non-cancerous cell model (21). In this case, imHC were infected with DENV and after 2 h, we removed the inoculum and added IVM at a final concentration of 5 µM. NS1 secretion was also impaired in IVM treated and DENV-infected imHC (Fig. 1F), confirming that IVM impairs NS1 secretion in different cell systems.

To confirm that IVM impairs NS1 secretion independent of viral RNA replication, we used A549 and Huh7 cells as well as imHC, all stably expressing NS1 in the absence of other viral proteins. Cells were treated with IVM for 48 h, and amounts of intra- vs extracellular NS1 were determined by quantitative western blot. While no effect of IVM was found in Huh7 cells, secretion of NS1 was significantly impaired in A549 cells and imHC (Fig. 1G through I). These results suggest that observations made in patients are best reflected in imHC and A549 cells. The latter were used for further analyses because of their ease to handle and propagate.

### Karyopherin β1 (KPNB1) is involved in NS1 secretion

**Fig 2:**
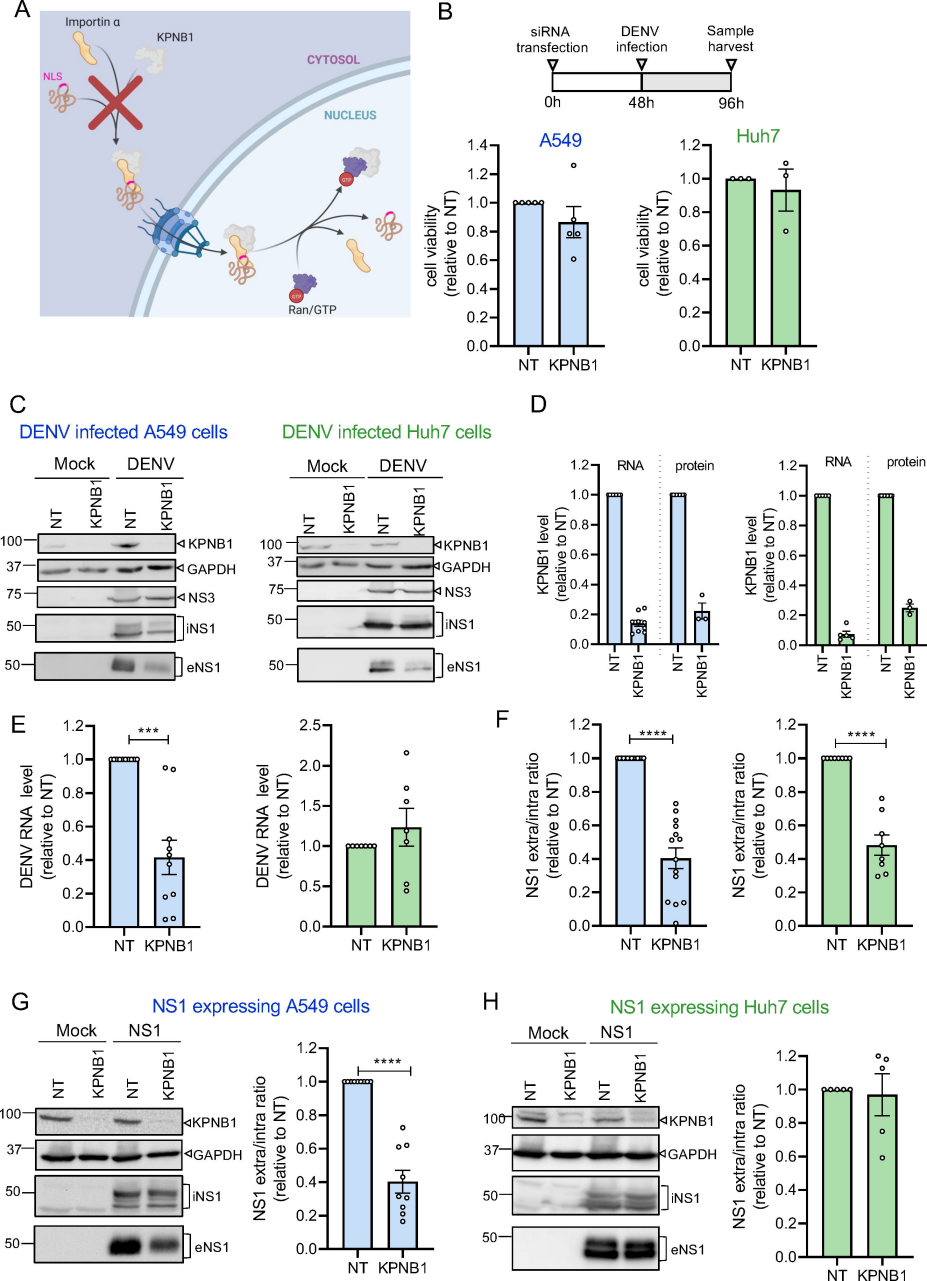
KPNB1 knock-down impairs DENV NS1 secretion. (**A**) Importin-α/KPNB1-mediated nuclear transport and inhibition by KPNB1 knock-down. Created with BioRender.com. (**B**) Timing of the used protocol is shown at the top. Cells were transfected with KPNB1-specific or non-targeting (NT) siRNAs. After 48 h, cells were infected with DENV at an MOI = 2. Samples were harvested 48 h post-infection. Bottom panels: Cell viability of A549 (blue) and Huh7 cells (green) on KPNB1 KD as determined by CellTiter-Glo assay. Values were normalized to those of cells transfected with NT siRNAs. *N* = 3 biological replicates. (**C**) Lysates and supernatants from mock vs DENV-infected A549 or Huh7 cells treated as in (**B**) were analyzed by western blot using antibodies with specificities indicated on the right of each panel. GAPDH served as a loading control. iNS1 and eNS1, intra- and extracellular NS1, respectively. (**D**) Determination of KD efficiency at the RNA level by using RT-qPCR or at the protein level by using western blot. Values were normalized to those obtained with cells transfected with non-targeting siRNAs that were set to 1. Number of biological replicates: *N* = 9 for A549-RNA; *N* = 3 for A549-protein; *N* = 5 for Huh7-RNA; and *N* = 3 for Huh7-protein. (**E**) Amount of intracellular DENV RNA after infection of A549 (blue) or Huh7 cells (green), determined by RT-qPCR and normalized to siNT-transfected cells. *N* = 10 biological replicates for A549 and *N* = 7 biological replicates for Huh7 cells. (**F**) Quantification of NS1 signals in lysates and supernatants of A549 (blue) or Huh7 cells (green). Values are displayed as the supernatant/lysate ratio after normalization to siNT-transfected cells. *N* = 14 biological replicates for A549 and *N* = 8 biological replicates for Huh7 cells. (**G**) Lysates and supernatants from mock and NS1-expressing A549 cells, transfected with KPNB1 or NT siRNA and harvested as in panel **B** were analyzed by western blot (left). Ratio of supernatant/lysate was determined and values were normalized to those of siNT-transfected cells (right). *N* = 9 biological replicates. (**H**) Same as in panel **G** but using Huh7 cells. *N* = 5 biological replicates. Data were represented as mean ± SEM. For all graphs, each dot corresponds to the value of an individual experiment. Statistical significance was determined by one-sample *t*-test. *P < 0.05, **P < 0.01, ***P < 0.001, ****P < 0.0001.

As IVM is known to block importin-α/importin-β interaction, thus preventing nuclear transport (Fig. 1A), we investigated if nuclear transport might regulate NS1 secretion. To this end, we downregulated KPNB1, a member of the importin-β family, using RNA interference (Fig. 2A). Forty-eight hours after small interfering RNA (siRNA) transfection, cells were infected with DENV and 48 h later, cells and culture supernatants were collected and cell viability (Fig. 2B) as well as knock-down (KD) efficiency were determined (Fig. 2C and D). KPNB1 KD reduced DENV viral RNA in A549 cells but had no impact in Huh7 cells (Fig. 2E). Although this cell line-specific effect was opposing to the one observed with IVM treatment (Fig. 1C), we consistently found that KPNB1 KD impaired NS1 secretion in both cell lines (Fig. 2F; Fig. S1I and J), reflecting IVM treatment results (Fig. 1E).

Next, we tested the effect of KPNB1 KD on NS1 secretion independent of viral replication by using cells stably expressing NS1. We found that NS1 secretion was impaired in NS1-expressing A549 cells, while no effect was detected with Huh7 cells (Fig. 2G and H). Taken together, these results indicate that nuclear transport is involved in NS1 secretion in DENV-infected A549 cells and that the phenotype is cell line dependent.

### A more general role of KPNB1 and nuclear transport in protein secretion

**Fig 3:**
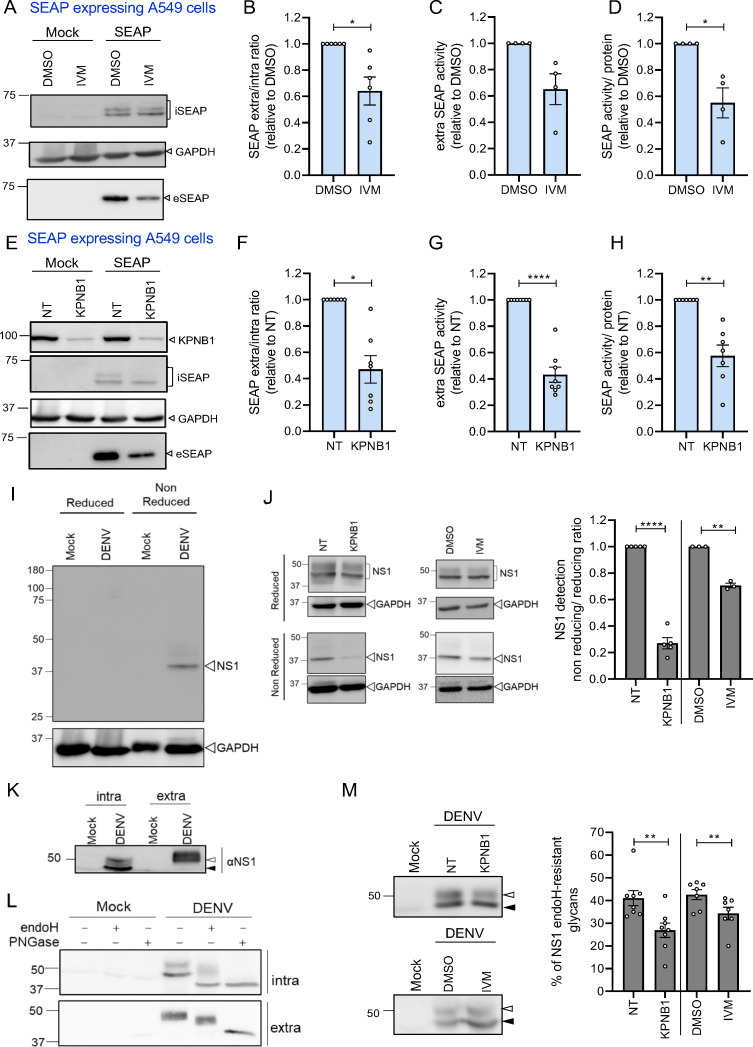
Nuclear transport blockage prevents the secretion and activity of secreted alkaline phosphatase (SEAP). (**A**) A549 cells expressing SEAP or control (mock) cells were treated for 48 h with DMSO or IVM (1 µM). Lysates and supernatants were analyzed by western blot using a SEAP-specific antibody. iSEAP and eSEAP, intra- and extracellular SEAP, respectively. (**B**) Quantification of the SEAP signal in lysates and culture supernatant of cells treated like in panel **A**. Values are displayed as the ratio of supernatant/lysate and normalized to those of DMSO-treated cells. *N* = 5 biological replicates. (**C**) Extracellular phosphatase activity released from cells treated like in panel **A**. Values were normalized to those of DMSO-treated cells. *N* = 4 biological replicates. (**D**) Intracellular phosphatase activity normalized to SEAP abundance determined by western blot of cell lysates obtained like in panel **A**. The ratio was normalized to values obtained for DMSO-treated cells. *N* = 4 biological replicates. (**E**) A549 cells expressing SEAP were transfected with KPNB1-targeting or NT siRNAs. Forty-eight hours post-transfection, the medium was changed for 48 h, and cell lysate and culture supernatant were harvested 48 h later. Samples were analyzed by western blot using a SEAP-specific antibody. (**F**) Quantification of SEAP signals in western blots as shown in panel **E**. *N* = 7 biological replicates. (**G**) Quantification of SEAP activities as in panel **C**. *N* = 8 biological replicates. (**H**) SEAP activity normalized to total intracellular SEAP amounts. Values in panels **F – G** were normalized to those obtained with siNT-transfected cells. *N* = 7 biological replicates. ( **I**) Lysates of mock vs DENV-infected A549 cells were analyzed by western blot after sample loading under reducing or non-reducing conditions and detection of NS1 using conformation-specific DN3 antibody. (**J**) Lysates of A549 cells transfected with given siRNAs for 48 h and infected with DENV for 48 h (left panel) or infected with DENV and treated with IVM (1 µM) or DMSO for 48 h (right panel) were analyzed by NS1-specific western blot after sample loading under reducing or non-reducing conditions. For quantification, NS1 signal was normalized to GAPDH and the ratio of non-reducing over reducing sample signal was calculated. Values were normalized to those obtained with siNT-transfected or IVM-treated cells. Data are represented as mean ± SEM of three or six independent experiments. (**K**) NS1 in lysate (intra) and culture supernatant (extra) of mock and DENV-infected A549 cells. (**L**) Lysate (intra) and supernatant (extra) of DENV-infected A549 cells were treated with endoH or PNGase prior to NS1-specific western blot analysis. (**M**) Lysates of A549 cells transfected with given siRNAs for 48 h and infected with DENV for 48 h (top panel) or infected with DENV and treated with IVM (1 µM) or DMSO for 48 h (bottom panel) were analyzed by NS1-specific western blot. Empty and filled arrowheads point to mature and immature forms of NS1, respectively. Quantification of seven or eight independent biological replicates is shown on the right. Data are represented as mean ± SEM. Each dot in the graphs corresponds to the value of an individual experiment. In all graphs, statistical significance was determined by one-sample *t*-test or ratio-paired *t*-test for panel M. *P < 0.05, **P < 0.01, ***P < 0.001, ****P < 0.0001.

We next wondered if the effect of IVM treatment or KPNB1 KD was specific to the secretion of DENV NS1 or a more general phenomenon applying to the secretion of proteins via the constitutive secretory pathway. To this end, we expressed a reporter glycoprotein, i.e., the secreted alkaline phosphatase (SEAP), which is known to be glycosylated and secreted from the ER via the constitutive secretory pathway (22). We focused this analysis on A549 cells since we observed no effect on NS1 secretion by either IVM treatment or KPNB1 KD in Huh7 cells in the absence of DENV infection (Fig. 1H and 2H). Interestingly, we observed decreased SEAP secretion on IVM treatment (Fig. 3A and B) or KPNB1 KD (Fig. 3E and F), suggesting that the role of nuclear transport for protein secretion is not restricted to DENV NS1.

To complement this analysis, we measured the extra- and intracellular enzymatic activity of SEAP, the latter to determine if nuclear transport inhibition might affect SEAP-specific activity in addition to impairing its secretion. We observed a decrease of SEAP activity in the culture supernatant of SEAP-expressing cells after IVM treatment (Fig. 3C) and after KPNB1 KD (Fig. 3G), correlating with the decrease of SEAP secretion determined by western blot (Fig. 3A and E). Most interestingly, we also observed reduced specific SEAP activity, that is, enzymatic activity per protein amount (Fig. 3D and H). Since SEAP activity is highly dependent on its folding state, we hypothesized that reduced nuclear transport impaired secretion as a consequence of improper SEAP folding.

### Proper glycosylation and folding of NS1 depend on nuclear transport

Building on this hypothesis and extrapolating to NS1, we monitored NS1 folding by using as an approximation NS1 detection by western blot with the monoclonal antibody DN3. This antibody recognized NS1 only under non-reducing condition (Fig. 3I) suggesting that DN3 recognizes an epitope present in NS1 only in a native conformation. Using this approach, we observed decreased recognition of NS1 expressed in KPNB1 KD or IVM-treated cells (Fig. 3J), arguing for altered NS1 conformation on KPNB1 depletion or pharmacological inhibition. No such reduction was found when we used reducing conditions and detection with a polyclonal antiserum raised against denatured protein (Fig. 3J).

As impaired folding might result in increased retention of NS1 in the ER, we also determined the dependency of NS1 *N*-glycans’ maturation on the nuclear transport integrity of the cell. NS1 has two *N*-glycosylation sites. Initially, NS1 is fully sensitive to treatment with endoglycosidase H (endoH), consistent with NS1 synthesis in the ER, but during Golgi transport acquires partial endoH resistance because of trimming of one of the two added glycans (12). Therefore, in unperturbed DENV-infected cells, intra- and extracellular NS1 have different electrophoretic mobilities (Fig. 3K). Interestingly, we could observe two protein species for intracellular NS1, probably reflecting different degrees of maturation. As expected, secreted NS1 was partially endoH-resistant while intracellular NS1 was mainly endoH-sensitive, although we could also detect a fraction of partially endoH-resistant intracellular NS1, most likely contributing to NS1 transported to the Golgi and having undergone maturation, but not yet being secreted (Fig. 3L). This led us to conclude that the slower migrating NS1 species (empty triangle in Fig. 3K) is the mature form, while the faster migrating protein (filled triangle in Fig. 3K) corresponded to immature NS1.

To determine the impact of nuclear transport on NS1 maturation, we quantified the proportion of mature NS1 in KPNB1 KD or IVM-treated and DENV-infected cells. Interestingly, while we detected ~40% of NS1 with endoH-resistant glycans, this proportion dropped to ~27% on KPNB1 KD and 34% on IVM treatment (Fig. 3M). Since endoH-resistance is acquired in the cis-Golgi, our result suggested that NS1 might be blocked in the ER, possibly due to a folding defect. Based on this assumption and the results obtained with SEAP, we hypothesized that nuclear transport is involved in the regulation of proper glycoprotein folding, which is necessary for glycoprotein transport through the secretory pathway.

### Proteins involved in folding are enriched in the NS1 interactome

**Fig 4:**
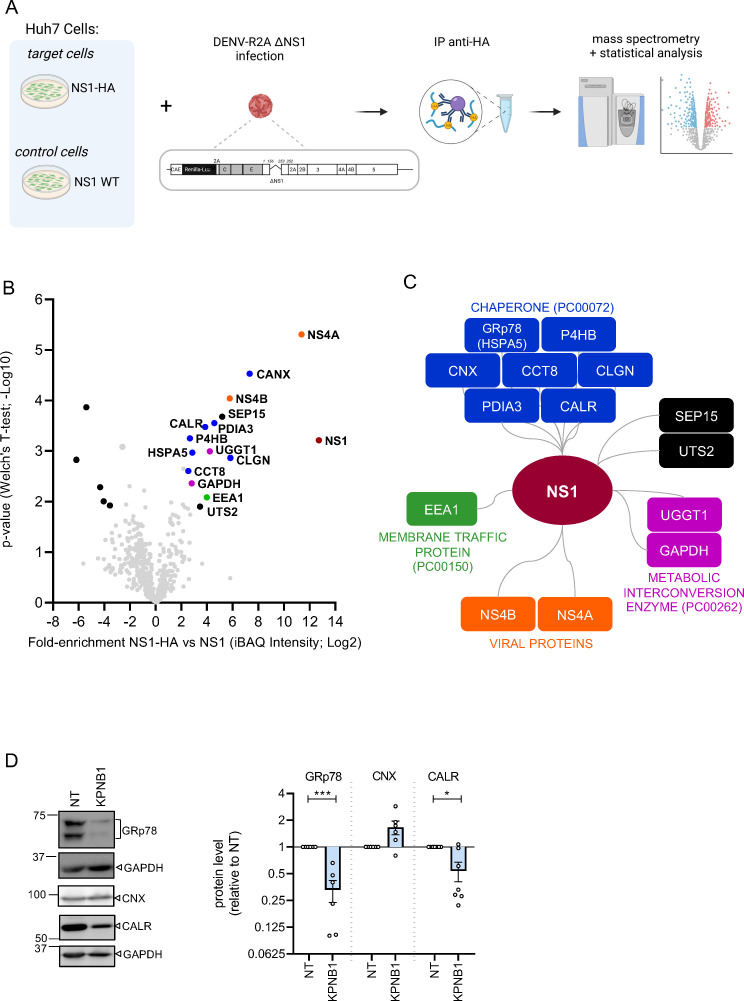
NS1 proteome reveals enrichment of host cell proteins involved in protein folding. (**A**) Schematic of the experimental approach used to enrich NS1. Huh7 cells stably expressing NS1 or NS1-HA were infected with a DENV reporter virus containing a deletion in NS1 that destroys replication competence (DENV-R2A ΔNS1; MOI = 1). This mutant is rescued by NS1 or NS1-HA expressed in the Huh7 helper cells. Cells were lysed 72 h post-infection, and NS1 contained therein was enriched by affinity purification using anti-HA-coated beads. Captured protein complexes were subjected to mass spectrometry analysis. (**B**) Cellular and viral proteins interact with NS1 on DENV infection. Volcano plot displaying the average degree of enrichment of NS1-HA over NS1 WT (ratio of intensity-based absolute quantification [iBAQ] protein intensities) and the *P* value (Welch’s *t*-test) for each protein. Viral and host proteins significantly enriched in NS1_HA immunoprecipitations are indicated in color according to their gene ontology enrichment (*N* = 4; *P*-value < 0.05, false discovery rate = 0.01). (**C**) Interaction network of NS1-associated proteins. Proteins were clustered according to “protein class” gene ontology enrichment. (**D**) Lysates of A549 cells infected with DENV and transfected with KPNB1-targeting or NT siRNAs were analyzed by western blot. Identified proteins are specified on the right of each blot. GAPDH served as a loading control. Quantification of protein signals is shown on the right panel. Values were normalized to GAPDH and are given relative to siNT-transfected cells. *N* = 7–8 biological replicates. Data are represented as mean ± SEM. Each dot in the graphs corresponds to the value of an individual experiment. Statistical significance was determined by one-sample *t*-test. *P < 0.05 and ***P < 0.001.

Next, we sought to obtain a comprehensive overview of proteins involved in NS1 folding and, therefore, determined the NS1 interactome in DENV-infected cells. To this end, we took advantage of our trans-complementation system (6) that is based on Huh7 cells in which we had observed reduced NS1 secretion on IVM treatment (Fig. 1E; Fig. S1C) and KPNB1 KD (Fig. 2F; Fig. S1G). These cells were infected with a DENV-R2A ΔNS1 mutant that was rescued by stably expressed wild-type (WT) NS1 or hemagglutinin (HA)-tagged NS1 (Fig. 4A). HA-specific beads were used to pull down NS1-HA, and the composition of captured protein complexes was determined by mass spectrometry (for a complete list of NS1 interactants, see Table S1). Bioinformatics analysis revealed a number of significantly enriched proteins. These included the viral proteins NS4A and NS4B earlier shown to interact with NS1 via the NS4A-2k-4B precursor (5), thus validating the accuracy of our trans-complementation assay (Fig. 4B). In addition, we identified 12 high-confidence interactors of NS1, and seven of these having chaperone activity according to gene ontology (GO) classification (Fig. 4C), arguing for a strong requirement of NS1 folding for host cell chaperones. These included calnexin, calreticulin, and GRp78 (aka BiP that is encoded by *HSPA5*), earlier reported as NS1 interactors (23, 24). We also detected CCT8, a member of the CTT complex also shown to interact with NS1 (24). Interestingly, we also identified two additional proteins with chaperone activity, namely Erp57 (also called PDIA3) and P4HB (also called PDIA1), two protein disulfide isomerases.

### KPNB1 and nuclear transport affect NS1 secretion by regulating GRp78 level

Given the strong interaction of NS1 with multiple chaperones, we next determined the effect of KPNB1 KD on the expression levels of some of these chaperones, that is*,* calnexin, calreticulin, and GRp78. Interestingly, we found that KPNB1 KD led to a strong decrease in total GRp78 and, to a lesser extent, calreticulin levels without impacting calnexin abundance (Fig. 4D).

**Fig 5:**
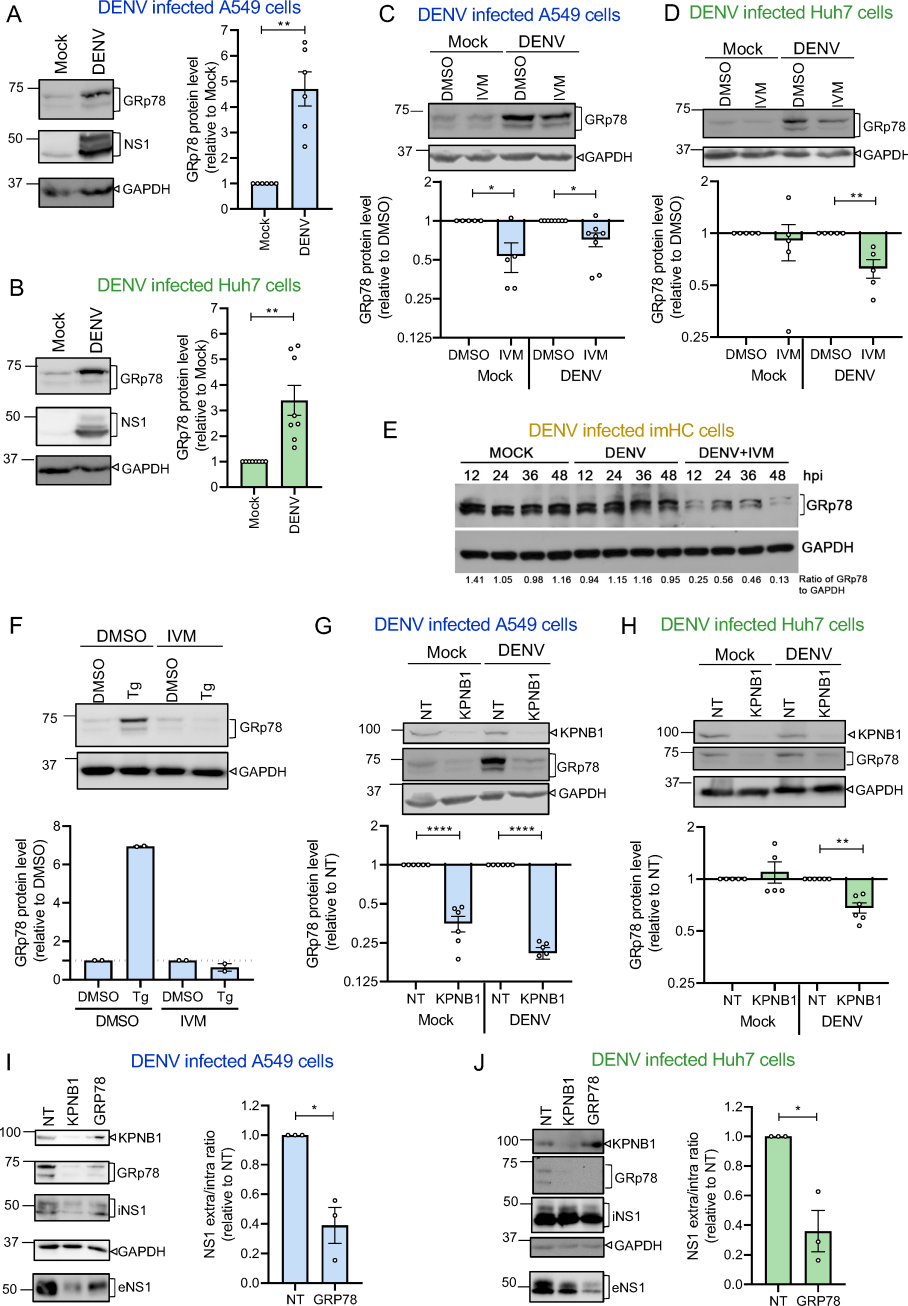
Nuclear transport via KPNB1 is required for the upregulation of GRp78 regulating NS1 secretion. (**A**) Representative western blot (left) and quantification of GRp78 level (right) in A549 cell lysates after mock or DENV infection. Values were normalized to those obtained with mock cells that were set to 1. *N* = 6 biological replicates. (**B**) Same as panel **A** but using Huh7 cells. *N* = 8 biological replicates. (**C**) Representative western blot (top) of lysates of A549 cells treated with IVM (1 µM) as described in Fig. 1B, and quantification of GRp78 signals detected by western blot (bottom). Values are given relative to DMSO-treated cells. *N* = 6–8 biological replicates. (**D**) Same as panel **C** but for Huh7 cells. *N* = 5 biological replicates. (**E**) Representative western blot of lysates of imHC cells treated with IVM (5 µM) 2 h post-infection (MOI = 0.1) and harvested at different time points post-infection. Quantification of GRp78 signals normalized to GAPDH is given below each lane. (**F**) Representative western blot (top) of lysates of A549 cells treated with DMSO or thapsigargin (Tg, 10 µM), or DMSO or IVM (25 µM) for 6 h prior to lysis, and quantification of GRp78 signals detected by western blot (bottom). Values were normalized to those of DMSO-treated cells. *N* = 2 independent biological replicates. (**G**) Representative western blot (top) of lysates of A549 cells transfected with KPNB1-targeting siRNAs using the timing given in Fig. 2B top panel, and quantification of GRp78 signals detected by western blot (bottom). Values are given relative to siNT-transfected cells. *N* = 6 biological replicates. (**H**) Same as panel **G** but for Huh7 cells. *N* = 5–6 biological replicates. (**I**) A549 cells were transfected with GRp78- or non-targeting (NT) siRNAs prior to DENV infection (MOI = 2). After 48 h, cells and culture supernatants were harvested and analyzed by western blot. A representative western blot for NS1 is shown on the left; quantification of NS1 signals is given on the right. Values are displayed as the ratio of supernatant/lysate, normalized to siNT-transfected cells. *N* = 3 biological replicates. (**J**) Same as panel **I** but using Huh7 cells. *N* = 3 biological replicates. In each graph, data are represented as mean ± SEM. Each dot in the graphs corresponds to the value of an individual experiment. Statistical significance was determined by one-sample *t*-test. *P < 0.05, **P < 0.01, ***P < 0.001, ****P < 0.0001.

Since the level of GRp78 was reduced most strongly by KPNB1 KD and since this chaperone was reported to be upregulated on DENV infection (25), we focused our subsequent analysis on this chaperone. First, we confirmed that DENV infection increased GRp78 levels using both A549 and Huh7 cells (Fig. 5A and B). Importantly, we found that IVM treatment reduced GRP78 abundance in both mock and DENV-infected A549 cells as well as infected Huh7 cells (Fig. 5C and D). Although DENV infection did not induce upregulation of GRp78 in imHC, the abundance of this chaperone was profoundly reduced on IVM treatment (Fig. 5E). Inhibition of Grp78 expression by IVM could also be observed when ER stress was induced by thapsigargin, an inhibitor of the sarcoplasmic/endoplasmic reticulum Ca^2+^ ATPase (Fig. 5F), arguing for a more general role of nuclear transport in ER stress response.

Next, we determined the impact of KPNB1 KD on GRP78 abundance in A549 and Huh7 cells and observed the reduction in both mock and DENV-infected A549 cells as well as DENV-infected Huh7 cells (Fig. 5G and H). As KPNB1 KD or IVM treatment also had no effect on NS1 secretion in NS1-expressing Huh7 cells (Fig. 1H and 2I), we assumed that the expression level of GRp78 in Huh7 cells might be critical for NS1 secretion and that nuclear transport regulates NS1 secretion by regulating GRp78 levels. To corroborate this assumption, we conducted KD experiments in A549 and Huh7 cells using siRNAs targeting GRp78. Obtained results revealed that GRp78 downregulation impaired NS1 secretion from DENV-infected cells to an extent comparable to the one induced by KPNB1 KD (Fig. 5I and J; Fig. S2A and B) We also found that GRp78 KD impaired the secretion of NS1 expressed on its own (Fig. S2C and D) and reduced SEAP secretion as well (Fig. S2E through H). Moreover, we detected a strong colocalization of GRp78 with NS1 in DENV-infected A549 cells (Fig. S2I and J). Interestingly, KD of calreticulin, whose level was also decreased on KPNB1 KD (Fig. 4D), did not impair NS1 secretion (Fig. S2K), suggesting that GRp78 is specifically involved in NS1 secretion. Altogether, these results indicated that nuclear transport affects NS1 secretion through the regulation of GRp78 level within infected cells. Analogous findings made with SEAP suggested that this pathway might be of more general relevance for secreted glycoproteins.

### Regulation of GRp78 expression level by nuclear transport of spliced XBP1

**Fig 6:**
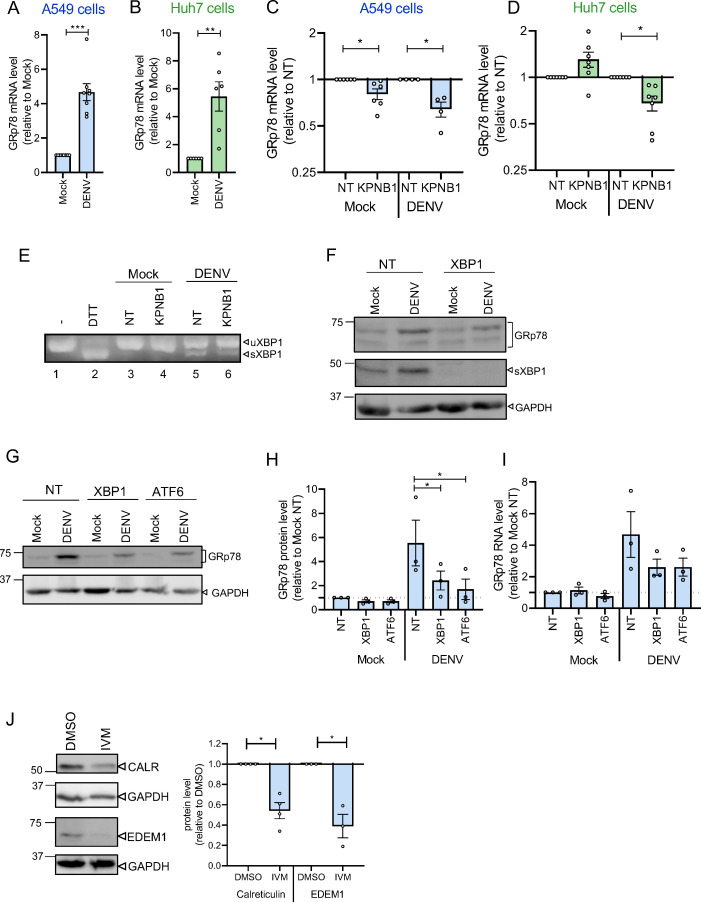
GRp78 level is regulated via the UPR requiring nuclear translocation of XBP1 and ATF6. (**A**) mRNA levels of GRp78 in mock vs DENV-infected A549 cells. Values were normalized to those of mock-infected cells. *N* = 8 biological replicates. (**B**) Same as panel **A**, but using Huh7 cells. *N* = 6 biological replicates. (**C**) mRNA levels of GRp78 in mock vs DENV-infected A549 cells transfected with KPNB1-specific siRNAs. *N* = 4–6 biological replicates. (**D**) Same as panel **C**, but using Huh7 cells. *N* = 7 biological replicates. (**E**) Splicing of XBP1 RNA as determined by RT-PCR using total RNA of A549 cells transfected with KPNB1- or non-targeting (NT) siRNAs. Treatment of cells with Dithiothreitol (DTT) for 30 min was used as a positive control. Arrowheads point to unspliced (u) and spliced (s) XBP1 mRNA. (**F**) A549 cells were transfected with XBP1 or non-targeting (NT) siRNA and 48 h later, infected with DENV (MOI = 2). After 48 h, cells were harvested and lysates were analyzed by western blot for GRp78 and XBP1 abundance. GAPDH served as a loading control. (**G**) Same as panel **F** with cells transfected with XBP1, or ATF6-targeting or non-targeting (NT) siRNAs. (**H**) Quantification of GRp78 protein levels in cells treated as in panel **G**, determined by western blot. Values were normalized to GAPDH and mock-NT signals. *N* = 3 biological replicates. (**I**) mRNA level of GRp78 in cells treated as in panel **G**. Data were normalized to mock-NT signals. *N* = 3 biological replicates. (**J**) Lysates of A549 cells infected with DENV (MOI = 2) and treated with DMSO or IVM were analyzed by western blot. Identified proteins are specified on the right of each blot. GAPDH served as a loading control. Quantification of protein signals is shown on the right panel. Values were normalized to GAPDH and are given relative to DMSO-treated cells. *N* = 3 independent biological replicates. In all graphs, data are represented as mean ± SEM. Each dot in the graphs corresponds to the value of an individual experiment. Statistical significance was determined by one-sample *t*-test or by an ordinary one-way ANOVA for panels H and I. *P < 0.05, **P < 0.01, ***P < 0.001.

Abundance of GRp78 might be regulated at the transcriptional level or by altered protein synthesis or degradation rate. We considered transcriptional regulation as the more likely possibility because nuclear transport is required for the import/export of transcription factors involved in GRp78 mRNA synthesis. We therefore quantified the GRp78 mRNA level and confirmed that DENV induced GRp78 expression at the transcriptional level around 5-fold in A549 and Huh7 cells (Fig. 6A and B). Notably, we observed a decrease of GRp78 mRNA level on KPNB1 KD in DENV-infected cells, correlating well with reduced GRp78 protein amounts (Fig. 6C and D). This suggested that nuclear transport via KPNB1 is required to induce GRp78 transcription.

GRp78 is known to be induced in response to ER stress via activation of the unfolded protein response (UPR). Furthermore, DENV was shown to induce the three arms of the UPR, i.e*.,* inositol-requiring kinase 1 (IRE1 or ERN1), protein kinase R (PKR)-like ER kinase (PERK or EIF2AK3), and activating transcription factor 6 (ATF6) (26 – 28). IRE1 activation induces the cleavage of X-box-binding protein 1 (XBP1) mRNA, leading to the production of a protein called sXBP1. Since sXBP1 is a multifunctional transcription factor (29), we hypothesized that it might be involved in the upregulation of GRp78 on DENV infection. First, we confirmed that DENV infection induced XBP1 mRNA splicing (Fig. 6E), consistent with an earlier report (30), leading to upregulation of sXBP1 protein level (Fig. 6F). Of note, we could not detect any obvious difference in XBP1 mRNA splicing on KPNB1 KD (Fig. 6E, compare lanes 3 and 4 with lanes 5 and 6). Second, XBP1 KD reduced the increase of both GRp78 protein and mRNA levels on DENV infection (Fig. 6F through I), suggesting that XBP1 is involved in the regulation of GRp78 abundance, consistent with a previous report (29). Third, ATF6 KD also impaired DENV-induced GRp78 increase (Fig. 6G and H), while PERK KD did not impact GRp78 level (Fig. S3A through C), suggesting that the ATF6 arm might also be activated by DENV and required for GRp78 induction. Fourth, we confirmed that IVM treatment also impaired the level of two other targets of ATF6/XBP1 in DENV-infected cells, namely, calreticulin (31) and endoplasmic reticulum degradation enhancer, mannosidase alpha-like 1 (EDEM-1) (32) (Fig. 6J).

Next, we sought to determine if KPNB1 could regulate XBP1 protein (pXBP1) nuclear translocation. Interestingly, we detected a decreased proportion of nuclear XBP1 in DENV-infected cells after KPNB1 KD (Fig. S3D). These results provide compelling evidence that sXBP1 is involved in the regulation of GRp78 levels on DENV infection and that KPNB1 is required for pXBP1 nuclear localization.

### Ivermectin decreases GRp78 and NS1 levels in DENV-infected patients

**Fig 7:**
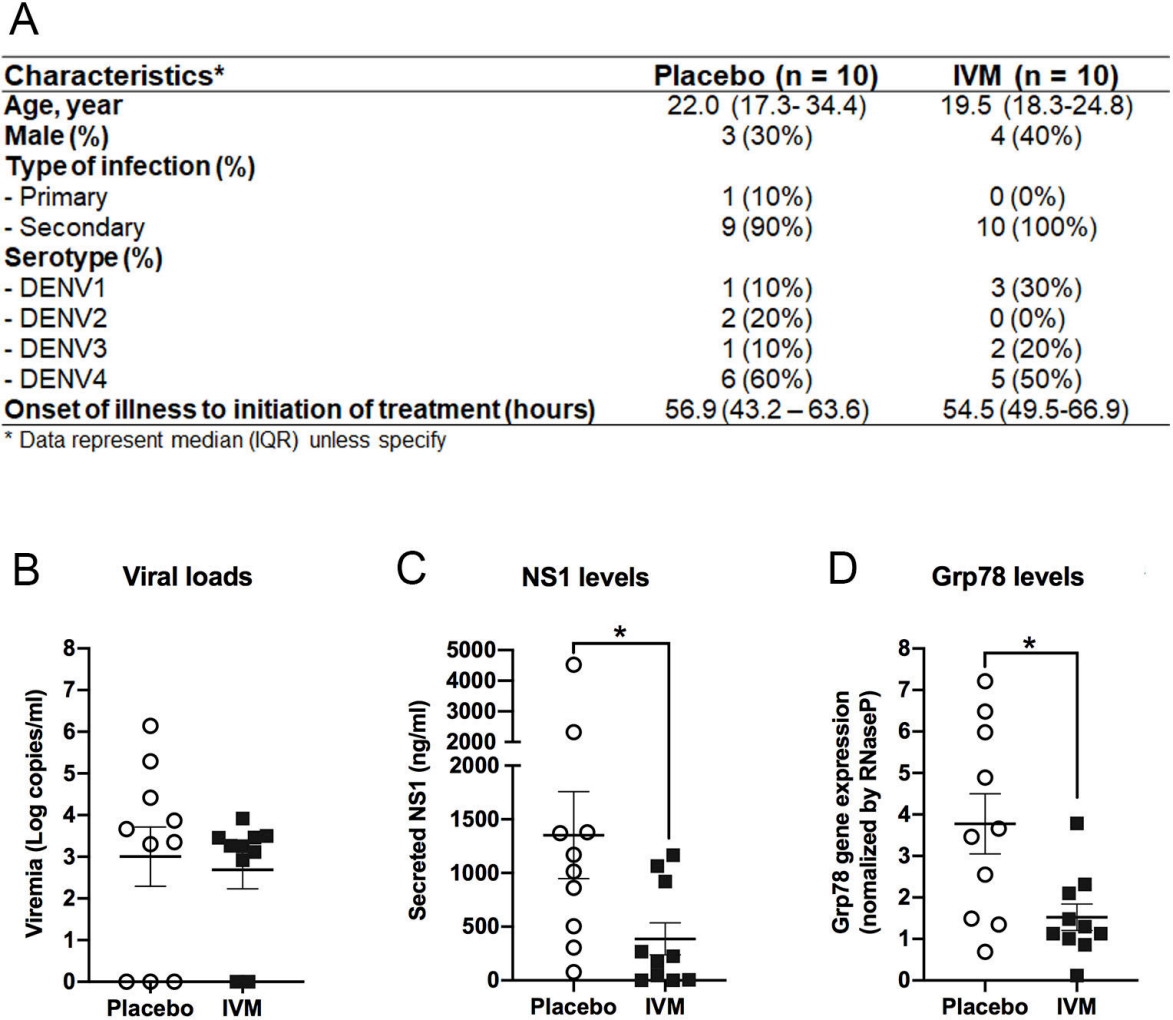
Significant reduction of NS1 and Grp78 levels in plasma samples of dengue patients following ivermectin administration. (**A**) Characteristics of dengue patients treated with placebo or IVM (10 patients per group) and used for this study. (**B**) Amounts of viral genome copies in plasma samples of the two groups of dengue patients treated or not with IVM. (**C**) Secreted NS1 in same groups as in panel **B**. (**D**) Grp78 RNA amounts in plasma samples of the two groups of dengue patients treated or not with IVM. Each dot in the scatter plot represents the result from a single patient, and the mean ± SEM of data for each group is given. Statistical significance between placebo and IVM groups was determined by unpaired *t*-test. *P = 0.0108.

**Fig 8:**
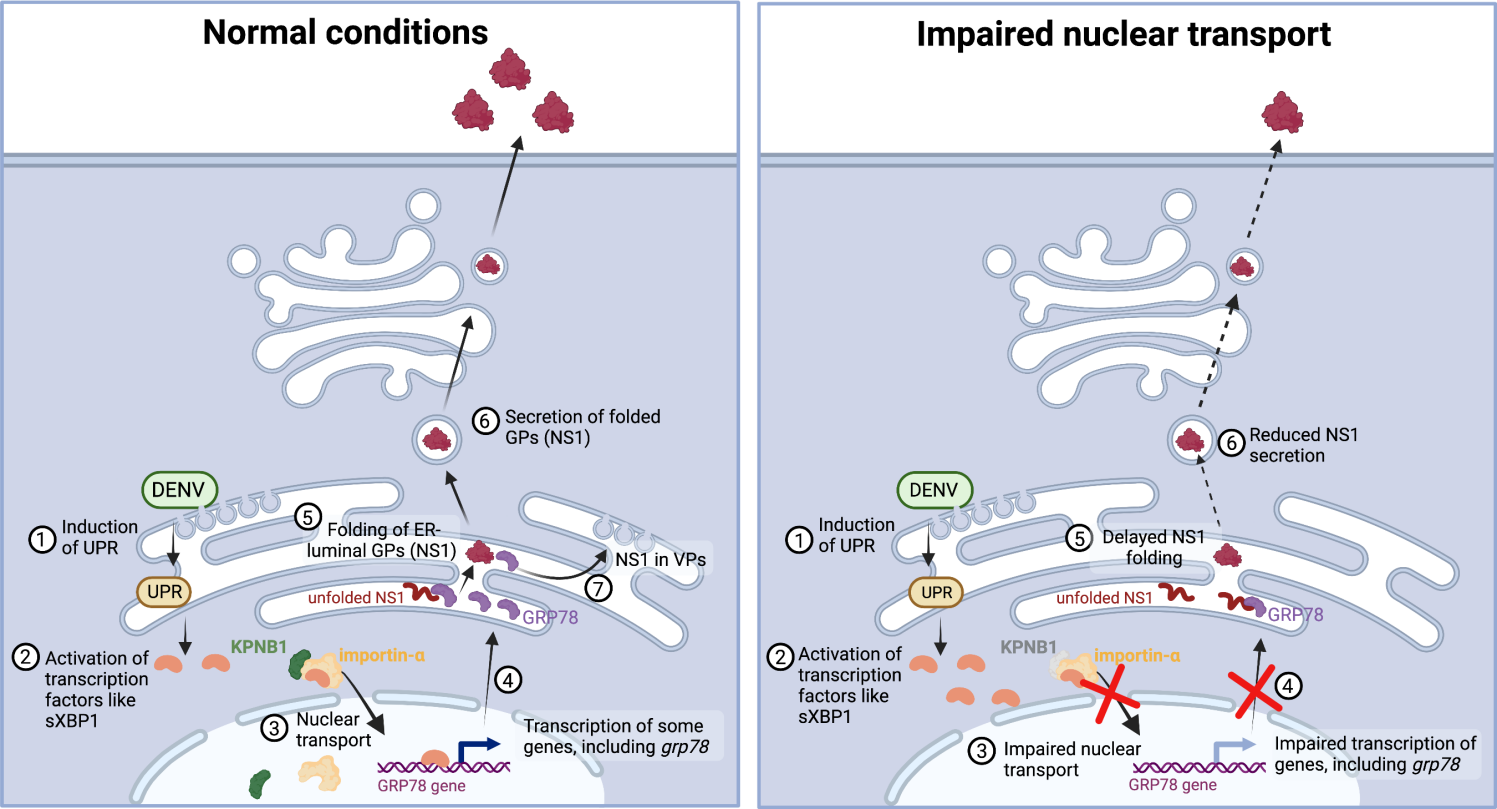
Proposed model for the role of nuclear transport in NS1 secretion. See Discussion for detailed description. Created with BioRender.com.

The data described so far point to a mechanism of how IVM affects DENV NS1 secretion *in vitro*. To validate these data *in vivo*, we used patient samples from a clinical trial in which DENV-infected patients had been treated with IVM or placebo for a duration of 3–4 days (Fig. 7A). For each group, 10 patients were selected and plasma samples were analyzed by qRT-PCR to quantify the amounts of DENV RNA reflecting viral replication and GRp78 mRNA [most likely contained in extracellular vesicles as reported earlier (33)]. In addition, NS1 protein amounts in patient plasma were quantified by ELISA. As expected, IVM treatment did not change viral load (Fig. 7B) but reduced the level of circulating NS1 (Fig. 7C). Of note, when we analyzed the GRp78 mRNA level in the plasma of patients treated with IVM vs placebo, IVM treatment significantly reduced the amount of GRp78 mRNA (Fig. 7D), consistent with our *in vitro* data. Taken together, these data show that IVM impairs NS1 secretion by reducing the abundance of host cell chaperones, notably GRp78, required for proper folding and thus the release of NS1. Mechanistically, IVM-affected nuclear transport dampens DENV-induced UPR by impairing the nuclear import of transcription factors required for the upregulation of chaperone gene expression (Fig. 8).

## DISCUSSION

Our results highlight the involvement of nuclear transport in DENV NS1 secretion and reveal the mode of action of IVM in this pathway (Fig. 8). Most importantly, our data provide an explanation of how IVM reduces the plasma levels of NS1 in infected patients, which we validated in the same cohort (18). DENV infection induces the UPR (26 – 28), which results in the activation of several transcription factors, including ATF6 and sXBP1. These factors are transported into the nucleus (29) via the importin-α/KPNB1 complex, as demonstrated in this study, and induce the activation of several genes involved in protein folding, including the ER-resident chaperone GRp78. This chaperone promotes the folding of luminal glycoproteins, including NS1. Properly folded NS1 on the one hand, is secreted into the extracellular medium, and on the other hand, it promotes DENV replication (Fig. 8, left panel). Impairment of nuclear transport via IVM treatment or KPNB1 KD reduces nuclear import of UPR-induced transcription factors, hence reducing the abundance of chaperones such as GRp78 or calreticulin. As a result, accumulated misfolded proteins, including NS1 are less efficiently secreted and might only insufficiently promote DENV replication (Fig. 8, right).

It has been reported that IVM blocks DENV NS3 helicase activity *in vitro* (34) and DENV NS5 nuclear transport (35). Our data reveal an additional, not mutually exclusive, mode of action of IVM on DENV replication: impairing proper folding of NS1 that is important for both DENV RNA replication and production of infectious virus particles (6). In addition, NS1 is essential for the formation of vesicle packets, the DENV replication organelle (5), which might also be affected by IVM.

Our results are consistent with a recent clinical trial demonstrating that IVM accelerates NS1 plasma clearance without impacting viremia in DENV-infected patients (18). Here, we provide several lines of evidence showing that IVM impairs NS1 secretion with no or little impact on DENV replication. Previous studies indicated that IVM could block DENV replication, but these data are not necessarily contradictory to ours, as in these former studies used IVM concentrations were much higher, or treatment duration was longer, or different cell types were used (19, 34 – 36). Another discrepancy arises from reported *in vitro* antiviral effects on the one hand and the lack of impact of IVM on viral load in patients. This discrepancy might be explained by the difference in used IVM doses but also by different timing of drug treatment. While in cell culture experiments, cells are often treated at early time points after infection, treatment of patients mostly occurs when viral replication is at its peak or already declining. Consistently, it was shown for yellow fever virus that the addition of IVM 22 h post-infection of cultured cells no longer exerted an antiviral effect (34).

While we could observe an effect of IVM in both DENV-infected A549 and Huh7 cells, in the context of sole NS1 expression, we could only detect an inhibitory effect of IVM in A549, but not Huh7 cells. The underlying reason for this difference is not known, but we note that basal expression of Grp78 was sensitive to IVM in A549 but not Huh7 cells (Fig. 5F and G). Moreover, we observed different degrees of glycosylation of NS1 secreted from A549 vs Huh7 cells (compare eNS1 in Fig. 1D or Fig. 1G and H). These results argue for different folding/maturation of NS1 in these two cell lines or different kinetics of NS1 secretion. Moreover, it was shown that secreted NS1 is associated with neutral lipids (14) and can bind to high-density and low-density lipoproteins (37). Since Huh7 cells efficiently secrete lipoproteins and have a higher lipid metabolism than A549 cells, more efficient lipoprotein loading of NS1 by Huh7 cells might account for the difference to A549 cells.

In our study, we also determined the DENV NS1 interactome and identified 12 cellular proteins as significantly enriched (Fig. 4B), among them a high number of chaperones. We could confirm the interaction of NS1 with proteins of the two main chaperone systems in the ER (38): the classical chaperone system that includes GRp78 and carbohydrate-binding proteins such as calnexin or calreticulin. While these interactors have already been reported (23, 24), here we identified novel ER-resident chaperones specifically associated with NS1, including the oxidoreductases PDIA3 or P4HB, consistent with the presence of 12 conserved cysteine residues forming disulfide bonds in NS1 (39). Furthermore, we identified another folding cofactor belonging to a different class of proteins, CCT8, the chaperonin-containing tailless polypeptide 1 complex. This complex consists of two stacked rings, with a central cavity allowing the folding of proteins in an ATP-dependent manner (40). Interestingly, other CCT proteins were also identified as NS1 interaction partners (24), reinforcing the contribution of this complex to NS1 folding.

Our report also highlights a new role of UPR in the DENV life cycle and pathogenesis. It is well known that DENV infection induces UPR and regulates this pathway for its own benefit (41), although the precise mechanism remains elusive. UPR is required for DENV-induced autophagy (42, 43) and reported to prevent host cell apoptosis and promote viral replication (42). In addition, evasion from innate immunity was also shown to depend on the UPR-autophagy pathway (44). An additional role reported here is the contribution of UPR to DENV replication and pathogenesis by increasing the level of chaperones involved in proper NS1 folding.

In conclusion, our study unveils that nuclear transport of UPR-induced transcription factors is required for proper DENV NS1 folding and secretion. This pathway is targeted by IVM explaining the accelerated plasma clearance of NS1 in DENV-infected patients. We show that this effect is not limited to DENV NS1 but applies to a non-viral glycoprotein, thus highlighting a general cellular pathway linking nuclear transport with the folding and secretion of luminal glycoproteins.

## MATERIALS AND METHODS

### Cell line and culture conditions

The mammalian cell lines Huh7, HEK293T, Vero clone E6, and A549 were grown in Dulbecco’s modified Eagle medium (DMEM; Invitrogen, Karlsruhe, Germany) supplemented with 2 mM l-glutamine, nonessential amino acids, 100 U/mL penicillin, 100 mg/mL streptomycin, and 10% fetal calf serum in a 37°C incubator with 5% CO_2_. The stem cell–derived immortalized hepatocyte-like cells (imHC) were grown in Dulbecco’s modified Eagle medium/nutrient mixture F-12 (DMEM/F-12; Invitrogen) supplemented with 10% fetal bovine serum (FBS; Invitrogen) in a 37°C incubator with 5% CO_2_. The Aedes albopictus clone C6/36 was maintained in Leibovitz’s L-15 medium supplemented with 10 mM HEPES (pH 7.4), 2 mM l-glutamine, nonessential amino acids, 100 U/mL penicillin, 100 mg/mL streptomycin, and 10% fetal calf serum at 28°C.

### Viruses

The molecular clone of the DENV isolate 16681 modified by an in-frame deletion of 97 amino acids in NS1 and encoding a Renilla luciferase (RLuc) reporter gene has been reported previously (45). The cloned genome of the DENV isolate New Guinea C (NGC) was a kind gift from Dr. Andrew Davidson, University of Bristol, United Kingdom (46). All virus stocks were prepared by amplification in C6/36 cells, and virus titers were determined by plaque assay using Vero E6 cells. Stocks of the DVR2ApΔNS1 virus containing an in-frame deletion of 97 amino acids in NS1 were produced in VeroE6 helper cells stably expressing NS1.

### siRNA-based knockdown (KD)

siRNA of 5 pmol (for KPNB1 and GRp78) or 10 pmol (for ATF6, XBP1, and PERK) were mixed with 1 µL of RNAiMAX Reagent (Thermo Fisher Scientific) in 100 µL of OptiMEM (Thermo Fisher Scientific). This mix was incubated 20 min at room temperature (RT) and spotted into a well of 24-well plates. Target A549 or Huh7 were seeded at a density of 4 × 10^4^ cells per well.

### NS1 detection assay

Forty-eight hours post siRNA transfection, cells were infected at an MOI = 2 with the DENV-NGC strain. After 1 h, cells were washed twice with OptiMEM and the medium was replaced by OptiMEM. In the case of ivermectin treatment, the medium was replaced by OptiMEM containing either DMSO or ivermectin (1 µM). In the case of A549 or Huh7 stably expressing NS1 (A549/DENV NS1 or Huh7/DENV NS1, respectively), cells were subjected to the same protocol but without infection. Forty-eight hours post medium change, supernatants were harvested and clarified by centrifugation at 700 × *g* for 5 min. Supernatants were transferred to a new tube containing a final concentration of 1% Triton X-100. Cell were washed with phosphate-buffered saline (PBS) and lysed in lysis buffer (20 mM Tris [pH 7.5], 1% Triton X-100, 0.05% sodium dodecyl sulfate, 150 nM NaCl, 0.5% Na deoxycholate) supplemented with protease/phosphatase inhibitor cocktail (Roche) and Benzonase (Merck-Millipore) for western blot analysis. For short-term treatment with ivermectin, proteins in the supernatants were precipitated prior to western blot analysis as described (47). 10% final concentration of trichloroacetic acid (TCA) was added to the sample and incubated for 15 min at −20°C before centrifugation at 10,000 × *g* at 4°C for 10 min. Pellet was washed with ice-cold acetone before resuspension in 1× PBS. To consider variations in precipitation efficiency, samples were spiked with equal amounts of recombinant SARS-CoV-2 (severe acute respiratory syndrome coronavirus 2) S-protein prior to TCA precipitation and used for normalization. Level of NS1 in the supernatant was also quantified by using the Platelia Dengue NS1 ELISA (Bio-Rad). The range of linearity was determined by using recombinant DENV NS1 (R&D Systems).

### DENV infection and NS1 expression in imHC

The imHC was infected for 2 h at an MOI = 0.1 with DENV-2 (laboratory strain 16681) or transfected for 4–6 h with 2 µg of pCAGGS-E28-NS1D2 plasmid (which contains a signal sequence of last 28 amino acids of envelope protein and full-length sequence of NS1 from DENV2 16681 strain) in OptiMEM. After infection or transfection, cells were washed twice with DMEM/F-12 and cultured in the medium supplemented with 10% FBS containing either DMSO or ivermectin (5 µM) for 48 h. Following the 48-h treatment, supernatants and cells were collected for subsequent NS1 or DENV RNA analyses. In some experiments, the DENV-infected imHC cells were also harvested at 12, 24, and 36 h after drug treatment for western blot analysis.

### Secreted alkaline phosphatase (SEAP) quantifications

A549 cells were reversed transfected with siRNA and a SEAP-encoding lentiviral vector. After 48 h, cells were washed twice with OptiMEM and the medium was replaced by OptiMEM. In the case of ivermectin treatment, the medium was replaced by OptiMEM containing either DMSO or ivermectin (1 µM). Forty-eight hours after medium change, supernatants and cell lysates were analyzed for NS1 detection. For activity measurement, cell lysates and supernatants were analyzed using the colorimetric Secreted Alkaline Phosphatase Reporter Assay Kit (Novus Biologicals) according to the manufacturer’s instructions.

### Indirect immunofluorescence analysis

A549 cells were grown on glass coverslips, transfected/infected as described above and 48 h later, cells were fixed with 4% paraformaldehyde (AppliChem GmbH, Darmstadt, Germany) for 20 min. Cells were permeabilized with 0.1% (vol/vol) Triton X-100 in PBS, incubated with primary antibodies in 1% BSA/PBS, washed, and stained with the corresponding fluorescent secondary antibody. Coverslips were mounted onto glass slides with DAPI-Fluoromount-G (Southern BioTech), and images were acquired using a Leica confocal SP8. Images were analyzed and quantified using the ImageJ software package.

### Determination of the NS1 interactome by quantitative LC-MS/MS proteomics

Samples were generated as previously described (5). Briefly, Huh7 cells stably expressing NS1-HA or NS1 were infected with DVR2A^ΔNS1^. Cells were washed twice with PBS, lysed in immunoprecipitation (IP) lysis buffer (150mM NaCl, 50mM Tris-HCl pH 7.4, and 0.5% Triton X-100), supplemented with protease/phosphatase inhibitor cocktail (Roche) for 1h on ice. Lysates were centrifuged at 21,000 × *g* for 1 h. Pre-cleared cell lysates were added to HA-specific agarose beads slurry (Sigma-Aldrich) and incubated for 3–5 h at 4°C with gentle agitation. Proteins bound to the resin were washed extensively with IP lysis buffer and three times in lysis buffer without detergent and protease inhibitors. Bound proteins were denatured by incubation in 20 µL of guanidinium chloride buffer (600 mM GdmCl, 1 mM Tris[2-carboxyethyl] phosphine-HCl, 4 mM chloroacetamide, and 100 mM Tris-HCl [pH 8.0]). After digestion with 1 µg LysC (WAKO Chemicals, USA) at RT for 3 h, the suspension was diluted in 100 mM Tris-HCl (pH 8.0), and the protein solution was digested with trypsin (Promega) overnight at RT. Peptides were purified on stage tips with three C18 Empore filter discs (3M; Maplewood) and analyzed by liquid chromatography (LC) coupled to mass spectrometry (MS) on an OrbiTrap XL instrument (Thermo Fisher Scientific) as described previously (48). Raw MS data were processed with MaxQuant software version 1.5.3 (49) using the built-in Andromeda search engine to search against the human proteome (UniProtKB, release 2012_01) containing forward and reverse sequences concatenated with the Dengue virus polyprotein (UniProt ID: P-29990) with the individual viral open reading frames manually annotated, and the label-free quantitation algorithm as described previously (50). Additionally, the intensity-based absolute quantification (iBAQ) algorithm and Match Between Runs option were used. In MaxQuant, carbamidomethylation was set as fixed and methionine oxidation and *N*-acetylation as variable modifications, using an initial mass tolerance of 6 ppm for the precursor ion and 0.5 Da for the fragment ions. Search results were filtered with a false discovery rate (FDR) of 0.01 for peptide and protein identifications.

Perseus software version 1.5.3.0 was used to further process the data. Protein tables were filtered to eliminate the identifications from the reverse database and common contaminants. In analyzing MS data, only proteins identified on the basis of at least one peptide and a minimum of three quantitation events in at least one experimental group were considered. iBAQ protein intensity values were normalized against the median intensity of each sample (using only peptides with recorded intensity values across all samples and biological replicates) and log-transformed, and missing values were filled by imputation with random numbers drawn from a normal distribution calculated for each sample. Significant interactors were determined by multiple equal variance *t*-tests with permutation-based FDR statistics. We performed 250 permutations, and the FDR threshold was set at 0.05. The parameter S0 was set at 1 to separate background from specifically enriched interactors. Results were plotted as Volcano plot and heat map using Perseus (51).

### RNA quantification by RT-qPCR

Cells were lysed using lysis buffer LBP contained in the NucleoSpin RNA Plus extraction kit (Macherey-Nagel). Total cellular RNA from one well of a 24-well plate was isolated using the NucleoSpin RNA Plus extraction kit as recommended by the manufacturer. cDNA was generated using the High Capacity cDNA RT kit (Thermo Fisher Scientific). qPCR was performed using the iTaq Universal SYBR Green Master Mix (Bio-Rad) and primers described above. Relative abundance of DENV, KPNB1, or GRp78 mRNA was normalized to hypoxanthine hosphoribosyltransferase (HPRT) transcript level.

### Western blot analysis

Cell lysates or supernatants were mixed with Laemmli buffer and incubated for 5 min at 95°C. In the case of non-reducing conditions, cell lysates were mixed with Laemmli buffer lacking β-mercaptoethanol and incubated for 5 min at 95°C. Proteins were separated by electrophoresis into SDS-10% polyacrylamide gels and transferred to a polyvinylidene fluoride (PVDF) membrane. Membranes were blocked with PBS-Tween 0.1% and supplemented with 5% milk. Primary antibodies (see Table 1 for detail) were diluted in PBS-Tween 0.1% containing 5% milk, and membranes were incubated with the antibodies overnight at 4°C. After several washings, membranes were incubated for 1 h at RT with the corresponding secondary antibody. Signals were detected using the western blot lightning plus-ECL reagent (PerkinElmer) and an Intas ChemoCam Imager 3.2 (Intas). Images were quantified using the ImageJ software package.

**TABLE 1:** Key resource table^*a*^

Reagent or resource	Source	Identifier
Antibodies		
Importin beta-1 specific monoclonal antibody (3E9)	Thermo Fisher Scientific	Cat # MA3-070
KPNB1 specific polyclonal antibody	Thermo Fisher Scientific	Cat #PA5-83110
Dengue virus type 2 NS1 specific polyclonal antibody	Thermo Fisher Scientific	Cat #PA5-32207
Mouse monoclonal anti-GAPDH antibody (G-9)	Santa Cruz Biotechnology	Cat #sc-365062
Rabbit anti-DENV NS1 antibody	(52)	NA
Rabbit anti-DENV NS3 antibody	(53)	NA
Mouse monoclonal anti-DENV NS1 antibody (DN3)	abcam	Cat #ab41616
Mouse monoclonal anti-Grp78 antibody	Thermo Fisher Scientific	Cat #MA527686
Alkaline phosphatase specific polyclonal antibody	Thermo Fisher Scientific	Cat # 200-4135-0100
BiP specific rabbit monoclonal antibody (C50B12)	Cell Signaling	Cat #3177S
Calnexin specific polyclonal antibody	Enzo life sciences	Cat #ADI-SPA-860-F
Calreticulin specific rabbit monoclonal antibody (D3E6) XP	Cell Signaling	Cat #12238S
Dengue virus anti-NS3 antibody (GT2811)	Genetex	Cat #GTX629477
Anti-XBP1 antibody	Novus biologicals	Cat # NBP1-77681
Goat anti-rabbit IgG, horseradish peroxidase conjugated	Sigma	Cat.#A6154
Goat anti-mouse IgG, horseradish peroxidase conjugated	Sigma	Cat.#A4416
Alexa Fluor 488-conjugated donkey anti-rabbit IgG	Thermo Fisher Scientific	Cat.#A-21206
Alexa Fluor 568-conjugated donkey anti-mouse IgG1	Thermo Fisher Scientific	Cat.#A-21124
Alexa Fluor 647-conjugated donkey anti-mouse IgG2a	Thermo Fisher Scientific	Cat.#A-21241
Virus strains		
Dengue DVR2A^pΔNS1^	(6)	NA
DENV isolate New Guinea C (NGC)	Gift from Dr. Andrew Davidson	NA
Chemicals, peptides, and recombinant proteins		
Ivermectin	Sigma-Aldrich	Cat# 8898
Thapsigargin	Focus Biomolecules	Cat#10-2105
Endo H	NEB	Cat# P0702L
Peptide:N-glycosidase F PNGase F (glycerol free), recombinant	NEB	Cat# P0709L
DAPI-Fluoromount-G	Southern BioTech	Cat.#0100-20
Recombinant DENV NS1	R&D Systems	Cat #9439-DG-100
Critical commercial assays		
CellTiter-Glo Luminescent Cell Viability Assay	Promega	Cat#G7571
Lipofectamine RNAiMAX Reagent	Thermo Fisher Scientific	Cat#13778
NucleoSpin RNA Plus extraction kit	Machery-Nagel	Cat#740984
High-Capacity cDNA Reverse Transcription Kit	Thermo Fisher Scientific	Cat#4368814
iTaq Universal SYBR green supermix	BioRad	Cat#1725121
Secreted Alkaline Phosphatase Reporter Assay Kit (Colorimetric)	Novus biologicals	Cat #NBP2-25285
Experimental models: cell lines		
A549 (male)	ATCC	Cat #CCL-185
HEK293T (female)	ATCC	Cat #CCL-3216
C6/36 (sex not defined)	ATCC	Cat.#CRL1660
Vero clone E6 (female)	ATCC	Cat.#CRL1586
Huh7 (male)	(54)	NA
Resources		
Huh7/DENV NS1-HA	(6)	NA
Huh7/DENV NS1	(6)	NA
A549/DENV NS1	This Paper	NA
Oligonucleotides		
siRNA		
ON-TARGETplus Human HSPA5 (3309) siRNA-SMARTpool	Dharmacon	Cat # L-008198-00-0005
ON-TARGETplus Human KPNB1 (3837) siRNA-Individual, 5 nmol	Dharmacon	Cat # J-017523-08-0005
ON-TARGETplus Nontargeting Pool, 5 nmol	Dharmacon	Cat # D-001810-10-05
ON-TARGETplus Human EIF2AK3 siRNA-SMARTpool	Dharmacon	Cat # L-004883-00-0005
ON-TARGETplus Human calreticulin siRNA-SMARTpool	Dharmacon	Cat # L-008197-00-0005
Negative control siPOOL 5	siTOOLS BIOTECH	NA
5 nmol siPOOL 22926 - ATF6 (human)	siTOOLS BIOTECH	NA
5 nmol siPOOL 7494 - XBP1 (human)	siTOOLS BIOTECH	NA
Primers		
hXBP1.3S AAACAGAGTAGCAGCTCAGACTGC	Sigma-Aldrich	NA
mXBP1.12AS* TCCTTCTGGGTAGACCTCTGGGA	Sigma-Aldrich	NA
HPRT Forward CCTGGCGTCGTGATTAGTG	Sigma-Aldrich	NA
HPRT Reverse ACACCCTTTCCAAATCCTCAG	Sigma-Aldrich	NA
KPNB1 Forward CTGCTTCCTGAAGCTGCCATCA	Sigma-Aldrich	NA
KPNB1 Reverse CTTCAGCCAGACTGGAGAAAGC	Sigma-Aldrich	NA
HSPA5 Forward CATCACGCCGTCCTATGTCG	Sigma-Aldrich	NA
HSPA5 Reverse CGTCAAAGACCGTGTTCTCG	Sigma-Aldrich	NA
DENV Forward TACGTGGACCGACAAAGACA	Sigma-Aldrich	NA
DENV Reverse GCTGTTGCACAGTCGACAC	Sigma-Aldrich	NA
RNase P Forward GACAGCCGCTCACCTTGGCT	Integrated DNA Technologies (IDT)	NA
RNase P Reverse GCTACTGGTTTTTCA ATTTCCTGTT	Integrated DNA Technologies (IDT)	NA
Recombinant DNA		
pCMV-Gag-Pol	Gift from Didier Trono	NA
pMD2-VSV-G	Gift from Didier Trono	NA
pSFFV-SEAP	Gift from François-Loïc Cosset	NA
pWPI_puro_NS1	(6)	NA
Software and algorithms		
FIJI	NA	https://imagej.nih.gov/
GraphPad Prism 5.0	La Jolla, CA, USA	GraphPad Software.

^*a*^ NA, not applicable; siRNA, small interfering RNA.

### Lentivirus production

For lentivirus production, HEK293T cells were transfected with the packaging plasmids pCMV-Gag-Pol and pMD2-VSV-G together with pWPI_puro_NS1 or pSFFV-SEAP, encoding for NS1 and SEAP, respectively, by using polyethylenimine (Polysciences Inc.) as reported earlier (6). Two days post-transfection, lentivirus-containing supernatant was harvested and filtered through a 0.45-mm pore-sized filter.

### Detection of NS1 in plasma of dengue patients by ELISA

Levels of NS1 in the plasma of dengue patients were quantified by in-house NS1 ELISA based on purified NS1 standards from each serotype as described (55).

### Detection of Grp78 in plasma of dengue patients

Plasma samples used in this study were collected from 20 adult dengue patients (10 placebo; 10 IVM-treated individuals) on days 3–4 after the initiation of IVM or placebo administration in the clinical trial for IVM as described in reference 18. Plasma sample (0.14 mL) from each patient was used as the starting material for RNA extraction by QIAamp Viral RNA Mini Kit (Qiagen), and the viremia was measured by serotype-specific real-time RT-PCR as previously described (56). For Grp78 evaluation, 0.4 mL of the plasma sample from each corresponding patient was used for total RNA extraction by RNase mini kit with digested DNase I treatment (Qiagen), and then the Grp78 mRNA amounts were quantified by one-step qRT-PCR (Brilliant III-Ultrafast SYBR Green, Agilent) using a LightCycler 480II (Roche). Relative abundance of GRp78 mRNA was normalized to RNase P mRNA level.

### Quantification and statistical analysis

All statistical analyses were performed with the GraphPad Prism 5.0 software package (LaJolla, CA). To assess statistical significance, a one-sample *t*-test or ratio-paired *t*-test was used. Data sets were considered significantly different if the *P* value was less than 0.05. For each experiment, details and sample sizes are listed in the figure legends. Statistical significances are depicted by asterisks in the figures as follows: * for *P* < 0.05, ** for *P* < 0.01, and *** for *P* < 0.001.

## SUPPLEMENTAL MATERIAL

Fig. S1 mbio.01441-23-s0001.tif Impaired NS1 secretion as confirmed by ELISA and upon short-term ivermectin treatment of DENV-infected cells.

Fig. S2 mbio.01441-23-s0002.tif GRp78 knock-down impairs NS1 and SEAP secretion.

Fig. S3 mbio.01441-23-s0003.tif ATF6 knock-down impairs DENV induced GRp78 upregulation.

Supplemental information mbio.01441-23-s0004.docx Legends for supplemental figures and table.

Table S1 mbio.01441-23-s0005.pdf List of significant NS1 interacting proteins.

ASM does not own the copyrights to Supplemental Material that may be linked to, or accessed through, an article. The authors have granted ASM a non-exclusive, world-wide license to publish the Supplemental Material files. Please contact the corresponding author directly for reuse.
