# Supplementary figures and images for: Dengue virus NS1 secretion is regulated via importin-subunit β1 controlling expression of the chaperone GRp78 and targeted by the clinical drug ivermectin
Source: mBio. 2023 Sep 13;14(5):e01441-23. doi: 10.1128/mbio.01441-23 (PMC10653883; doi:10.1128/mbio.01441-23)

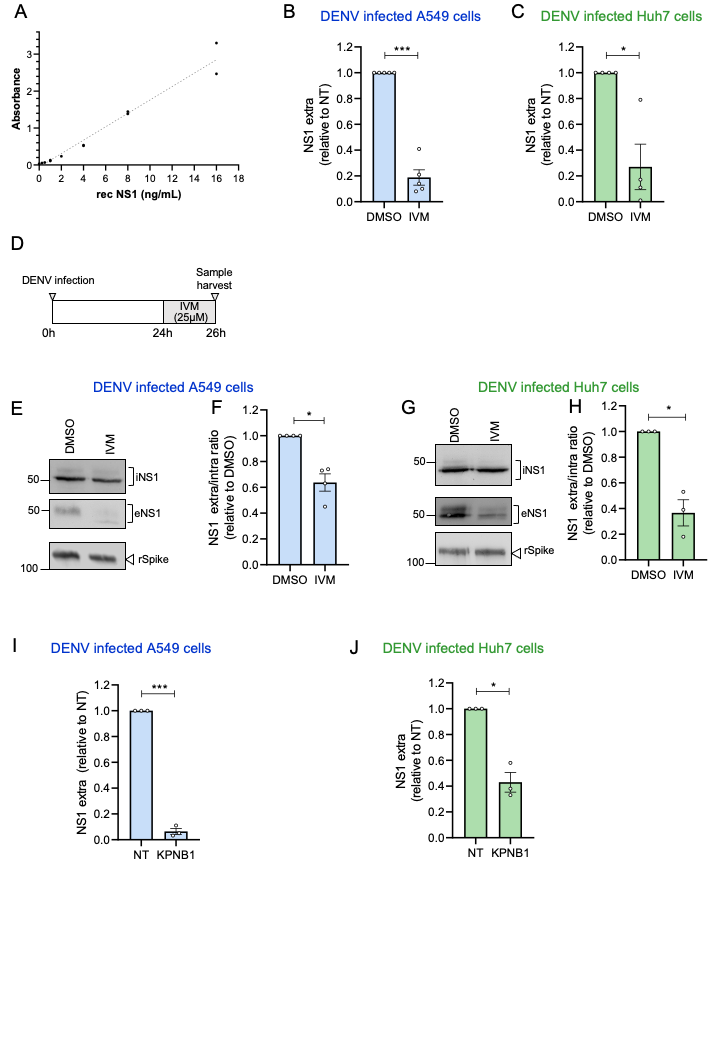

Supplement: Fig. S1 — Impaired NS1 secretion as confirmed by ELISA and upon short-term ivermectin treatment of DENV-infected cells. [file mbio.01441-23-s0001.tif]

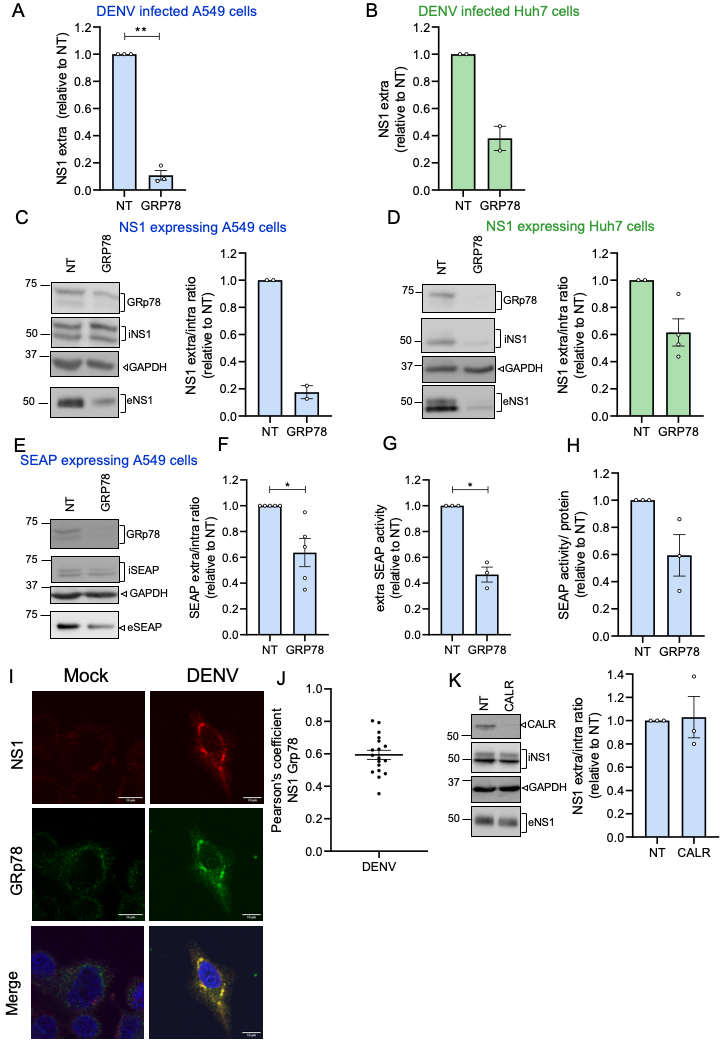

Supplement: Fig. S2 — GRp78 knock-down impairs NS1 and SEAP secretion. [file mbio.01441-23-s0002.tif]

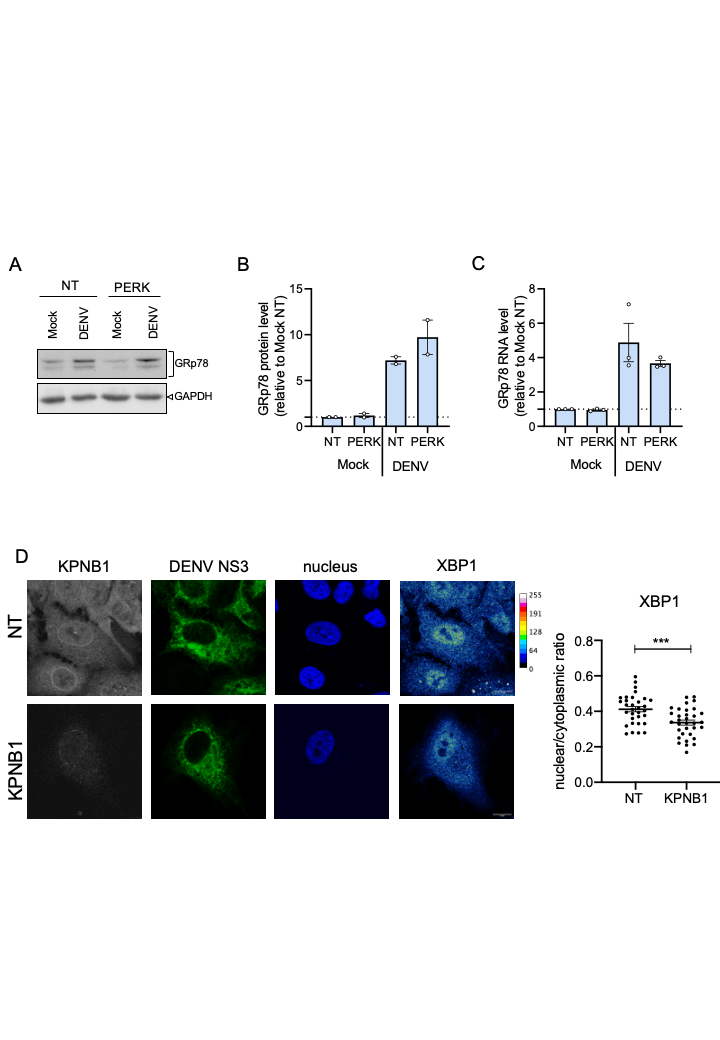

Supplement: Fig. S3 — ATF6 knock-down impairs DENV induced GRp78 upregulation. [file mbio.01441-23-s0003.tif]
