# Supplementary material for: Dengue virus NS1 secretion is regulated via importin-subunit β1 controlling expression of the chaperone GRp78 and targeted by the clinical drug ivermectin
Source: mBio. 2023 Sep 13;14(5):e01441-23. doi: 10.1128/mbio.01441-23 (PMC10653883; doi:10.1128/mbio.01441-23)
Supplement: Supplemental information — Legends for supplemental figures and table. [file mbio.01441-23-s0004.docx]

**Supplemental information**

**Supplemental Figure 1. Impaired NS1 secretion as confirmed by ELISA and upon short-term ivermectin treatment of DENV-infected cells. (A)** Calibration curve of NS1 ELISA. Optical densities obtained with a serial dilution of known concentrations of recombinant NS1 were determined by ELISA. **(B)** Level of NS1 in culture supernatants of DENV infected A549 cells, treated with DMSO or ivermectin (IVM; 1µM) for 48h, was determined by ELISA. Values were normalized to those of DMSO-treated control cells. N=4 biological replicates. **(C)** Same as (A) but using Huh7 cells. **(D)** Summary of the protocol used. A549 or Huh7 cells were infected with DENV (MOI=5) and 24h post infection, cells were washed and treated with ivermectin (IVM; 25µM) or DMSO for 2h. **(E)** Lysate and culture supernatant from Mock and DENV infected A549 cells were analyzed by western blot. NS1 contained in culture supernatant was concentrated by precipitation using trichloroacetic acid (TCA) to allow detection by western blot. **(F)** Quantification of NS1 signals in western blots displayed as the ratio of supernatant/lysate, normalized to DMSO-treated control cells. N=4 biological replicates. **(G)** Same as (E), but using Huh7 cells. **(H)** Same as (F), but using Huh7 cells. N=3 biological replicates. Recombinant SARS-CoV2 Spike, was added prior to TCA precipitation and used to normalize eNS1 amount in order to exclude variation due to different precipitation efficiencies. **(I)** Levels of NS1 in culture supernatants of DENV infected A549 cells treated as in Figure 2B, were determined by ELISA. Values were normalized to those of NT- control cells. N=3 biological replicates. (**J**) Same as (I) but using Huh7 cells. In all graphs, data are represented as mean ± SEM. Each dot in the graphs corresponds to the value of an individual experiment. Statistical significance was determined by one-sample t-test. *** p <0.001, * p < 0.05.

**Supplemental Figure 2. GRp78 knock-down impairs NS1 and SEAP secretion. (A)** A549 cells were transfected with GRp78- or non-targeting (NT) siRNAs prior to DENV infection (MOI=2). After 48h, culture supernatants were harvested and the level of NS1 was determined by ELISA. Data were normalized to those of NT control cells. N=3 biological replicates. **(B)** Same as (A) but using Huh7 cells. N=2 biological replicates. **(C)** NS1 expressing A549 cells were transfected with GRp78-targeting or non-targeting (NT) siRNAs. 48h post transfection, cells were washed, medium was exchanged and cells were incubated for additional 48h. Lysates and culture supernatants of Mock and NS1-expressing cells were analyzed by western blot using NS1-specific antiserum. A representative western blot is shown in the left panel, quantification of NS1 signals on the right. Values are displayed as the ratio of supernatant/lysate, normalized to siNT transfected cells. N=2 biological replicates. **(D)** Same as (C), but using Huh7 samples. N=3 biological replicates. **(E)** A549 cells stably expressing SEAP were transfected with KPNB1-targeting or NT siRNAs. After 48h, cells were washed and incubated for additional 48h. Lysates and culture supernatants from Mock and SEAP expressing cells were analyzed by western blot using alkaline phosphatase-specific antibody. **(F)** Quantification of SEAP displayed as the ratio of supernatant/lysate, normalized to siNT transfected cells. N=5 biological replicates. **(G)** Extracellular SEAP activity released from cells and normalized to siNT transfected cells. N=3 biological replicates. **(H)** Intracellular SEAP activity, normalized to intracellular SEAP amounts as determined by western blot. N=3 biological replicates. **(I)** Immunofluorescence of A549 cells Mock or DENV infected and stained for Grp78 and DENV NS1. **(J)** Signal overlap between Grp78 and NS1 as determined by Pearson’s coefficient, (**K**) A549 cells were transfected with GRp78- or non-targeting (NT) siRNAs prior to DENV infection (MOI=2). After 48h, culture supernatants were harvested and the level of NS1 was determined by western blot using NS1-specific antiserum. Data are normalized to NT condition. N=3 biological replicates. Data are represented as mean ± SEM. Each dot in the graphs corresponds to the value of an individual experiment. Statistical significance was determined by one-sample t-test. ** p <0.01, * p < 0.05.

**Supplemental Figure 3. ATF6 knock-down impairs DENV induced GRp78 upregulation.** **(A)** A549 cells were transfected with PERK-targeting or NT siRNAs for 48h, followed by DENV infection (MOI=2). After 48h, cell lysates were analyzed by western blot for GRP78 expression. N=2 biological replicates. **(B)** Quantification of GRp78 protein level as determined by western blot, normalized to GAPDH signal and Mock-NT signals. **(C)** GRp78 mRNA levels in cells treated as in (A). N=3 biological replicates. Data were normalized to Mock-NT signal and are represented as mean ± SEM. Each dot in graphs corresponds to the value of an individual experiment. **(D)** Immunofluorescence of A549 cells transfected with siRNAs specified on the left and stained for KPNB1, DENV NS3 and XBP1 (left panels). The ratio of nuclear to cytoplasmic pXBP1 was quantified by analyzing 7-9 cells for each of four independent experiments (right panel). Data are represented as mean ± SEM. Statistical significance was determined by unpaired t-test. *** p <0.001.

**Supplemental Table 1. List of significant NS1 interacting proteins.**

This table contains LC-MS/MS data related to affinity-purification coupled to mass spectrometry (AP-MS) of HA-tagged NS1 (NS1-HA) vs. untagged NS1 (NS1 WT) in DV-R2A ΔNS1 infected Huh7 cells. Proteins specifically enriched in NS1-HA are indicated by “+”. UniprotKB accession codes of all protein groups and proteins identified by MS were extracted from UniprotKB (Human; release 2015_08 including isoforms and unreviewed sequences). Protein sequences of DENV-2 16681 strain (P29990) were extracted from UniprotKB.
