# Supplementary material for: Dengue virus NS1 secretion is regulated via importin-subunit β1 controlling expression of the chaperone GRp78 and targeted by the clinical drug ivermectin
Source: mBio. 2023 Sep 13;14(5):e01441-23. doi: 10.1128/mbio.01441-23 (PMC10653883; doi:10.1128/mbio.01441-23)
Supplement: Table S1 — List of significant NS1 interacting proteins. [file mbio.01441-23-s0005.pdf]

T: Protein IDs	T: Majority protein IDs	T: Protein names	T: Gene names	C: Welch's T-test Significant NS1	N: Peptides	N: -Log Welch's T-test p-value NS1 (i.e. SIGNIFICANCE based on p value)	N: Welch's T-test q-value NS1 (i.e. OVERALL STATISTICAL SIGNIFICANCE)	N: Welch's T-test Difference NS1 (i.e. ENRICHMENT)
P29990-4	P29990-4	DENV non-structural protein NS1	NS1	+	17	3,210196598	0	12,71950889
P29990-8	P29990-8	DENV non-structural protein NS4A	NS4A	+	3	5,309036483	0	11,36718186
P27824;B4E2T8;H0Y9Q7;D6RGY2;E7ETH2;D6RFL1;D6RB85;D6RDP7;H0Y9H1;F5H856;D6RAQ8;D6RAU8	P27824;B4E2T8	Calnexin	CANX	+	7	4,529956838	0	7,322527885
O14967;B4DRG2;E7EVY8;D6RAZ4	O14967;B4DRG2;E7EVY8	Calmegin	CLGN	+	8	2,86389025	0	5,81799372
Q562R1	Q562R1	Beta-actin-like protein 2	ACTBL2		8	0,483711847	0,179764706	5,812171141
P29990-9	P29990-9	DENV non-structural protein NS4B	NS4B	+	1	4,040222998	0	5,774119059
O60613	O60613	15 kDa selenoprotein	SEP15	+	2	3,678714366	0	5,187176307
P30101;B3KQT9;G5EA52;F5H119	P30101;B3KQT9;G5EA52;F5H119	Protein disulfide-isomerase A3;Protein disulfide-isomerase	PDIA3	+	7	3,550299577	0	4,570713679
Q9NYU2;Q9NYU2-2	Q9NYU2;Q9NYU2-2	UDP-glucose:glycoprotein glucosyltransferase 1	UGGT1	+	14	2,991583619	0	4,216765324
Q15075	Q15075	Early endosome antigen 1	EEA1	+	2	2,085415522	0,015692308	3,980854034
P27797;B4E2Y9	P27797;B4E2Y9	Calreticulin	CALR	+	7	3,475790846	0	3,857216517
P04259;F5H6G5	P04259;F5H6G5	Keratin, type II cytoskeletal 6B	KRT6B		21	0,770112013	0,131709091	3,624722958
O95399-2;Q5H8X8;A8MXQ0;O95399	O95399-2;Q5H8X8;A8MXQ0;O95399	Urotensin-2	UTS2	+	2	1,900463419	0,030105263	3,461115837
P11021;B4DEF7	P11021	78 kDa glucose-regulated protein	HSPA5	+	29	2,967463739	0,0184	2,852842172
P04406;E7EU54;E7EUT5;E7EUT4;E7EPW1	P04406;E7EU54;E7EUT5;E7EUT4	Glyceraldehyde-3-phosphate dehydrogenase	GAPDH	+	3	2,362499872	0,0286	2,804388046
P78371;F5GWF6;B7ZAT2;F8VQ14	P78371;F5GWF6;B7ZAT2;F8VQ14	T-complex protein 1 subunit beta	CCT2		8	1,74258784	0,060769231	2,698189418
P07237;F5H8J2;B4DUA5;E7EPA8;H0Y3Z3	P07237;F5H8J2;B4DUA5;E7EPA8;H0Y3Z3	Protein disulfide-isomerase	P4HB	+	9	3,249888455	0,01725	2,668820779
P48643;B7ZAR1;E9PCA1;E7ENZ3;E7EWW2;B4DYD8	P48643;B7ZAR1;E9PCA1;E7ENZ3;E7EWW2;B4DYD8	T-complex protein 1 subunit epsilon	CCT5		6	1,187882668	0,085	2,637226184
P46063;F5GYB7;F5H4P4;F5H3W0;F5H2L2;F8WA66;F8WD97	P46063;F5GYB7;F5H4P4;F5H3W0;F5H2L2;F8WA66;F8WD97	ATP-dependent DNA helicase Q1	RECQL		2	0,854301819	0,146060606	2,616040468
P17661	P17661	Desmin	DES		2	0,46415468	0,251259259	2,588612318
P50990;G5E9B2;B4DEM7;B4DQH4	P50990;G5E9B2;B4DEM7;B4DQH4	T-complex protein 1 subunit theta	CCT8	+	15	2,608323008	0,030666667	2,542480469
P40227;B4DPJ8;A6NCD2	P40227;B4DPJ8;A6NCD2	T-complex protein 1 subunit zeta	CCT6A		6	1,861036672	0,061333333	2,513172944
P50991;B7Z9L0;F5H5W3;B7Z2F4	P50991;B7Z9L0;F5H5W3;B7Z2F4	T-complex protein 1 subunit delta	CCT4		6	1,888976899	0,063612903	2,279141108
Q01081;Q71RF1;Q701P4	Q01081;Q71RF1;Q701P4	Splicing factor U2AF 35 kDa subunit	U2AF1		2	0,907575665	0,164722222	2,239427567
Q96AG4	Q96AG4	Leucine-rich repeat-containing protein 59	LRRC59		2	0,852989446	0,160883721	2,195578972
Q12874;E7EUT8	Q12874;E7EUT8	Splicing factor 3A subunit 3	SF3A3		3	0,31791353	0,395282511	2,166354338
P29990-7	P29990-7	DENV non-structural protein 3	NS3		6	2,653948763	0,057272727	2,154791196
O43143;F5H6K0	O43143;F5H6K0	Pre-mRNA-splicing factor ATP-dependent RNA helicase DHX15	DHX15		2	1,416475094	0,099545455	2,138311942
Q99832;B7Z4T9;F8WAM2;F5GZK5;B8ZZC9;B7Z4Z7;B7Z1C9;A8MWI8	Q99832;B7Z4T9;F8WAM2;F5GZK5;B8ZZC9;B7Z4Z7	T-complex protein 1 subunit eta	CCT7		6	1,944670249	0,068242424	2,120890458
P62258;P62258-2	P62258;P62258-2	14-3-3 protein epsilon	YWHAE		3	0,805059435	0,178021053	2,075928609
P02679;C9JEU5;C9JC84;P02679-2;C9JPQ9;C9JU00	P02679;C9JEU5;C9JC84;P02679-2;C9JPQ9;C9JU00	Fibrinogen gamma chain	FGG		3	0,919844364	0,162857143	2,038949569
P53999;F5H1H8	P53999;F5H1H8	Activated RNA polymerase II transcriptional coactivator p15	SUB1		4	1,197323296	0,134571429	2,032114506
P49368;E9PAQ6;B4DUR8;P49368-2;E9PRC8;Q5SZX9;Q5SZX6;E9PQ35;E9PM09;Q5SZW8	P49368;E9PAQ6;B4DUR8;P49368-2;E9PRC8	T-complex protein 1 subunit gamma	CCT3		6	1,006467365	0,169546667	1,975945234
P55157;D6REL9;F5H6U4;E9PBP6;Q6P5T3	P55157;D6REL9;F5H6U4;E9PBP6;Q6P5T3	Microsomal triglyceride transfer protein large subunit	MTTP		2	1,22972727	0,137806452	1,914460659
Q9UBM7;E9PJ54;E9PMT8;E9PM00;E9PRL8;E9PQ71;E9PIP9	Q9UBM7;E9PJ54;E9PMT8;E9PM00;E9PRL8;E9PQ71;E9PIP9	7-dehydrocholesterol reductase	DHCR7		2	1,578595753	0,122415094	1,785657088
P07437;Q5JP53;F8VYX6;F8VW92;Q5ST81;F8VUJ7;E7EWR1;E7ENH1;F5H894;E9PBJ4;Q9BUF5;G3V2A3;F5H0I4;A6NKZ8;B3KS31;C9JAA5;Q5SQY0;B4DP54;A6NNZ2;Q9H4B7;CON__ENSEMBL:ENSBTAP00000025008;G3V5W4;G3V2R8;G3V3R4;G3V2N6	P07437;Q5JP53;F8VYX6;F8VW92;Q5ST81;F8VUJ7;E7EWR1;E7ENH1;F5H894;E9PBJ4	Tubulin beta chain	TUBB		16	1,690194716	0,125833333	1,771546364
O95864;O95864-3;B7Z634;O95864-2	O95864;O95864-3	Fatty acid desaturase 2	FADS2		4	0,644569035	0,248613333	1,708260616
P63244;D6RFX4;H0YAF8;D6R9Z1;D6RAC2;H0Y8W2;D6R9L0;D6REE5;D6RAU2;E9PD14;D6RFZ9;D6RDF4;D6RHH4;H0YAM7;D6RGK8;H0Y9P0;D6RHJ5;D6RF23;D6R909;H0Y8R5;D6RBD0	P63244;D6RFX4;H0YAF8;D6R9Z1;D6RAC2;H0Y8W2;D6R9L0;D6REE5;D6RAU2;E9PD14;D6RFZ9;D6RDF4;D6RHH4;H0YAM7	Guanine nucleotide-binding protein subunit beta-2-like 1;Guanine nucleotide-binding protein subunit beta-2-like 1, N-terminally processed	GNB2L1		4	1,011249049	0,178368932	1,653087139
P18124;A8MUD9;C9JIJ5;C9JZ88	P18124;A8MUD9	60S ribosomal protein L7	RPL7		7	0,963386803	0,176	1,628024419
P02671;P02671-2;Q6NSD8	P02671;P02671-2	Fibrinogen alpha chain;Fibrinopeptide A;Fibrinogen alpha chain	FGA		5	1,038483345	0,181544554	1,599814097
P31689;B7Z5C0;F5GZ88	P31689;B7Z5C0;F5GZ88	DnaJ homolog subfamily A member 1	DNAJA1		3	1,723198162	0,1324	1,560741266
P81605;A5JHP3	P81605;A5JHP3	Dermcidin;Survival-promoting peptide;DCD-1	DCD		2	0,533246719	0,325005464	1,541577578
P49411	P49411	Elongation factor Tu, mitochondrial	TUFM		5	1,103479501	0,177942857	1,530108929
P31942;P31942-2;B4DHY1;P31942-4;P31942-3	P31942;P31942-2;B4DHY1;P31942-4;P31942-3	Heterogeneous nuclear ribonucleoprotein H3	HNRNPH3		2	0,539405148	0,325362162	1,504315615
P13667	P13667	Protein disulfide-isomerase A4	PDIA4		5	0,712110175	0,252789809	1,489080588
Q15366;B4DRD7;F8VZX2;G3V0E8;B4DXP5;Q68Y55;Q32Q82;F8VYL7;A8K7X6;Q6IPF4;F8W0G4;F8VXH9;F8W1G6;B4DLC0;F8VRH0;F8VWQ4;F8VRG9	Q15366;B4DRD7;F8VZX2;G3V0E8;B4DXP5;Q68Y55;Q32Q82;F8VYL7;A8K7X6;Q6IPF4;F8W0G4;F8VXH9;F8W1G6;B4DLC0	Poly(rC)-binding protein 2	PCBP2		3	0,418171244	0,385100917	1,420473655
Q6NUP7;Q6NUP7-2	Q6NUP7;Q6NUP7-2	Serine/threonine-protein phosphatase 4 regulatory subunit 4	PPP4R4		1	0,763628436	0,251204969	1,392452319
Q5BKY9;G3XAI9;Q8N9E0;Q5BKY9-2	Q5BKY9;G3XAI9;Q8N9E0;Q5BKY9-2	Protein FAM133B;Protein FAM133A	FAM133B;FAM133A		3	0,280491665	0,519228346	1,370388826
P17987;E7EQR6;F5H282;E7ERF2;F5GZI8;F5GYL4;F5H726;F5H676;F5H136	P17987;E7EQR6;F5H282	T-complex protein 1 subunit alpha	TCP1		9	1,366187624	0,177653846	1,365986983
P10809;E7EXB4;E7ESH4;F5GWR2;B7Z712;C9JL25;C9JL19;C9JCQ4	P10809	60 kDa heat shock protein, mitochondrial	HSPD1		12	1,74190549	0,162776471	1,342950662
P68371;A8K854;Q3ZCM7;F8W813	P68371;A8K854	Tubulin beta-4B chain;Tubulin beta-3 chain	TUBB4B;TUBB3		18	1,542334653	0,178893617	1,327635129
P55081	P55081	Microfibrillar-associated protein 1	MFAP1		4	0,621422799	0,326580645	1,303395351
Q13200;F5GZ16;E9PCS3;E7EW34	Q13200;F5GZ16;E9PCS3;E7EW34	26S proteasome non-ATPase regulatory subunit 2	PSMD2		1	1,144568979	0,220030075	1,272834619
Q8N5F7;Q5M9Q1	Q8N5F7;Q5M9Q1	NF-kappa-B-activating protein;NKAP-like protein	NKAP;NKAPL		1	0,527166086	0,36817561	1,251653035
P60468	P60468	Protein transport protein Sec61 subunit beta	SEC61B		2	1,584031759	0,184075472	1,244970004
Q96CW1;Q96CW1-2;E9PFW3;F5H0H0;C9JJ47;C9JGT8;B4DJB1;C9JJD3;C9JTK4;C9JPV8	Q96CW1;Q96CW1-2;E9PFW3;F5H0H0;C9JJ47;C9JGT8;B4DJB1;C9JJD3;C9JTK4	AP-2 complex subunit mu	AP2M1		11	0,27468511	0,540875472	1,239773909
Q9BUJ2;B7Z4B8;Q9BUJ2-4;Q9BUJ2-2;Q9BUJ2-3;Q9BUJ2-5	Q9BUJ2;B7Z4B8;Q9BUJ2-4;Q9BUJ2-2;Q9BUJ2-3	Heterogeneous nuclear ribonucleoprotein U-like protein 1	HNRNPUL1		16	1,569306162	0,179721739	1,233537674
Q7Z417	Q7Z417	Nuclear fragile X mental retardation-interacting protein 2	NUFIP2		1	0,34884964	0,472991736	1,223907789
P63151;E5RFR9;B4E1T7;Q9Y2T4;Q9Y2T4-3;B7Z3Y1;Q9Y2T4-2	P63151;E5RFR9;B4E1T7;Q9Y2T4;Q9Y2T4-3;B7Z3Y1;Q9Y2T4-2	Serine/threonine-protein phosphatase 2A 55 kDa regulatory subunit B alpha isoform;Serine/threonine-protein phosphatase 2A 55 kDa regulatory subunit B gamma isoform	PPP2R2A;PPP2R2C		2	0,628211121	0,340375	1,217212756
Q9Y265;Q9Y265-2	Q9Y265;Q9Y265-2	RuvB-like 1	RUVBL1		1	0,96019124	0,251773585	1,206852913
Q15436;G3V531;E7ES38;F5H6C4;F5H365;B3KXI2	Q15436;G3V531;E7ES38;F5H6C4;F5H365;B3KXI2	Protein transport protein Sec23A	SEC23A		3	1,984136034	0,179526882	1,20339489
Q13283;F8W7N6;E5RH00;E5RI46;E5RIF8;E5RH42;E5RJU8;F5H4D6;Q5HYE9	Q13283;F8W7N6;E5RH00;E5RI46;E5RIF8;E5RH42;E5RJU8;F5H4D6;Q5HYE9	Ras GTPase-activating protein-binding protein 1	G3BP1		2	0,234666342	0,577540636	1,188626766
P09012	P09012	U1 small nuclear ribonucleoprotein A	SNRPA		3	0,767952313	0,322111111	1,1616141

P68363;Q9BQE3;Q71U36;F5H5D3;G3V1U9;P68366;A8MUB1;Q13748;E9PGK3;Q6PEY2;F8VVB9;F8VXZ4;Q13748-2;F8VQQ4;F8VRZ4;F8VS66;F8VWX4;E7ET24;Q9NY65;F8VWV9;C9J2C0;B3KPW9;F8VX09;C9JDL2;C9JEV8;C9JQ00;C9JJQ8;A6NHL2;C9K0S6;F8VRK0;C9JDS9;A6NHL2-2;F8VXZ7;F8VS94;F8VYI2;F8VXB8;F8W0F6	P68363;Q9BQE3;Q71U36;F5H5D3;G3V1U9;P68366;A8MUB1;Q13748;E9PGK3;Q6PEY2;F8VVB9;F8VXZ4;Q13748-2;F8VQQ4	Tubulin alpha-1B chain;Tubulin alpha-1C chain;Tubulin alpha-1A chain;Tubulin alpha-4A chain;Tubulin alpha-3C/D chain;Tubulin alpha-3E chain	TUBA1B;TUBA1C;TUBA1A;TUBA4A;TUBA3C;TUBA3E		14	1,667442062	0,200935484	1,135484536
P12004	P12004	Proliferating cell nuclear antigen	PCNA		3	0,448181911	0,419051724	1,133954922
P17066;P48741;E7EP11;C9IYI3;B4DHP5	P17066;P48741	Heat shock 70 kDa protein 6;Putative heat shock 70 kDa protein 7	HSPA6;HSPA7		3	1,597116363	0,205068702	1,124730905
F8WES6;P01781;F5H3H4;F5GXM8;P01763;P01767;P01765;P01764;P01766;P01771;P01768;P01762;P01772	F8WES6;P01781	Ig heavy chain V-III region GAL			3	0,909839677	0,305372093	1,106097857
P49327;F5H0D4	P49327	Fatty acid synthase;[Acyl-carrier-protein] S-acetyltransferase;[Acyl-carrier-protein] S-malonyltransferase;3-oxoacyl-[acyl-carrier-protein] synthase;3-oxoacyl-[acyl-carrier-protein] reductase;3-hydroxyacyl-[acyl-carrier-protein] dehydratase;Enoyl-[acyl-carrier-protein] reductase;Oleoyl-[acyl-carrier-protein] hydrolase	FASN		3	0,628834406	0,377769231	1,061643839
P30040;F8W1G0;F8VY02	P30040	Endoplasmic reticulum resident protein 29	ERP29		3	1,037364984	0,296189349	1,058821599
P37108;H0YLA2	P37108;H0YLA2	Signal recognition particle 14 kDa protein	SRP14		1	0,279989707	0,556321168	1,046920776
Q5VTL8;Q5VTL8-2	Q5VTL8;Q5VTL8-2	Pre-mRNA-splicing factor 38B	PRPF38B		2	1,014118151	0,307098266	1,039398909
P02675;B4E1D3;D6REL8;CON__P02676	P02675;B4E1D3;D6REL8	Fibrinogen beta chain;Fibrinopeptide B;Fibrinogen beta chain	FGB		3	0,910252134	0,328919786	1,016523282
O60237;F8W8M3;O60237-4;O60237-3;Q2TAI8	O60237;F8W8M3;O60237-4;O60237-3;Q2TAI8	Protein phosphatase 1 regulatory subunit 12B	PPP1R12B		2	0,540647829	0,415179039	0,985747973
Q00325;Q00325-2;F8VVM2;F8VZL5;F8VWR4;F8VWQ0	Q00325;Q00325-2;F8VVM2;F8VZL5;F8VWR4;F8VWQ0	Phosphate carrier protein, mitochondrial	SLC25A3		4	1,351961289	0,292190476	0,960269451
Q8IYB3;A9Z1X7;E9PCT1;Q8IYB3-2	Q8IYB3;A9Z1X7;E9PCT1;Q8IYB3-2	Serine/arginine repetitive matrix protein 1	SRRM1		2	0,381006915	0,518440154	0,90978996
Q9BS26	Q9BS26	Endoplasmic reticulum resident protein 44	ERP44		4	0,340526873	0,553695652	0,85439841
Q9UDY2;F5H886;Q9UDY2-3;F5H301;Q9UDY2-2;Q9UDY2-4	Q9UDY2;F5H886;Q9UDY2-3;F5H301	Tight junction protein ZO-2	TJP2		3	0,561390882	0,45815	0,845415354
Q14697;F5H6X6;Q14697-2;E9PKU7;H0YFL4	Q14697;F5H6X6;Q14697-2;E9PKU7	Neutral alpha-glucosidase AB	GANAB		4	0,61497074	0,439254237	0,844121456
Q9NXE8;B4DJK2;Q9NXE8-2	Q9NXE8;B4DJK2;Q9NXE8-2	Pre-mRNA-splicing factor CWC25 homolog	CWC25		2	0,309315383	0,573670175	0,819409847
Q13643	Q13643	Four and a half LIM domains protein 3	FHL3		2	0,561944655	0,485870968	0,781441689
P33993;F5H4S2;F5H776;A4D2A2;P33993-2;C9J8M6	P33993;F5H4S2;F5H776;A4D2A2;P33993-2	DNA replication licensing factor MCM7	MCM7		6	0,73358716	0,441535865	0,771110217
Q05519;Q5T760;Q8IWE6;B4DWT1;B4DTC1;Q5T757	Q05519;Q5T760;Q8IWE6	Serine/arginine-rich splicing factor 11	SRSF11		5	0,599058884	0,484550607	0,757388274
P01009;P01009-2;P01009-3;G3V2B9;G3V544;G3V5R8;G3V387	P01009;P01009-2;P01009-3	Alpha-1-antitrypsin;Short peptide from AAT	SERPINA1		10	0,223237307	0,648652038	0,736470222
Q9UJV9;D6RD33;D6RGI7	Q9UJV9	Probable ATP-dependent RNA helicase DDX41	DDX41		3	0,603989348	0,504111111	0,729291916
P25705;A8K092	P25705;A8K092	ATP synthase subunit alpha, mitochondrial	ATP5A1		11	0,449317859	0,547041199	0,729122639
P62195;A8K3Z3	P62195;A8K3Z3	26S protease regulatory subunit 8	PSMC5		2	0,248274924	0,635656051	0,7241834
Q9NZ01;B3KSQ1;Q9NZ01-2	Q9NZ01;B3KSQ1	Very-long-chain enoyl-CoA reductase	TECR		3	0,455419641	0,548847584	0,721406937
Q06210;Q06210-2	Q06210;Q06210-2	Glutamine--fructose-6-phosphate aminotransferase [isomerizing] 1	GFPT1		2	0,577468989	0,5208	0,711300929
P62826;B5MDF5;F5H018;H0YFC6;B4DV51	P62826;B5MDF5;F5H018;H0YFC6	GTP-binding nuclear protein Ran	RAN		6	0,521543792	0,535251908	0,70331494
P05141;P12236;P12235;Q9H0C2	P05141;P12236;P12235	ADP/ATP translocase 2;ADP/ATP translocase 2, N-terminally processed;ADP/ATP translocase 3;ADP/ATP translocase 3, N-terminally processed;ADP/ATP translocase 1	SLC25A5;SLC25A6;SLC25A4		10	0,444326656	0,555709091	0,698301315
Q92734;G5E9V1;Q05BK6;C9JJP5;C9JUE0;C9JTY3	Q92734;G5E9V1;Q05BK6;C9JJP5;C9JUE0;C9JTY3	Protein TFG	TFG		14	0,584152962	0,519264368	0,695263227
P0C0S5;Q71UI9;C9J386;C9J0D1;A6NFA8;A6NKY0;A6NN01;F5H4B2	P0C0S5;Q71UI9;C9J386;C9J0D1;A6NFA8;A6NKY0;A6NN01;F5H4B2	Histone H2A.Z;Histone H2A.V;Histone H2A	H2AFZ;H2AFV		2	0,126704016	0,761933333	0,683631261
O43175;Q5SZU1;F5H634;F5GYN9	O43175;Q5SZU1;F5H634	D-3-phosphoglycerate dehydrogenase	PHGDH		5	0,379162962	0,577641379	0,672683875
P62879;Q9HAV0;C9JD14;C9JZN1;C9JIS1;C9JXA5	P62879;Q9HAV0;C9JD14;C9JZN1;C9JIS1;C9JXA5	Guanine nucleotide-binding protein G(I)/G(S)/G(T) subunit beta-2;Guanine nucleotide-binding protein subunit beta-4	GNB2;GNB4		2	0,161562682	0,720091954	0,664342086
P68104;Q5VTE0;Q6IQ15;F8W6I0;Q05639;A6PW80;Q5JR01;E7EPH7	P68104;Q5VTE0;Q6IQ15;F8W6I0	Elongation factor 1-alpha 1;Putative elongation factor 1-alpha-like 3	EEF1A1;EEF1A1P5		15	0,652396967	0,517861538	0,659992536
P33778;Q99880;Q93079;Q16778;Q99879;Q99877;Q5QNW6;P58876;O60814;Q8N257;P62807;P23527;P57053;P06899;B4DR52;B4DLA9;Q96A08;Q6DRA6;Q6DN03	P33778;Q99880;Q93079;Q16778;Q99879;Q99877;Q5QNW6;P58876;O60814;Q8N257;P62807;P23527;P57053;P06899;B4DR52;B4DLA9;Q96A08	Histone H2B type 1-B;Histone H2B type 1-L;Histone H2B type 1-H;Histone H2B type 2-E;Histone H2B type 1-M;Histone H2B type 1-N;Histone H2B type 2-F;Histone H2B type 1-D;Histone H2B type 1-K;Histone H2B type 3-B;Histone H2B type 1-C/E/F/G/I;Histone H2B type 1-O;Histone H2B type F-S;Histone H2B type 1-J;Histone H2B;Histone H2B type 1-A	HIST1H2BB;HIST1H2BL;HIST1H2BH;HIST2H2BE;HIST1H2BM;HIST1H2BN;HIST2H2BF;HIST1H2BD;HIST1H2BK;HIST3H2BB;HIST1H2BC;HIST1H2BO;H2BFS;HIST1H2BJ;HIST1H2BA		4	0,389470018	0,583401361	0,64751482
P63173	P63173	60S ribosomal protein L38	RPL38		1	0,726184277	0,518765625	0,642671585
Q9UBS4	Q9UBS4	DnaJ homolog subfamily B member 11	DNAJB11		5	1,005369847	0,481404082	0,632765293
Q9Y5X1;B3KXH8	Q9Y5X1;B3KXH8	Sorting nexin-9	SNX9		2	0,556617033	0,552820513	0,630379518
Q8WVK2;B8ZZ98	Q8WVK2;B8ZZ98	U4/U6.U5 small nuclear ribonucleoprotein 27 kDa protein	SNRNP27		2	0,185632871	0,71340413	0,619401852
P23284	P23284	Peptidyl-prolyl cis-trans isomerase B	PPIB		5	0,371042354	0,598635762	0,608365218
P11142;E9PKE3;E7ET08;P11142-2;E9PNE6;E9PN89;A8K7Q2;E9PLF4;E9PQQ4;E9PQK7;E9PK54;P54652;E9PPY6;E9PS65;E9PN25;E9PI65;E9PM13;B3KUS2;P08107;P34931;H0YIE7;F8W1P1;B4DXY3;E7EQL7;F8WEE3;F8VZJ4;F5H1D3;F5GZ62;Q53FA3;H0YG33;Q5SP16;E7EP94	P11142;E9PKE3;E7ET08;P11142-2;E9PNE6;E9PN89;A8K7Q2	Heat shock cognate 71 kDa protein	HSPA8		25	1,069272716	0,488803213	0,607147853
Q8N4Q1;Q8N4Q1-2	Q8N4Q1;Q8N4Q1-2	Mitochondrial intermembrane space import and assembly protein 40	CHCHD4		1	0,211481276	0,708024169	0,602987687
Q8IYT4;F8WBL0;Q8IYT4-2	Q8IYT4;F8WBL0;Q8IYT4-2	Katanin p60 ATPase-containing subunit A-like 2	KATNAL2		1	0,541298441	0,568742857	0,602432569
Q13162;E9PEN3;A6NG45	Q13162;E9PEN3	Peroxiredoxin-4	PRDX4		5	0,285329891	0,655083591	0,582156976
Q9P2M7;A6PVU7;Q9P2M7-2	Q9P2M7;A6PVU7;Q9P2M7-2	Cingulin	CGN		2	0,278099624	0,654670807	0,580262979
P55072;C9JUP7;C9IZA5;E7EQM8	P55072	Transitional endoplasmic reticulum ATPase	VCP		36	0,453100783	0,588956229	0,577135404
Q15084;B7Z254;F8WA83;B5MCQ5;Q15084-2	Q15084;B7Z254;F8WA83;B5MCQ5;Q15084-2	Protein disulfide-isomerase A6	PDIA6		4	0,64197457	0,56105036	0,575053374
P02787;C9JVG0;CON__Q2HJF0;CON__Q29443;CON__Q0IIK2	P02787	Serotransferrin	TF		14	0,599116811	0,574867133	0,557178179
O60701;B4DN25;O60701-2	O60701;B4DN25;O60701-2	UDP-glucose 6-dehydrogenase	UGDH		1	0,208414962	0,710280702	0,554801782
Q86VP6;Q86VP6-2	Q86VP6;Q86VP6-2	Cullin-associated NEDD8-dissociated protein 1	CAND1		3	0,290266609	0,662920245	0,53716747
Q7L014;D6RJA6;F5GYY8;P31146;H0Y9U3	Q7L014	Probable ATP-dependent RNA helicase DDX46	DDX46		18	0,502399017	0,5970033	0,529503981
P08238;Q58FF7;H0Y6E4;E9PHI1;Q5T9W8;H0Y5S7;E9PHI2;H0Y598;E9PHH7	P08238	Heat shock protein HSP 90-beta	HSP90AB1		20	0,502990021	0,600292359	0,527021408
Q8NHP8;F5H5E2	Q8NHP8;F5H5E2	Putative phospholipase B-like 2;Putative phospholipase B-like 2 32 kDa form;Putative phospholipase B-like 2 45 kDa form	PLBD2		3	0,607698312	0,584054054	0,524534225
P13639;B4DPU3;F5H370;F5GZG3;B4DMC6;Q15029;B4DK30	P13639;B4DPU3;F5H370;F5GZG3	Elongation factor 2	EEF2		15	0,485271595	0,613442623	0,514062723
P02751;P02751-5;F8W7G7;E7ERA1;P02751-12;P02751-10;P02751-6;P02751-9;P02751-14;P02751-13;P02751-8;E9PE77;P02751-3;P02751-11;P02751-7;P02751-15;H0Y7Z1;P02751-2;E9PG29;P02751-4	P02751;P02751-5;F8W7G7;E7ERA1;P02751-12;P02751-10;P02751-6;P02751-9;P02751-14;P02751-13;P02751-8;E9PE77;P02751-3;P02751-11;P02751-7;P02751-15;H0Y7Z1;P02751-2;E9PG29;P02751-4	Fibronectin;Anastellin;Ugl-Y1;Ugl-Y2;Ugl-Y3	FN1		2	0,298776213	0,690674772	0,485126495
P62273;A8MZ73	P62273;A8MZ73	40S ribosomal protein S29	RPS29		1	0,535852942	0,6232	0,477422714

P22392;P22392-2;Q32Q12;O60361;F6XY72	P22392;P22392-2;Q32Q12;O60361;F6XY72	Nucleoside diphosphate kinase B;Nucleoside diphosphate kinase;Putative nucleoside diphosphate kinase	NME2;NME1-NME2;NME2P1		5	0,114723452	0,815893048	0,471248627
P27348;E9PG15;B4DMT8;Q04917;B7Z2E6;H0YB80;B0AZS6;E7EX24	P27348;E9PG15;B4DMT8	14-3-3 protein theta	YWHAQ		3	0,321896023	0,710894895	0,442894618
Q15437;Q5QPE1;Q5QPE2;E9PBH1	Q15437	Protein transport protein Sec23B	SEC23B		3	0,45718426	0,660196923	0,436301549
P58397;P58397-3	P58397;P58397-3	A disintegrin and metalloproteinase with thrombospondin motifs 12	ADAMTS12		2	0,23878419	0,728968661	0,429532051
P35998;B7Z5E2	P35998;B7Z5E2	26S protease regulatory subunit 7	PSMC2		1	0,230746568	0,735235955	0,422493537
P06733;P06733-2	P06733;P06733-2	Alpha-enolase	ENO1		3	0,088741495	0,86101292	0,41614906
Q15942;H0Y2Y8;B4DQR8;B4DQX7;C9IZ41;C9JJK5	Q15942;H0Y2Y8;B4DQR8;B4DQX7	Zyxin	ZYX		9	0,369920879	0,714302671	0,394188722
P53007;B4DP62	P53007;B4DP62	Tricarboxylate transport protein, mitochondrial	SLC25A1		4	0,275711994	0,733144476	0,386767228
P11498;B4DN00;E9PS68;E9PRE7	P11498;B4DN00	Pyruvate carboxylase, mitochondrial	PC		3	0,154973497	0,797185984	0,381825447
P60866;B4DW28;E5RIP1;E5RJX2;G3XAN0	P60866;B4DW28;E5RIP1;E5RJX2;G3XAN0	40S ribosomal protein S20	RPS20		2	1,155116174	0,654566978	0,36372153
Q02539	Q02539	Histone H1.1	HIST1H1A		3	0,1412211	0,822255319	0,352612019
P62873;B1AKQ8;B1AKQ7;B1AKQ9;P16520;F5H100;F5H8J8;F5H0S8;E7EP32;E9PCP0	P62873;B1AKQ8;B1AKQ7;B1AKQ9;P16520;F5H100;F5H8J8;F5H0S8;E7EP32;E9PCP0	Guanine nucleotide-binding protein G(I)/G(S)/G(T) subunit beta-1;Guanine nucleotide-binding protein G(I)/G(S)/G(T) subunit beta-3	GNB1;GNB3;GNB2		2	0,055584176	0,887306024	0,347744465
P38646;B7Z4V2;F5H3L8;D6RJI2;H0YBG6;D6RA73;H0Y8S0	P38646;B7Z4V2;F5H3L8	Stress-70 protein, mitochondrial	HSPA9		17	0,943021685	0,694036364	0,336208026
P30050	P30050	60S ribosomal protein L12	RPL12		2	0,633684159	0,710181818	0,334006786
P31431;B4E1S6	P31431;B4E1S6	Syndecan-4;Syndecan	SDC4		2	0,10775575	0,86015544	0,331762632
Q9NWB6;E7EUG0;Q9NWB6-2	Q9NWB6;E7EUG0;Q9NWB6-2	Arginine and glutamate-rich protein 1	ARGLU1		8	0,448192368	0,736361582	0,31914107
P14625;E9PEX3;Q96GW1;Q58FF3;H0YIV0;F8W026	P14625;E9PEX3	Endoplasmin	HSP90B1		21	0,324510383	0,76567313	0,312438011
Q9UQ80;F8VR77;F8VRZ3;F8VTY8;H0Y3X3;H0YIN7;F8W0A3	Q9UQ80;F8VR77;F8VRZ3;F8VTY8;H0Y3X3;H0YIN7;F8W0A3	Proliferation-associated protein 2G4	PA2G4		2	0,251487892	0,798229508	0,302848101
P04075	P04075	Fructose-bisphosphate aldolase A	ALDOA		4	0,250802569	0,799411444	0,295748234
P26368;P26368-2	P26368;P26368-2	Splicing factor U2AF 65 kDa subunit	U2AF2		3	0,127857246	0,860082474	0,277465343
O95816;B4DXE2	O95816;B4DXE2	BAG family molecular chaperone regulator 2	BAG2		1	0,828862669	0,747631285	0,277000984
Q9BRX2	Q9BRX2	Protein pelota homolog	PELO		1	0,23440154	0,813994667	0,271760305
P14618;P14618-2;B4DUU6;E9PF79;E7EUQ8;E7EUJ4	P14618;P14618-2;B4DUU6;E9PF79;E7EUQ8	Pyruvate kinase PKM	PKM		11	0,178542301	0,840358839	0,271319071
Q9NYL4;F8VU90;B4DWB7	Q9NYL4;F8VU90;B4DWB7	Peptidyl-prolyl cis-trans isomerase FKBP11;Peptidyl-prolyl cis-trans isomerase	FKBP11		1	0,281394626	0,811225806	0,263661385
Q15233;C9JYS8;F5GYZ3;Q8WXF1;C9J4X2;C9IZL7;F5H656;Q8WXF1-2	Q15233;C9JYS8;F5GYZ3	Non-POU domain-containing octamer-binding protein	NONO		4	0,167497441	0,855394256	0,253986359
Q9UJS0;Q546F9;F5GX33	Q9UJS0;Q546F9;F5GX33	Calcium-binding mitochondrial carrier protein Aralar2	SLC25A13		2	0,075197451	0,885640777	0,246681054
O60488;O60488-2;O95573;H0Y9A0	O60488;O60488-2;O95573;H0Y9A0	Long-chain-fatty-acid--CoA ligase 4;Long-chain-fatty-acid--CoA ligase 3	ACSL4;ACSL3		2	0,265276907	0,83237037	0,238740842
O75400;O75400-2;E9PHJ7;F8W9L2;O75400-3;F5H578;H0YG38;Q6NWY9;Q6NWY9-2;Q6NWY9-3	O75400;O75400-2;E9PHJ7;F8W9L2;O75400-3	Pre-mRNA-processing factor 40 homolog A	PRPF40A		14	0,141485887	0,86989313	0,227215608
Q15365	Q15365	Poly(rC)-binding protein 1	PCBP1		5	0,14094384	0,880243038	0,212552706
Q14444;E9PEK6;G3V153;Q14444-2;E9PP31;E9PLA9	Q14444;E9PEK6;G3V153;Q14444-2;E9PP31;E9PLA9	Caprin-1	CAPRIN1		3	0,066663808	0,901346062	0,210757017
Q14974;F5H4R7;B7ZAV6	Q14974;F5H4R7;B7ZAV6	Importin subunit beta-1	KPNB1		3	0,266668973	0,859272727	0,208285093
O95232;B4DJ96;F5H1W9;H0YBV7;H0YAR4;D6RDI2;H0YA81	O95232;B4DJ96;F5H1W9	Luc7-like protein 3	LUC7L3		5	0,150858379	0,879553299	0,208003521
P48735;H0YL11;B4DSZ6;B4DFL2	P48735;H0YL11;B4DSZ6;B4DFL2	Isocitrate dehydrogenase [NADP], mitochondrial	IDH2		4	0,248853023	0,866820513	0,192998091
Q16630;Q16630-2;F8WJN3;C9JGC2;Q16630-3;F8W084	Q16630;Q16630-2;F8WJN3;C9JGC2;Q16630-3	Cleavage and polyadenylation specificity factor subunit 6	CPSF6		3	0,098465078	0,887690073	0,188049157
P60842;Q14240;E7EQG2;Q14240-2;Q9NZE6;E7EMV8;B4E102;B4DKP9;P38919;E9PBH4;F8WE11	P60842;Q14240;E7EQG2;Q14240-2	Eukaryotic initiation factor 4A-I;Eukaryotic initiation factor 4A-II;Eukaryotic initiation factor 4A-II, N-terminally processed	EIF4A1;EIF4A2		7	0,098368356	0,887130435	0,187296708
Q9UHX1-4;Q9UHX1-6;Q9UHX1-2;Q9UHX1;Q9UHX1-3;Q9UHX1-5;E9PQ56;E9PN18;H0YEM1;E9PMU7;E9PL19;H0YCP8	Q9UHX1-4;Q9UHX1-6;Q9UHX1-2;Q9UHX1;Q9UHX1-3;Q9UHX1-5;E9PQ56;E9PN18;H0YEM1;E9PMU7	Poly(U)-binding-splicing factor PUF60	PUF60		11	0,119784328	0,892561576	0,185619195
P36542;B4DL14;P36542-2	P36542;B4DL14;P36542-2	ATP synthase subunit gamma, mitochondrial;ATP synthase subunit gamma	ATP5C1		5	0,144806673	0,888588529	0,183306058
O43809;Q5HYH5	O43809;Q5HYH5	Cleavage and polyadenylation specificity factor subunit 5	NUDT21;DKFZp313O211		4	0,15934674	0,89064	0,177122275
Q06830	Q06830	Peroxiredoxin-1	PRDX1		11	0,170287699	0,896554156	0,176364263
O14773;E7EV34;B5MDC1	O14773;E7EV34;B5MDC1	Tripeptidyl-peptidase 1	TPP1		2	0,137480414	0,891066339	0,175893943
Q96PK6;Q96PK6-2	Q96PK6	RNA-binding protein 14	RBM14		8	0,166900118	0,886883951	0,168585618
P21333;Q5HY54;P21333-2;A6NDY9;E7EWU2;F8WE98;E9PHF0;O75369;Q14315;H0Y5F3;H0Y5C6;O75369-5;O75369-4;O75369-6;O75369-3;O75369-2;Q60FE7;B2ZZ83;Q14315-2	P21333;Q5HY54;P21333-2;A6NDY9	Filamin-A	FLNA		7	0,141158416	0,889803922	0,167486747
P07355;H0YKS4;H0YMD0;H0YMU9;H0YN42;P07355-2;H0YMM1;H0YNP5;H0YM50;A6NMY6;H0YN52;H0YNA0;H0YKZ7;H0YLV6;H0YMT9;H0YKX9;H0YN28;H0YL33;H0YMW4;B3KRQ1;H0YLE2;H0YMD9;H0YKV8;H0YKL9	P07355;H0YKS4;H0YMD0;H0YMU9;H0YN42;P07355-2;H0YMM1;H0YNP5;H0YM50;A6NMY6;H0YN52;H0YNA0;H0YKZ7;H0YLV6;H0YMT9;H0YKX9;H0YN28;H0YL33;H0YMW4;B3KRQ1	Annexin A2;Annexin;Putative annexin A2-like protein	ANXA2;ANXA2P2		4	0,213048276	0,894512563	0,164217949
Q01844;C9JGE3;B0QYK0;Q01844-2;B0QYK1;Q96FE8;Q96MX4	Q01844;C9JGE3;B0QYK0;Q01844-2;B0QYK1;Q96FE8;Q96MX4	RNA-binding protein EWS	EWSR1		3	0,06733837	0,92179669	0,163442135
Q14498;H0Y4X3;E1P5S2;E9PCZ6;A2RRD3;Q14498-2;G3XAC6;Q5QP23;Q86U06;Q86U06-2;Q86U06-4;G3V546;G3V5Z6;Q5QP22;Q5QP21;H0YJJ3;G3XAP0;E7EU28;Q86U06-3;Q86U06-5	Q14498;H0Y4X3;E1P5S2;E9PCZ6;A2RRD3;Q14498-2;G3XAC6	RNA-binding protein 39	RBM39;RNPC2		8	0,152153065	0,888479218	0,163249334
P06753;H0YL80	P06753	Tropomyosin alpha-3 chain	TPM3		18	0,015865201	0,978387387	0,150209347
Q8NC51;Q8NC51-3;Q8NC51-4;Q8NC51-2	Q8NC51;Q8NC51-3;Q8NC51-4;Q8NC51-2	Plasminogen activator inhibitor 1 RNA-binding protein	SERBP1		3	0,081090082	0,920684086	0,145071586
Q8NF91;Q8NF91-4;E7ENN3;E9PEL9;Q8NF91-8;Q8NF91-2;E7EQI5	Q8NF91;Q8NF91-4;E7ENN3;E9PEL9;Q8NF91-8;Q8NF91-2;E7EQI5	Nesprin-1	SYNE1		2	0,110722725	0,91012381	0,136761904
P52597;B4DKS8	P52597;B4DKS8	Heterogeneous nuclear ribonucleoprotein F;Heterogeneous nuclear ribonucleoprotein F, N-terminally processed	HNRNPF		2	0,57398933	0,888865672	0,136618455
P61978;P61978-2;Q5T6W5;P61978-3;B4DFF1;Q5T6W2;Q5T6W1	P61978;P61978-2;Q5T6W5;P61978-3;B4DFF1;Q5T6W2;Q5T6W1	Heterogeneous nuclear ribonucleoprotein K	HNRNPK		10	0,135062202	0,923867299	0,119685173
P08670;F5H288;B0YJC4;B0YJC5;Q5JVS8	P08670;F5H288;B0YJC4;B0YJC5	Vimentin	VIM		5	0,081699517	0,937082353	0,116912842
Q02978;F5GY65	Q02978;F5GY65	Mitochondrial 2-oxoglutarate/malate carrier protein	SLC25A11		1	0,052320691	0,947493088	0,104066372
Q66PJ3;Q66PJ3-2;Q66PJ3-3;F5GYV5;F8WCT1;B3V0L1;Q66PJ3-4;F5H7X4;F8WEP2;Q66PJ3-5	Q66PJ3;Q66PJ3-2;Q66PJ3-3;F5GYV5;F8WCT1;B3V0L1;Q66PJ3-4;F5H7X4	ADP-ribosylation factor-like protein 6-interacting protein 4	ARL6IP4		3	0,03257747	0,964408163	0,090649923
P19474;F5H012;P19474-2;H0YDP8	P19474;F5H012;P19474-2	E3 ubiquitin-protein ligase TRIM21	TRIM21		14	0,059299689	0,949119266	0,088293552
Q9Y383;B7Z500;Q9Y383-2;B7Z4Q3;Q9NQ29;Q9NQ29-2;A8MYV2;Q9NQ29-3;C9JWT3;B8ZZ10;F8WB41;F8WEU3;B8ZZ12;F8WBC1;F2Z322;B8ZZ09	Q9Y383;B7Z500;Q9Y383-2;B7Z4Q3	Putative RNA-binding protein Luc7-like 2;UPF0562 protein C7orf55	LUC7L2;C7orf55		12	0,080338652	0,945802299	0,085277239

P49756;P49756-2;P49756-3;E9PQU5;H0YE46;E9PSE8;P49756-4	P49756;P49756-2;P49756-3	RNA-binding protein 25	RBM25		14	0,049915234	0,963036364	0,069673379
Q9Y262;C9JHP4;C9K0Q7;B0QY89;G8JLH4;B0QY90	Q9Y262;C9JHP4;C9K0Q7;B0QY89;G8JLH4	Eukaryotic translation initiation factor 3 subunit L	EIF3L		3	0,023333501	0,977073826	0,06474328
Q8NEZ4;Q8NEZ4-3;H0YMU7;Q8NEZ4-2	Q8NEZ4;Q8NEZ4-3;H0YMU7;Q8NEZ4-2	Histone-lysine N-methyltransferase 2C	KMT2C		2	0,010764366	0,986079295	0,059634765
O60784;F8WA65;E7EPD0;E7EPZ3;O60784-3;O60784-4;O60784-2	O60784;F8WA65;E7EPD0;E7EPZ3;O60784-3;O60784-4;O60784-2	Target of Myb protein 1	TOM1		2	0,008544474	0,988780702	0,056190888
Q8WXA9;B3KRJ9;Q8WXA9-2	Q8WXA9;B3KRJ9;Q8WXA9-2	Splicing regulatory glutamine/lysine-rich protein 1	SREK1		1	0,0146462	0,988035477	0,054871241
Q01581;Q8N995	Q01581;Q8N995	Hydroxymethylglutaryl-CoA synthase, cytoplasmic	HMGCS1		1	0,044311811	0,978822472	0,047803164
P08579;B4DDC7	P08579;B4DDC7	U2 small nuclear ribonucleoprotein B	SNRPB2		2	0,032130698	0,980553571	0,046616395
P63104;E7EX29;E5RGE1;E5RIR4;E9PD24;E7EVZ2;E7ESK7	P63104;E7EX29;E5RGE1;E5RIR4;E9PD24;E7EVZ2;E7ESK7	14-3-3 protein zeta/delta	YWHAZ		2	0,028651003	0,983625835	0,045677185
P42677;Q5T4L4	P42677;Q5T4L4	40S ribosomal protein S27	RPS27		3	0,114697999	0,976959641	0,038934231
P27635;F8W7C6	P27635;F8W7C6	60S ribosomal protein L10	RPL10		2	0,032360531	0,988968889	0,03494517
P53621;P53621-2	P53621;P53621-2	Coatomer subunit alpha;Xenin;Proxenin	COPA		2	0,027428647	0,986327434	0,033449491
P55735;E9PHR5;A8MTA5;A8MWR8;A8MXL6;A8MV37	P55735;E9PHR5;A8MTA5;A8MWR8;A8MXL6;A8MV37	Protein SEC13 homolog	SEC13		2	0,024615564	0,985916484	0,028179328
P46776;E9PJD9;E9PLL6;E9PLX7	P46776;E9PJD9;E9PLL6;E9PLX7	60S ribosomal protein L27a	RPL27A		2	0,007857928	0,99835141	0,012385686
Q13247;Q13247-3;Q08170;E7EMJ8	Q13247;Q13247-3	Serine/arginine-rich splicing factor 6	SRSF6		3	0,00345082	0,998172414	0,008395831
Q8N9Q2	Q8N9Q2	Protein SREK1IP1	SREK1IP1		3	0,003897586	0,997823656	0,005491416
P31943;E9PCY7;G8JLB6;D6RIU0;D6RBM0;E5RGH4;E7EQJ0;D6RAM1;E5RGV0;D6R9T0;D6RFM3;D6RIT2;D6RDU3;D6RJ04;D6RIH9;P55795;H0YAQ2;E7EN40;H0YB39;D6RF17;D6RDL0;F5GZT4;H0YBG7;H0YBD7;B4DFK9	P31943;E9PCY7;G8JLB6;D6RIU0;D6RBM0;E5RGH4;E7EQJ0;D6RAM1;E5RGV0;D6R9T0;D6RFM3;D6RIT2;D6RDU3;D6RJ04;D6RIH9	Heterogeneous nuclear ribonucleoprotein H;Heterogeneous nuclear ribonucleoprotein H, N-terminally processed	HNRNPH1		5	0,005208189	0,997939914	0,002882799
P04843;F8WF32;F5H6I5	P04843;F8WF32;F5H6I5	Dolichyl-diphosphooligosaccharide--protein glycosyltransferase subunit 1	RPN1		1	-4,34E-08	1	0
P07307;P07307-3;P07307-2	P07307;P07307-3;P07307-2	Asialoglycoprotein receptor 2	ASGR2		2	-4,34E-08	1	0
Q14103;Q14103-3;D6RF44;F5H6R6;H0YA96;D6RAF8;H0Y8G5;B4DTC3;Q14103-4;Q14103-2	Q14103;Q14103-3;D6RF44;F5H6R6;H0YA96;D6RAF8;H0Y8G5;B4DTC3;Q14103-4;Q14103-2	Heterogeneous nuclear ribonucleoprotein D0	HNRNPD		1	0,000362546	0,999074946	-0,001837651
P62263;H0YB22;E5RH77	P62263;H0YB22;E5RH77	40S ribosomal protein S14	RPS14		4	0,011522826	0,998883117	-0,008179824
B4DYH1;Q92598;E7EUG1;Q92598-2;Q92598-3	B4DYH1;Q92598;E7EUG1;Q92598-2;Q92598-3	Heat shock protein 105 kDa	HSPH1		2	0,007210751	0,997157667	-0,009518544
Q9H0D6;B4DZC3;Q9H0D6-2	Q9H0D6;B4DZC3;Q9H0D6-2	5-3 exoribonuclease 2	XRN2		1	0,014746225	0,995973913	-0,015027285
Q07065	Q07065	Cytoskeleton-associated protein 4	CKAP4		2	0,022807073	0,990296943	-0,019991239
P62906	P62906	60S ribosomal protein L10a	RPL10A		6	0,015498973	0,989559913	-0,02071619
Q86X95;Q86X95-2	Q86X95;Q86X95-2	Corepressor interacting with RBPJ 1	CIR1		2	0,011823567	0,988787746	-0,033339103
P60228;E5RIT4;H0YBR5;E5RHS5;E5RGA2	P60228;E5RIT4;H0YBR5;E5RHS5;E5RGA2	Eukaryotic translation initiation factor 3 subunit E	EIF3E		2	0,019707031	0,986534216	-0,035711606
Q8TA86;C9J6V2	Q8TA86	Retinitis pigmentosa 9 protein	RP9		6	0,036852727	0,963837104	-0,07783699
Q8NDV7;Q8NDV7-2;Q8NDV7-6;Q8NDV7-5;C9JA83	Q8NDV7;Q8NDV7-2;Q8NDV7-6;Q8NDV7-5;C9JA83	Trinucleotide repeat-containing gene 6A protein	TNRC6A		2	0,030608724	0,964261851	-0,083133698
Q9BU76;Q9BU76-4	Q9BU76;Q9BU76-4	Multiple myeloma tumor-associated protein 2	MMTAG2		1	0,118216099	0,941761124	-0,093201637
P29966	P29966	Myristoylated alanine-rich C-kinase substrate	MARCKS		5	0,089724674	0,943330233	-0,09609429
P14866;A6NIT8	P14866;A6NIT8	Heterogeneous nuclear ribonucleoprotein L	HNRNPL		5	0,056452708	0,947972286	-0,103621324
Q7L4I2;Q7L4I2-2;F5GXM2;H0YGP9;E1B6W4	Q7L4I2;Q7L4I2-2;F5GXM2	Arginine/serine-rich coiled-coil protein 2	RSRC2		3	0,04103391	0,948155251	-0,107248465
Q32MZ4;Q32MZ4-3;Q32MZ4-2	Q32MZ4;Q32MZ4-3;Q32MZ4-2	Leucine-rich repeat flightless-interacting protein 1	LRRFIP1		5	0,03237685	0,958906606	-0,109839837
P35637;B4DR70;E7EUX0;P35637-2;A6NMX6;Q92804;F5GWQ7;Q92804-2	P35637;B4DR70;E7EUX0;P35637-2;A6NMX6	RNA-binding protein FUS	FUS		6	0,067753047	0,941324009	-0,110818227
P42167;G5E972;P42167-2;H0YJH7	P42167;G5E972;P42167-2	Lamina-associated polypeptide 2, isoforms beta/gamma;Thymopoietin;Thymopentin	TMPO		11	0,051575394	0,948685185	-0,11185201
Q07020;F8VUA6;G3V203;H0YHA7;F8VWC5;F8VYV2;E7EW92	Q07020;F8VUA6;G3V203;H0YHA7;F8VWC5;F8VYV2;E7EW92	60S ribosomal protein L18	RPL18		3	0,091289792	0,929518868	-0,117965062
Q96HS1;Q96HS1-2	Q96HS1;Q96HS1-2	Serine/threonine-protein phosphatase PGAM5, mitochondrial	PGAM5		1	0,036396534	0,947935927	-0,129637639
P62805	P62805	Histone H4	HIST1H4A		9	0,065648458	0,939577465	-0,130579472
P05388;F8VU65;F8VWS0;Q3B7A4;F8VPE8;G3V210;F8VW21;Q8NHW5;F8VS58;F8VQY6;F8VRK7;F8VZS0;F8VWV4;F8W1K8	P05388;F8VU65;F8VWS0;Q3B7A4;F8VPE8;G3V210;F8VW21;Q8NHW5;F8VS58;F8VQY6;F8VRK7;F8VZS0;F8VWV4	60S acidic ribosomal protein P0;60S acidic ribosomal protein P0-like	RPLP0;RPLP0P6		9	0,275463318	0,894	-0,156896909
P15144;B4DP96	P15144;B4DP96	Aminopeptidase N	ANPEP		1	0,125392596	0,887211538	-0,161491235
Q9BLZ2	Q9BLZ2				6	0,037458193	0,945299304	-0,166909933
P10599;B1ALW1	P10599;B1ALW1	Thioredoxin	TXN		3	0,101416969	0,888258993	-0,175179482
P08865;C9J9K3;A6NE09	P08865;C9J9K3;A6NE09	40S ribosomal protein SA	RPSA;RPSAP58		1	0,04096868	0,94128972	-0,17644612
Q8TB52	Q8TB52	F-box only protein 30	FBXO30		4	0,152452578	0,888168734	-0,17871968
P51659;E7ER27;E7EWE5;E9PB82;F5HE57;E7EPL9;G5E9S2;E7ET17	P51659;E7ER27;E7EWE5;E9PB82;F5HE57;E7EPL9;G5E9S2;E7ET17	Peroxisomal multifunctional enzyme type 2;(3R)-hydroxyacyl-CoA dehydrogenase;Enoyl-CoA hydratase 2	HSD17B4		2	0,104626178	0,887708029	-0,187803507
P22061;P22061-2;B7Z972;F8WDT3;F8WAV5;F8WAX2	P22061;P22061-2;B7Z972	Protein-L-isoaspartate(D-aspartate) O-methyltransferase;Protein-L-isoaspartate O-methyltransferase	PCMT1		8	0,131779367	0,892681704	-0,19224596
P11940;E7EQV3;E7ERJ7;P11940-2;Q9H361;H0YAP2;H0YBQ4;H0YAR2;Q13310;Q13310-2;H0YC10;E5RGH3;H0YEQ8;E5RH24;E5RJB9;B1ANR1;H0YAS7;H0YB86;H0YAS6;H0YBN4;H0YB75;B1ANR0;Q4VC03	P11940;E7EQV3;E7ERJ7;P11940-2;Q9H361;H0YAP2;H0YBQ4;H0YAR2	Polyadenylate-binding protein 1;Polyadenylate-binding protein;Polyadenylate-binding protein 3	PABPC1;PABPC3		4	0,106380023	0,886614634	-0,196013769
O95487;O95487-2;B7ZKM8	O95487;O95487-2;B7ZKM8	Protein transport protein Sec24B	SEC24B		1	0,259490298	0,853973753	-0,218205293
P02545;P02545-2;Q6UYC3;P02545-3;Q5TCJ4;Q3BDU5;Q5TCI8;D6RAQ3;E7EUI9;H0YAB0;Q03252;E9PBF6	P02545;P02545-2;Q6UYC3;P02545-3;Q5TCJ4;Q3BDU5;Q5TCI8;D6RAQ3;E7EUI9	Prelamin-A/C;Lamin-A/C	LMNA		12	0,258166628	0,855694737	-0,226836205
P67809;H0Y449;E7ETA0;C9J5V9;P16989;Q9Y2T7;P16989-2;P16989-3	P67809;H0Y449;E7ETA0;C9J5V9	Nuclease-sensitive element-binding protein 1	YBX1		10	0,213188464	0,854104712	-0,233895461
Q96II8;E9PD99;Q96II8-3;B4E0T7;Q96II8-2;F8WFB5;F5H3V2	Q96II8;E9PD99;Q96II8-3;B4E0T7;Q96II8-2;F8WFB5;F5H3V2	Leucine-rich repeat and calponin homology domain-containing protein 3	LRCH3		2	0,060177365	0,892181818	-0,26789705
P62829;C9JD32;B9ZVP7	P62829;C9JD32;B9ZVP7	60S ribosomal protein L23	RPL23		3	0,377607185	0,79368595	-0,272983392
P08754;P63092;P04899;P63096;P09471;P38405;P19087;A8MTJ3;Q5JWF2-2;Q5JWF2;P11488;P63092-3;P63092-2;A2A2R6;Q5JWD1;H0Y7E8;Q5JWE9;H0Y7F4;F5H4U5;P09471-2;A6NI00;Q86XU3	P08754;P63092;P04899;P63096;P09471;P38405;P19087;A8MTJ3;Q5JWF2-2;Q5JWF2;P11488;P63092-3;P63092-2;A2A2R6;Q5JWD1;H0Y7E8;Q5JWE9;H0Y7F4;F5H4U5;P09471-2;A6NI00;Q86XU3	Guanine nucleotide-binding protein G(k) subunit alpha;Guanine nucleotide-binding protein G(s) subunit alpha isoforms short;Guanine nucleotide-binding protein G(i) subunit alpha-2;Guanine nucleotide-binding protein G(i) subunit alpha-1;Guanine nucleotide-binding protein G(o) subunit alpha;Guanine nucleotide-binding protein G(olf) subunit alpha;Guanine nucleotide-binding protein G(t) subunit alpha-2;Guanine nucleotide-binding protein G(t) subunit alpha-3;Guanine nucleotide-binding protein G(s) subunit alpha isoforms XLas;Guanine nucleotide-binding protein G(t) subunit alpha-1	GNAI3;GNAS;GNAI2;GNAI1;GNAO1;GNAL;GNAT2;GNAT3;GNAT1		2	0,264492828	0,79873913	-0,286983967
P11717	P11717	Cation-independent mannose-6-phosphate receptor	IGF2R		2	0,250087294	0,796317808	-0,304974874
Q07666;E7ET98;Q07666-2;Q07666-3	Q07666;E7ET98;Q07666-2;Q07666-3	KH domain-containing, RNA-binding, signal transduction-associated protein 1	KHDRBS1		2	0,653855584	0,725154286	-0,308716297
P05783;F8VZY9;Q92764;CON__Q92764;C4AM86;CON__Q497I4;CON__H-INV:HIT000015463	P05783;F8VZY9	Keratin, type I cytoskeletal 18	KRT18		16	0,750332758	0,711556851	-0,320947011

P62899;P62899-2;C9JU56;B7Z4E3;B7Z4C8;Q6IRZ0;B8ZZK4	P62899;P62899-2;C9JU56;B7Z4E3;B7Z4C8;Q6IRZ0;B8ZZK4	60S ribosomal protein L31	RPL31		3	0,240757207	0,787745856	-0,327972889
P23246;P23246-2;H0Y9K7;H0Y9U2	P23246;P23246-2	Splicing factor, proline- and glutamine-rich	SFPQ		10	0,189025632	0,798897297	-0,334372997
Q00610;Q00610-2;P53675;P53675-2;F5H5N6	Q00610;Q00610-2	Clathrin heavy chain 1	CLTC		63	0,700175833	0,71480597	-0,338951906
P46783;F6U211	P46783;F6U211	40S ribosomal protein S10	RPS10		1	0,066772541	0,887217822	-0,342488368
P84098	P84098	60S ribosomal protein L19	RPL19		5	0,467256317	0,72504298	-0,349965096
P29401;B3KSI4;B4E022;E7EPA7;E9PFF2	P29401;B3KSI4;B4E022;E7EPA7;E9PFF2	Transketolase	TKT		2	0,106819776	0,85553125	-0,35063537
P09496-2;P09496;B4DIN1;P09496-3;F8WF69;F5H6N3;C9J8P9;C9JND5	P09496-2;P09496;B4DIN1;P09496-3;F8WF69	Clathrin light chain A	CLTA		8	0,511386781	0,711847059	-0,352508545
Q9Y4I1;F8WE88;G3V394;F8W6H6;Q9Y4I1-2;Q9Y4I1-3;E7ERV5;F5H363	Q9Y4I1;F8WE88;G3V394;F8W6H6;Q9Y4I1-2;Q9Y4I1-3;E7ERV5;F5H363	Unconventional myosin-Va	MYO5A		9	0,094016936	0,86485347	-0,35537521
P07910;P07910-2;G3V4C1;G3V2Q1;P07910-4;G3V576;G3V4W0;B4DY08;B2R603;P07910-3;O60812;G3V2D6;Q569J8;G3V5X6;G3V3K6;G3V251;B4DSU6;G3V555;G3V5V7;G3V575;G8JL89;G3V2H6;G3V4M8	P07910;P07910-2;G3V4C1;G3V2Q1;P07910-4;G3V576;G3V4W0;B4DY08;B2R603;P07910-3;O60812;G3V2D6	Heterogeneous nuclear ribonucleoproteins C1/C2;Heterogeneous nuclear ribonucleoprotein C-like 1	HNRNPC;HNRNPCL1		5	0,0910796	0,868244898	-0,35879151
E7ENH9;P53396;B4E3P0;P53396-2	E7ENH9;P53396;B4E3P0;P53396-2	ATP-citrate synthase	ACLY		4	0,087991923	0,8702711	-0,361609936
P42766;H0Y3A0;F2Z388	P42766;H0Y3A0;F2Z388	60S ribosomal protein L35	RPL35		2	0,392443554	0,716149856	-0,363968213
P07900;P07900-2;Q86U12;G3V2J8;Q58FF6;Q14568;Q58FG0	P07900;P07900-2;Q86U12	Heat shock protein HSP 90-alpha	HSP90AA1		15	0,261314783	0,749114206	-0,368668238
P84103;B4E241	P84103;B4E241	Serine/arginine-rich splicing factor 3	SRSF3		2	0,294364977	0,740347339	-0,370720545
P84090;G3V279	P84090;G3V279	Enhancer of rudimentary homolog	ERH		3	0,341360063	0,711304348	-0,39884758
Q9BPW8;C9JDV8;F8WCR5	Q9BPW8	Protein NipSnap homolog 1	NIPSNAP1		8	0,901530613	0,633482428	-0,404803276
P62917;E7EW01;E9PKZ0;E9PIZ3;E9PKU4;E9PP36;G3V1A1	P62917;E7EW01;E9PKZ0;E9PIZ3;E9PKU4;E9PP36;G3V1A1	60S ribosomal protein L8	RPL8		4	0,576191656	0,66106422	-0,408095837
P23396;E9PL09;E9PPU1;H0YEU2;E9PQ96;E9PSF4;E9PJH4;F2Z2S8;H0YF32;E9PK82;H0YCJ7;E9PL45;H0YES8;E9PQX2	P23396;E9PL09;E9PPU1;H0YEU2	40S ribosomal protein S3	RPS3		7	0,953413443	0,626	-0,411550045
O00571;B4E3E8;O15523;B4DLU5;B4DXX7;B4E3C4;B4E132;C9J8G5;C9J081;B4DLA0	O00571;B4E3E8;O15523;B4DLU5;B4DXX7	ATP-dependent RNA helicase DDX3X;ATP-dependent RNA helicase DDX3Y	DDX3X;DDX3Y		9	0,524238167	0,658518519	-0,42253383
P15880;H0YEN5;E9PM36;E9PMM9;E9PPT0;E9PQD7	P15880;H0YEN5;E9PM36;E9PMM9;E9PPT0;E9PQD7	40S ribosomal protein S2	RPS2		4	0,333358191	0,712189349	-0,429676374
Q8TEH3;Q8TEH3-6;Q8TEH3-7;Q8TEH3-2;Q8TEH3-3;Q8TEH3-4;Q8TEH3-5	Q8TEH3;Q8TEH3-6;Q8TEH3-7;Q8TEH3-2;Q8TEH3-3	DENN domain-containing protein 1A	DENND1A		4	0,214127128	0,734422535	-0,444782416
P26641;B4DTG2;B4DUP0	P26641;B4DTG2	Elongation factor 1-gamma	EEF1G		4	0,14016577	0,797615176	-0,447116216
P0CG47;P62979;P62987;F5H6Q2;F5GYU3;F5H2Z3;F5H265;F5H7Y5;B4DV12;F5H388;F5H747;F5GXK7;Q96C32;F5H7K6;F5H041;P0CG48;F5GYX6;F5H4D8;F5GZ39	P0CG47;P62979;P62987;F5H6Q2;F5GYU3;F5H2Z3;F5H265;F5H7Y5;B4DV12;F5H388;F5H747;F5GXK7;Q96C32;F5H7K6;F5H041;P0CG48;F5GYX6;F5H4D8	Polyubiquitin-B;Ubiquitin;Ubiquitin-40S ribosomal protein S27a;Ubiquitin;40S ribosomal protein S27a;Ubiquitin-60S ribosomal protein L40;Ubiquitin;60S ribosomal protein L40;Polyubiquitin-C;Ubiquitin	UBB;RPS27A;UBA52;UBC		3	1,167388524	0,578680412	-0,455391248
P08621;P08621-4;P08621-2	P08621;P08621-4;P08621-2	U1 small nuclear ribonucleoprotein 70 kDa	SNRNP70		2	0,106701153	0,826832891	-0,455675364
P14868;Q68CR9	P14868;Q68CR9	Aspartate--tRNA ligase, cytoplasmic	DARS		1	0,470391956	0,651205047	-0,459597905
Q76I76;F5H527	Q76I76;F5H527	Protein phosphatase Slingshot homolog 2	SSH2		2	0,273953217	0,714131737	-0,461800575
Q15154;E7ETA6;Q15154-2;C9JGS5;E7EV56;H0YBA1;A6NNN6	Q15154;E7ETA6;Q15154-2;C9JGS5;E7EV56;H0YBA1	Pericentriolar material 1 protein	PCM1		6	0,236580195	0,711433526	-0,475932042
P68431;P84243;Q71DI3;Q16695;B4DEB1;Q6NXT2;Q5TEC6	P68431;P84243;Q71DI3;Q16695;B4DEB1;Q6NXT2;Q5TEC6	Histone H3.1;Histone H3.3;Histone H3.2;Histone H3.1t;Histone H3;Histone H3.3C	HIST1H3A;H3F3A;HIST2H3A;HIST3H3;H3F3C;HIST2H3PS2		4	0,44907481	0,649924528	-0,480625788
P61254;A6NE05	P61254;A6NE05	60S ribosomal protein L26	RPL26		6	0,508340459	0,624776699	-0,487047672
P35579;P35579-2;H0Y3P4;Q5BKV1;B1AH99;REV__E7EQT4;REV__Q9UKV3-5;REV__Q9UKV3	P35579;P35579-2	Myosin-9	MYH9		188	0,961226051	0,575507042	-0,495010217
Q07157;Q07157-2;H0Y3R8;G5E9E7;G3V1L9	Q07157;Q07157-2;H0Y3R8;G5E9E7;G3V1L9	Tight junction protein ZO-1	TJP1		2	0,19713936	0,732284091	-0,500555595
B7Z6F8;Q14677;Q14677-2;H0YD52	B7Z6F8;Q14677;Q14677-2	Clathrin interactor 1	CLINT1		10	0,388542413	0,646873418	-0,502120813
P62249;Q6IPX4	P62249;Q6IPX4	40S ribosomal protein S16	RPS16		5	0,555534003	0,59576	-0,518814882
P17844;Q92841;Q92841-3;C9JMU5;Q59F66;E7ETL9;Q92841-1;Q92841-2	P17844;Q92841;Q92841-3;C9JMU5;Q59F66;E7ETL9;Q92841-1;Q92841-2	Probable ATP-dependent RNA helicase DDX5;Probable ATP-dependent RNA helicase DDX17	DDX5;DDX17		4	0,772424455	0,579715302	-0,520539602
P61247;D6RG13;D6RAT0;F5H4F9;H0Y8L7;D6RB09;E9PFI5;D6RGE0;D6RED7;D6RAS7;D6R9B6;H0Y9Y4;D6RI02	P61247;D6RG13;D6RAT0;F5H4F9;H0Y8L7;D6RB09;E9PFI5;D6RGE0;D6RED7;D6RAS7;D6R9B6;H0Y9Y4	40S ribosomal protein S3a	RPS3A		4	0,419563043	0,62585209	-0,529924393
Q15125	Q15125	3-beta-hydroxysteroid-Delta(8),Delta(7)-isomerase	EBP		1	0,106779507	0,815882038	-0,531663497
P22626;P22626-2	P22626;P22626-2	Heterogeneous nuclear ribonucleoproteins A2/B1	HNRNPA2B1		9	0,209489662	0,711709302	-0,545643965
P52926;F5H2U8;F5H2A4;P52926-2;F5H6H0;Q1M185;Q1M188;Q1M186;Q1M187	P52926;F5H2U8;F5H2A4;P52926-2;F5H6H0;Q1M185;Q1M188;Q1M186;Q1M187	High mobility group protein HMGI-C	HMGA2		4	0,279750285	0,674682927	-0,546216329
Q02878;F8W181;F8VZ45;F8VR69;F8VZA3;F8VWR1	Q02878;F8W181	60S ribosomal protein L6	RPL6		8	0,841489524	0,548237037	-0,561595917
P51991;E7EWI9;E7ERJ4;P51991-2	P51991;E7EWI9;E7ERJ4;P51991-2	Heterogeneous nuclear ribonucleoprotein A3	HNRNPA3		6	0,594332549	0,577659574	-0,564009349
Q6ZN17;Q6ZN17-2	Q6ZN17;Q6ZN17-2	Protein lin-28 homolog B	LIN28B		1	0,580695083	0,56134767	-0,593411684
P62241;Q5JR95	P62241;Q5JR95	40S ribosomal protein S8	RPS8		4	0,772899981	0,537429658	-0,597660383
P47756-2;B1AK87;B1AK88;E7EQ40;P47756;F6USW4;B1AK85	P47756-2;B1AK87;B1AK88;E7EQ40;P47756	F-actin-capping protein subunit beta	CAPZB		3	0,338952173	0,625503268	-0,602247238
Q9P0K7;E7EMX7;E9PED3;Q9P0K7-3;Q9P0K7-2;D6RB27;D6R9G4;D6RBY4;D6REL2;D6R9G6;D6RF74;D6RE17;D6RB25;D6RBW6	Q9P0K7;E7EMX7;E9PED3;Q9P0K7-3;Q9P0K7-2	Ankycorbin	RAI14		19	0,355904387	0,595684211	-0,624955177
P46781;B5MCT8;C9JM19;F2Z3C0;A8MXK4	P46781;B5MCT8;C9JM19	40S ribosomal protein S9	RPS9		6	0,416135398	0,582894198	-0,626235008
P20042	P20042	Eukaryotic translation initiation factor 2 subunit 2	EIF2S2		1	0,113891473	0,792516484	-0,626676639
Q9UN86;Q9UN86-2;D6RFJ8	Q9UN86;Q9UN86-2;D6RFJ8	Ras GTPase-activating protein-binding protein 2	G3BP2		4	0,19060159	0,705891566	-0,64609313
Q9NZI8;C9JT33	Q9NZI8	Insulin-like growth factor 2 mRNA-binding protein 1	IGF2BP1		7	0,387957215	0,583520548	-0,647755623
P35268	P35268	60S ribosomal protein L22	RPL22		2	0,302346815	0,624065147	-0,65443929
P40939;B4DYP2	P40939;B4DYP2	Trifunctional enzyme subunit alpha, mitochondrial;Long-chain enoyl-CoA hydratase;Long chain 3-hydroxyacyl-CoA dehydrogenase	HADHA		2	0,540276695	0,548752768	-0,656244755
P36578;E7EWF1;B4DMJ2;B4DP82	P36578;E7EWF1;B4DMJ2	60S ribosomal protein L4	RPL4		9	0,775433753	0,490224	-0,667078495
P26373;F5H1S2	P26373;F5H1S2	60S ribosomal protein L13	RPL13		5	0,526694953	0,539257576	-0,689996401
P51654;H0Y3U6;G3V1R0;C9JLE3;REV__Q96HE7	P51654;H0Y3U6;G3V1R0;C9JLE3	Glypican-3;Secreted glypican-3	GPC3		3	0,389618241	0,579428571	-0,694927375
P15531;P15531-2;E7ERL0;C9K028;E5RHP0	P15531;P15531-2;E7ERL0	Nucleoside diphosphate kinase A	NME1		4	0,335488799	0,589698997	-0,697076003
O94832;F2Z2U7	O94832;F2Z2U7	Unconventional myosin-Id	MYO1D		1	0,360308962	0,578875	-0,700670958
Q92786	Q92786	Prospero homeobox protein 1	PROX1		2	0,239396202	0,648406349	-0,701240222
P62269;Q5GGW2	P62269	40S ribosomal protein S18	RPS18		9	0,788274595	0,471557377	-0,705426375
Q9UBI6	Q9UBI6	Guanine nucleotide-binding protein G(I)/G(S)/G(O) subunit gamma-12	GNG12		2	0,232968922	0,65185	-0,706818819
Q86TG7;Q86TG7-2	Q86TG7;Q86TG7-2	Retrotransposon-derived protein PEG10	PEG10		1	0,678744514	0,485414634	-0,720086734
P62701;P22090;Q8TD47;C9JEH7;A6NH36;C9JQ55	P62701;P22090;Q8TD47;C9JEH7	40S ribosomal protein S4, X isoform;40S ribosomal protein S4, Y isoform 1;40S ribosomal protein S4, Y isoform 2	RPS4X;RPS4Y1;RPS4Y2		10	1,156030968	0,403093333	-0,726625919
P53680;P53680-2	P53680;P53680-2	AP-2 complex subunit sigma	AP2S1		4	0,327725939	0,579128028	-0,751958211
Q9BRJ6;C9JQV0	Q9BRJ6;C9JQV0	Uncharacterized protein C7orf50	C7orf50		1	0,162978556	0,712678571	-0,757556518

Q99729-3;D6R9P3;D6RD18;D6RBZ0;Q99729-2;Q99729;Q99729-4	Q99729-3;D6R9P3;D6RD18;D6RBZ0;Q99729-2;Q99729;Q99729-4	Heterogeneous nuclear ribonucleoprotein A/B	HNRNPAB		3	0,408266696	0,546926471	-0,762806813
P61313;E7EQV9;E7ENU7;E7EX53;Q642I1	P61313;E7EQV9;E7ENU7	60S ribosomal protein L15;Ribosomal protein L15	RPL15		9	0,834218211	0,418337662	-0,783864657
Q9ULV0;Q9H6Y6;Q7Z7A5;Q9NQX4;H0YM93;F5H231;H0YMK3;E9PE01;Q6P1W8	Q9ULV0;Q9H6Y6;Q7Z7A5	Unconventional myosin-Vb	MYO5B		8	0,3683102	0,559898917	-0,784199635
P42166	P42166	Lamina-associated polypeptide 2, isoform alpha;Thymopoietin;Thymopentin	TMPO		11	1,043926566	0,384621005	-0,786219279
Q92828	Q92828	Coronin-2A	CORO2A		2	0,292857828	0,582779661	-0,796479464
P46779;H0YLP6;H0YMF4;H0YKD8;C9JB50;G5E9L2;B4DEP9;E9PB24	P46779;H0YLP6;H0YMF4;H0YKD8;C9JB50;G5E9L2;B4DEP9;E9PB24	60S ribosomal protein L28	RPL28		3	0,29234581	0,586979866	-0,802519639
P63261;P60709;F5H0N0;B4E335;B4DW52;F5GYT4;Q5T8M7;F5H1C1;G5E9R0;E7EVS6;C9JUM1;C9JZR7;E9PG30;C9JTX5;Q6S8J3;A5A3E0;P0CG38;P0CG39;Q9BYX7;F8WCH0	P63261;P60709;F5H0N0;B4E335;B4DW52;F5GYT4;Q5T8M7;F5H1C1;G5E9R0;E7EVS6	Actin, cytoplasmic 2;Actin, cytoplasmic 2, N-terminally processed;Actin, cytoplasmic 1;Actin, cytoplasmic 1, N-terminally processed	ACTG1;ACTB		27	1,884757765	0,326021739	-0,803007603
P60660;P60660-2;F8W1R7;G8JLA2;G3V1V0;B7Z6Z4;F8VPF3;F8VZV5;G3V1Y7;F8VZU9;F8W180;H0YI43;F8VXL3	P60660;P60660-2;F8W1R7;G8JLA2;G3V1V0;B7Z6Z4;F8VPF3;F8VZV5;G3V1Y7;F8VZU9;F8W180	Myosin light polypeptide 6	MYL6		12	1,162110695	0,373603865	-0,803075155
P62280	P62280	40S ribosomal protein S11	RPS11		4	1,017008958	0,384112676	-0,813319683
O00159;O00159-3;F5H6E2;F5H3W9;O00159-2;F5H730	O00159;O00159-3;F5H6E2;F5H3W9;O00159-2;F5H730	Unconventional myosin-Ic	MYO1C		15	0,447238465	0,519627907	-0,823454221
Q9Y2W1	Q9Y2W1	Thyroid hormone receptor-associated protein 3	THRAP3		2	0,387887147	0,545	-0,828540643
P12268;H0Y4R1;P20839;E7ETK5;C9K0R9;F5H247;C9J381;E7EQS0;P20839-2;B4DE09;P20839-3;C9JV30	P12268;H0Y4R1;P20839;E7ETK5;C9K0R9;F5H247;C9J381;E7EQS0;P20839-2;B4DE09;P20839-3;C9JV30	Inosine-5-monophosphate dehydrogenase 2;Inosine-5-monophosphate dehydrogenase 1;Inosine-5-monophosphate dehydrogenase	IMPDH2;IMPDH1		2	0,240640633	0,62261039	-0,829172929
Q86V81;E9PB61	Q86V81;E9PB61	THO complex subunit 4	ALYREF		3	0,465764698	0,50513834	-0,835365772
Q9UM54-6;E7EW20;Q9UM54;Q9UM54-5;Q9UM54-4;Q9UM54-2;Q9UM54-1	Q9UM54-6;E7EW20;Q9UM54;Q9UM54-5;Q9UM54-4;Q9UM54-2;Q9UM54-1	Unconventional myosin-VI	MYO6		42	1,137529648	0,357881188	-0,841987769
O14950	O14950	Myosin regulatory light chain 12B	MYL12B		15	1,150547714	0,352041026	-0,862584273
F5H8M4;F8WA45;P06396-2;P06396;F5H1A8;B7Z373;Q5T0H9;CON__Q3SX14;Q5T0H7;Q5T0H8;Q5T0I0;Q5T0I1	F5H8M4;F8WA45;P06396-2;P06396;F5H1A8;B7Z373;Q5T0H9	Gelsolin	GSN		12	0,933763884	0,370854369	-0,87642463
Q13148;B1AKP7;G3V162;Q13148-2	Q13148;B1AKP7;G3V162;Q13148-2	TAR DNA-binding protein 43	TARDBP		1	0,853551068	0,383018868	-0,879849593
O00567	O00567	Nucleolar protein 56	NOP56		2	0,594143315	0,431914894	-0,890515645
P35580-2;F8W6L6	P35580-2;F8W6L6	Myosin-10	MYH10		147	1,03104041	0,352525253	-0,893378417
P62753;A2A3R7	P62753;A2A3R7	40S ribosomal protein S6	RPS6		2	2,221326567	0,251168831	-0,903201739
O43795;E9PDF6;O43795-2;E7EPB4;E7EQD9;E9PHK2;C9JYW1;Q9UBC5;C9JUP5;Q8N1T3;G3V342;F5GWY8;F5H3C6	O43795;E9PDF6;O43795-2	Unconventional myosin-Ib	MYO1B		27	1,004970008	0,350428571	-0,907914162
O76021;B4DJ58	O76021;B4DJ58	Ribosomal L1 domain-containing protein 1	RSL1D1		1	0,70500807	0,388343891	-0,923863093
Q13045;E7EPM0;F5H407	Q13045;E7EPM0;F5H407	Protein flightless-1 homolog	FLII		14	0,761637674	0,382161137	-0,925226053
Q16698;E5RJD2;E5RJG7;E5RFV2;B7Z6B8	Q16698;E5RJD2;E5RJG7;E5RFV2;B7Z6B8	2,4-dienoyl-CoA reductase, mitochondrial	DECR1		1	0,88408226	0,356	-0,934940497
O15143;C9J4Z7;C9JEY1;C9JQM8;C9JFG9;C9J6C8;F8WCG3;C9JBJ7;C9K057;C9JTT6	O15143;C9J4Z7;C9JEY1;C9JQM8;C9JFG9;C9J6C8;F8WCG3	Actin-related protein 2/3 complex subunit 1B	ARPC1B		9	0,693767763	0,382506912	-0,950644016
Q13428;Q13428-6;Q13428-3;E7ETY2;E9PHK9;Q13428-7;Q13428-4;Q13428-5;Q13428-2;Q13428-8;H0YA99	Q13428;Q13428-6;Q13428-3;E7ETY2;E9PHK9;Q13428-7;Q13428-4;Q13428-5;Q13428-2;Q13428-8	Treacle protein	TCOF1		6	0,4998551	0,440235294	-0,970534643
P68133;P68032;P63267;P62736;Q5T8M8;A6NL76;E7EQV5;F8WB63;B8ZZJ2;C9JFL5;F6UVQ4;F6QUT6;B4DUI8	P68133;P68032;P63267;P62736;Q5T8M8;A6NL76;E7EQV5;F8WB63;B8ZZJ2;C9JFL5;F6UVQ4;F6QUT6;B4DUI8	Actin, alpha skeletal muscle;Actin, alpha cardiac muscle 1;Actin, gamma-enteric smooth muscle;Actin, aortic smooth muscle	ACTA1;ACTC1;ACTG2;ACTA2		20	0,558325303	0,411105263	-0,992306709
Q9P2E9;Q9P2E9-2;Q9P2E9-3;F8W7S5;A2A2S5;Q8N4C6;Q8N4C6-7;F8W6D3;H0YDJ4;H0YEL6;E9PN67;C9J066;Q8N4C6-6;Q5BKU3;Q8N4C6-4;Q8N4C6-9;Q8N4C6-2;Q8N4C6-5;Q8N4C6-10	Q9P2E9;Q9P2E9-2;Q9P2E9-3;F8W7S5	Ribosome-binding protein 1	RRBP1		26	0,783579776	0,35778	-0,992889722
P16401	P16401	Histone H1.5	HIST1H1B		4	0,44680055	0,458854772	-1,014334838
P0C0S8;Q9BTM1;Q99878;Q93077;Q7L7L0;P04908;Q96KK5;P20671;H0YFX9;P16104;Q6FI13;Q16777;C9JE22;Q9BTM1-2;Q96QV6	P0C0S8;Q9BTM1;Q99878;Q93077;Q7L7L0;P04908;Q96KK5;P20671;H0YFX9;P16104;Q6FI13;Q16777;C9JE22;Q9BTM1-2;Q96QV6	Histone H2A type 1;Histone H2A.J;Histone H2A type 1-J;Histone H2A type 1-C;Histone H2A type 3;Histone H2A type 1-B/E;Histone H2A type 1-H;Histone H2A type 1-D;Histone H2A;Histone H2AX;Histone H2A type 2-A;Histone H2A type 2-C;Histone H2A type 1-A	HIST1H2AG;H2AFJ;HIST1H2AJ;HIST1H2AC;HIST3H2A;HIST1H2AB;HIST1H2AH;HIST1H2AD;H2AFX;HIST2H2AA3;HIST2H2AC;HIST1H2AA		4	0,640888937	0,385495327	-1,015286446
P62136;E9PMD7;E9PDP1;Q07161;A6NNR3;P36873;F8VYE8;F8W0W8;P36873-2;B4DNE3;E7ETD8;F5H1L6;F5H037;F8W0V8;F8VR82	P62136;E9PMD7;E9PDP1;Q07161;A6NNR3	Serine/threonine-protein phosphatase PP1-alpha catalytic subunit;Serine/threonine-protein phosphatase	PPP1CA		5	0,904564025	0,328382979	-1,018614133
Q05682-3;E7EX44;Q05682-5;Q05682;Q05682-2;Q05682-4;E9PGZ1;F5H1Z9;A8K0X1;C9J813;E7EUR9;C9JEK3;C9JE79;F8WE61	Q05682-3;E7EX44;Q05682-5;Q05682;Q05682-2;Q05682-4;E9PGZ1;F5H1Z9;A8K0X1;C9J813	Caldesmon	CALD1		14	0,526962014	0,412422907	-1,020770868
P47914	P47914	60S ribosomal protein L29	RPL29		2	1,518533892	0,249904762	-1,028390725
O15027;O15027-2;O15027-4;O15027-3;O15027-5;A4QN19;H0Y5S1;C9JVR0;Q8N9G1;F6VLX6	O15027;O15027-2;O15027-4;O15027-3;O15027-5;A4QN19	Protein transport protein Sec16A	SEC16A		25	1,001849849	0,305681818	-1,039170106
P09327;Q96AC8;C9J2B5;O75366;O75366-2	P09327	Villin-1	VIL1		38	0,906877345	0,323142857	-1,046881517
B4DV78	B4DV78		VIL1		29	0,354373381	0,503920319	-1,057195981
Q13813;Q13813-3;F8W7K3;Q13813-2;A6NG51;B4DGT1;A6NN88	Q13813;Q13813-3;F8W7K3;Q13813-2;A6NG51	Spectrin alpha chain, non-erythrocytic 1	SPTAN1		57	0,748062951	0,34611399	-1,063443025
Q8WWI1-3;E9PMS6;Q8WWI1;Q8WWI1-2;E9PMT2;F8WD26;E9PLH4;Q8WWI1-4;E9PMP7;H0Y424;E9PJ10;H0YE95;E9PK58;E9PLU6;E9PRE3;E9PRJ0	Q8WWI1-3;E9PMS6;Q8WWI1;Q8WWI1-2;E9PMT2;F8WD26;E9PLH4;Q8WWI1-4;E9PMP7;H0Y424	LIM domain only protein 7	LMO7		54	0,981014194	0,305309942	-1,069109599
P52272;P52272-2;Q59ES8;F5H7Z1	P52272;P52272-2;Q59ES8	Heterogeneous nuclear ribonucleoprotein M	HNRNPM		19	0,933063834	0,307428571	-1,076541583
P32969;D6RAN4;B4E1M5;E7ESE0;H0Y9V9;H0Y9R4	P32969;D6RAN4;B4E1M5;E7ESE0;H0Y9V9	60S ribosomal protein L9	RPL9		4	1,068568951	0,282216867	-1,077063878
P83731;C9JXB8;C9JNW5	P83731;C9JXB8;C9JNW5	60S ribosomal protein L24	RPL24		2	0,657669408	0,361123153	-1,088589986
O95782;O95782-2	O95782;O95782-2	AP-2 complex subunit alpha-1	AP2A1		15	1,171818267	0,251425	-1,093432109
P61981;B4DE78;B4DHC4	P61981;B4DE78;B4DHC4	14-3-3 protein gamma;14-3-3 protein gamma, N-terminally processed	YWHAG		3	0,389375203	0,471802469	-1,0947601
Q7Z406-6;Q7Z406;Q7Z406-2;F2Z2U8;G8JLL9;Q7Z406-4;F2Z2U9;Q7Z406-5;F2Z2S7	Q7Z406-6;Q7Z406;Q7Z406-2;F2Z2U8;G8JLL9;Q7Z406-4;F2Z2U9;Q7Z406-5;F2Z2S7	Myosin-14	MYH14		78	1,216111399	0,248736842	-1,102802436
P43243;A8MXP9;D6REM6;D6R991;Q68E03;H0Y8T4;B3KM87;B7ZAV5	P43243;A8MXP9;D6REM6;D6R991	Matrin-3	MATR3		3	0,513986759	0,402785714	-1,104536772
P09493-3;D9YZV2;D9YZV8;H0YKX5;Q6ZN40;D9YZV3;Q9Y427;H0YKP3;H0YLS7;H0YKZ6	P09493-3;D9YZV2;D9YZV8;H0YKX5;Q6ZN40;D9YZV3;Q9Y427	Tropomyosin alpha-1 chain	TPM1		21	1,094797818	0,25590184	-1,112374306
P62266;D6RD47;D6RDJ2;D6RIX0;D6R9I7	P62266;D6RD47	40S ribosomal protein S23	RPS23		3	1,243351454	0,251369863	-1,114212831
Q02543;B4DM74	Q02543;B4DM74	60S ribosomal protein L18a	RPL18A		2	0,521827273	0,388109091	-1,120515664
Q01082;Q01082-3;Q01082-2;F8W6C1;O15020;O15020-2	Q01082;Q01082-3;Q01082-2	Spectrin beta chain, non-erythrocytic 1	SPTBN1		51	0,805196808	0,321171271	-1,124756495
P28289;Q5T7W3	P28289;Q5T7W3	Tropomodulin-1	TMOD1		2	1,082083167	0,252683544	-1,135717233

P62888;E5RI99;E5RJH3	P62888;E5RI99;E5RJH3	60S ribosomal protein L30	RPL30		2	0,635617236	0,351819095	-1,149315198
O75533;F8WC19;B4DGZ4;E9PCH3	O75533	Splicing factor 3B subunit 1	SF3B1		5	1,551647637	0,197448819	-1,152087609
Q14257;F8WCY5;A8MXP8;H0YL43	Q14257;F8WCY5;A8MXP8	Reticulocalbin-2	RCN2		9	0,791356535	0,316741573	-1,153793653
P63010;P63010-2;Q7Z451;F5GYG9	P63010;P63010-2;Q7Z451;F5GYG9	AP-2 complex subunit beta	AP2B1		20	1,005868442	0,254804878	-1,155614535
P63208;E5RJR5;E7ERH2;F8W8N3;P63208-2;E5RGM3	P63208;E5RJR5;E7ERH2;F8W8N3;P63208-2	S-phase kinase-associated protein 1	SKP1		4	0,899868206	0,285724551	-1,165046692
P09497-2;P09497;H0Y9Q6;D6RJD1	P09497-2;P09497	Clathrin light chain B	CLTB		8	1,346450696	0,219737226	-1,167593638
P14649;F8W1I5	P14649;F8W1I5	Myosin light chain 6B	MYL6B		7	1,152637653	0,242573427	-1,174995104
Q96FJ2;P63167	Q96FJ2;P63167	Dynein light chain 2, cytoplasmic;Dynein light chain 1, cytoplasmic	DYNLL2;DYNLL1		1	1,067581055	0,249621622	-1,19002835
P35244;B5MC59	P35244;B5MC59	Replication protein A 14 kDa subunit	RPA3		2	0,768179907	0,309175141	-1,196713448
O95831;O95831-3;O95831-2;E9PMA0;Q2QKE4;Q1L6K4;Q1L6K6	O95831;O95831-3;O95831-2;E9PMA0	Apoptosis-inducing factor 1, mitochondrial	AIFM1		9	0,419065909	0,424136752	-1,199960232
P62158;H0Y7A7;E7ETZ0;E7EMB3;Q96HY3;F8WBR5;G3V479;G3V361;G3V226;P27482;C9J7T9;P02585	P62158;H0Y7A7;E7ETZ0;E7EMB3;Q96HY3	Calmodulin	CALM1;CALM2		9	0,608298203	0,351959391	-1,211829027
P35611;P35611-2;H0Y9H2;E7EV99;E7ENY0;Q86XM2;A2A3N8;P35611-3;H0YFD8;H0YG19	P35611;P35611-2;H0Y9H2;E7EV99;E7ENY0;Q86XM2;A2A3N8;P35611-3	Alpha-adducin	ADD1		3	0,605170717	0,352907216	-1,213739157
P62081;B5MCP9	P62081;B5MCP9	40S ribosomal protein S7	RPS7		4	1,203007191	0,221122302	-1,214413643
P83111;P83111-2;H0YNN5	P83111;P83111-2	Serine beta-lactamase-like protein LACTB, mitochondrial	LACTB		5	1,125063973	0,234808511	-1,218050957
P98082;P98082-3;P98082-2;D6REB1;D6RIA5;D6RFF7;D6RGZ1	P98082;P98082-3;P98082-2	Disabled homolog 2	DAB2		14	0,516420277	0,380095694	-1,218931357
P62854;Q5JNZ5;F8VZW7	P62854;Q5JNZ5;F8VZW7	40S ribosomal protein S26;Putative 40S ribosomal protein S26-like 1	RPS26;RPS26P11		3	0,944295689	0,252025641	-1,231531779
Q9NYZ3	Q9NYZ3	G2 and S phase-expressed protein 1	GTSE1		2	1,368867046	0,200943089	-1,239433686
P06753-2;Q5VU59;P06753-3;Q5VU66;Q5VU72;Q5VU61;Q8NAG3;Q5VU63;D6R904;A6NL28-2;A6NL28;D6RFM2	P06753-2;Q5VU59;P06753-3;Q5VU66;Q5VU72;Q5VU61;Q8NAG3	Tropomyosin alpha-3 chain	TPM3;DKFZp686J1372		29	1,523189665	0,180103448	-1,243825595
O00422	O00422	Histone deacetylase complex subunit SAP18	SAP18		1	0,493861797	0,384018605	-1,244987806
P01859;P01857;P01861;P01860	P01859;P01857;P01861;P01860	Ig gamma-2 chain C region;Ig gamma-1 chain C region;Ig gamma-4 chain C region;Ig gamma-3 chain C region	IGHG2;IGHG1;IGHG4;IGHG3		2	0,297759866	0,521649805	-1,245557467
Q86XZ4;F8VS10	Q86XZ4;F8VS10	Spermatogenesis-associated serine-rich protein 2	SPATS2		1	0,4327311	0,406070796	-1,254816055
P19338;E7EX81;Q9BQ02;C9JYW2;C9JLB1;C9J1H7;C9JWL1	P19338;E7EX81;Q9BQ02	Nucleolin	NCL		18	0,955855959	0,249771812	-1,257276694
P67936;P67936-2	P67936;P67936-2	Tropomyosin alpha-4 chain	TPM4		20	1,360842468	0,196533333	-1,26132377
P38159;P38159-2;Q96E39	P38159;P38159-2;Q96E39	RNA-binding motif protein, X chromosome;RNA-binding motif protein, X chromosome, N-terminally processed;RNA binding motif protein, X-linked-like-1	RBMX;RBMXL1		5	0,503867412	0,378514286	-1,262648106
Q12965;H0YNQ8;O00160;H0YN00;H0YLJ4;H0Y2Q6;H0YLE5;H0YNB0	Q12965	Unconventional myosin-Ie	MYO1E		28	1,236932185	0,1994375	-1,272396882
P62750;A8MXA8;A8MUS3	P62750;A8MXA8;A8MUS3	60S ribosomal protein L23a	RPL23A		4	1,220025146	0,207162791	-1,274278323
Q9UPQ9;Q9UPQ9-1;A8MYY3;C9J5L7;H0Y720;Q9UPQ9-2	Q9UPQ9;Q9UPQ9-1;A8MYY3;C9J5L7;H0Y720;Q9UPQ9-2	Trinucleotide repeat-containing gene 6B protein	TNRC6B		7	0,892885519	0,252774194	-1,276017507
P08590;P05976;P05976-2	P08590;P05976;P05976-2	Myosin light chain 3;Myosin light chain 1/3, skeletal muscle isoform	MYL3;MYL1		1	1,073380582	0,219014925	-1,311360041
P62424;Q5T8U2;Q5T8U3	P62424;Q5T8U2;Q5T8U3	60S ribosomal protein L7a	RPL7A		7	1,371899805	0,177571429	-1,323257128
P40429;B4DNC8;Q6NVV1	P40429	60S ribosomal protein L13a	RPL13A		3	0,590442767	0,330189474	-1,331993421
P55209;H0YHC3;F8W020;F8W118;F8VXI6;F8VUX1;F8VY35;F8VV59;B7Z9C2;F8W0J6;F5H4R6;H0YIV4;B3KV44;B3KNT8;F8VRJ2;F5H1X5;H0YH88;F8W543;F8VVB5	P55209;H0YHC3;F8W020;F8W118;F8VXI6;F8VUX1;F8VY35;F8VV59;B7Z9C2;F8W0J6;F5H4R6;H0YIV4;B3KV44;B3KNT8;F8VRJ2;F5H1X5;H0YH88;F8W543	Nucleosome assembly protein 1-like 1	NAP1L1		3	0,998586107	0,219594203	-1,35528628
P06748;P06748-2;Q9BYG9;E5RGW4;E5RI98	P06748;P06748-2;Q9BYG9	Nucleophosmin	NPM1		8	0,815441315	0,250384106	-1,383205096
P61160;E9PF41;G3V1L8;F5H6T1	P61160;E9PF41;G3V1L8;F5H6T1	Actin-related protein 2	ACTR2		10	1,352793151	0,185714286	-1,388290246
P23528;E9PQB7;G3V1A4;E9PP50;E9PK25;E9PLJ3;E9PS23	P23528;E9PQB7;G3V1A4;E9PP50;E9PK25;E9PLJ3	Cofilin-1	CFL1		3	0,885483471	0,235492958	-1,394070625
Q9Y608-4;Q9Y608;C9JSU1	Q9Y608-4;Q9Y608	Leucine-rich repeat flightless-interacting protein 2	LRRFIP2		14	0,788550542	0,251267974	-1,406026999
Q86V48;Q86V48-2;Q86V48-3;E5RFK8	Q86V48;Q86V48-2;Q86V48-3	Leucine zipper protein 1	LUZP1		15	0,597255462	0,320826816	-1,41057841
O15511;O15511-2;B1ALC0	O15511;O15511-2;B1ALC0	Actin-related protein 2/3 complex subunit 5	ARPC5		4	1,116946173	0,188067227	-1,421689987
Q96SB8;Q96SB8-2	Q96SB8;Q96SB8-2	Structural maintenance of chromosomes protein 6	SMC6		2	0,250351957	0,544300752	-1,428668499
Q9NVI7-2;Q9NVI7;H0Y2W2;G3V1I6;Q5T9A4;E9PDY1;Q5T2N8;A8MSP6	Q9NVI7-2;Q9NVI7;H0Y2W2;G3V1I6	ATPase family AAA domain-containing protein 3A	ATAD3A		7	1,56565953	0,167487179	-1,43731308
P18621;B4E3C2	P18621;B4E3C2	60S ribosomal protein L17	RPL17		4	0,626681492	0,307105882	-1,438596725
O75531;E9PJJ8	O75531	Barrier-to-autointegration factor;Barrier-to-autointegration factor, N-terminally processed	BANF1		5	1,049681648	0,195834711	-1,44007953
P05387;H0YDD8;A6NIB2	P05387	60S acidic ribosomal protein P2	RPLP2		7	0,69528917	0,267830303	-1,44955651
Q13151	Q13151	Heterogeneous nuclear ribonucleoprotein A0	HNRNPA0		3	0,53770034	0,329026178	-1,44994688
Q9UPQ0;D6RD46;E9PHM7;Q9UPQ0-2;Q9UPQ0-4;Q9UPQ0-3;Q9UPQ0-6;E7EPK0;E9PDJ9;H0Y2S5;G5EA03;Q9UPQ0-9;Q9UPQ0-8;Q9UPQ0-5;H0Y8P3;F5GY62;D6RGH8;D6R8Y0;D6RJ93;Q6NVB9;Q9UPQ0-7	Q9UPQ0;D6RD46;E9PHM7;Q9UPQ0-2;Q9UPQ0-4;Q9UPQ0-3;Q9UPQ0-6;E7EPK0;E9PDJ9;H0Y2S5;G5EA03;Q9UPQ0-9;Q9UPQ0-8;Q9UPQ0-5;H0Y8P3	LIM and calponin homology domains-containing protein 1	LIMCH1		19	0,921674507	0,220606061	-1,451194445
P51114;P51114-3;E7ERF5;B4DXZ6;P51114-2;E7EU85;E9PFF5;P51116;Q06787;Q06787-7;C9IZ22;C9JZE0;C9JY20;F8W871;A8MQB8;G3V0J0;Q06787-8;Q06787-6;Q06787-2;Q06787-4;Q06787-5;G8JL90;Q06787-3	P51114;P51114-3;E7ERF5;B4DXZ6;P51114-2;E7EU85;E9PFF5	Fragile X mental retardation syndrome-related protein 1	FXR1		11	1,471281624	0,162988506	-1,455651442
Q9NYL9;H0YKU1;H0YNJ8;H0YNU8;Q9NZR1;G5EA42	Q9NYL9;H0YKU1;H0YNJ8	Tropomodulin-3	TMOD3		16	1,142024003	0,181541284	-1,471847375
P34741;E7ESK6;E9PBI9;F5H0S6;E5RHU3	P34741;E7ESK6;E9PBI9;F5H0S6;E5RHU3	Syndecan-2;Syndecan	SDC2		2	0,359884368	0,4188	-1,497411331
P62851	P62851	40S ribosomal protein S25	RPS25		3	1,161585023	0,183838384	-1,502191861
P16403;P16402;P22492	P16403;P16402	Histone H1.2;Histone H1.3	HIST1H1C;HIST1H1D		6	1,037165734	0,18235514	-1,555637995
P39023;F2Z3A5;G5E9G0;B5MCW2;F8WCR1	P39023;F2Z3A5;G5E9G0;B5MCW2	60S ribosomal protein L3	RPL3		9	1,312346698	0,166271605	-1,562366009
Q16643;A8MV58;Q16643-2;D6R9W4;F8W9Z3;D6R9Q9;D6RFI1;D6RCR4	Q16643;A8MV58;Q16643-2;D6R9W4	Drebrin	DBN1		18	0,729272328	0,244331034	-1,569107056
P61158;B4DXW1;F5H3P5;B4DTI0;Q9P1U1;Q9P1U1-2;Q9P1U1-3	P61158;B4DXW1;F5H3P5;B4DTI0	Actin-related protein 3	ACTR3		10	1,033846047	0,182777778	-1,571802934
Q96IZ0;F8W1M8	Q96IZ0;F8W1M8	PRKC apoptosis WT1 regulator protein	PAWR		2	1,086126889	0,177875	-1,604165077
P62140;C9J9S3;C9JP48	P62140	Serine/threonine-protein phosphatase PP1-beta catalytic subunit	PPP1CB		5	0,753704278	0,227	-1,632126967
Q9ULV4;A7MAP0;B4E3S0;H0YHL7;F8W1H8;F8VUX3;F8VSA4;F8VRE9;F8VTT6;F8VVB7;F8VV53	Q9ULV4;A7MAP0;B4E3S0	Coronin-1C;Coronin	CORO1C		10	0,850001755	0,199015873	-1,634865125
O43707;D6PXK4;E7EV83;P12814;F5GXS2;P12814-2;Q1HE25;B7TY16;P35609;G3V2W4;H0YGN3;B2RCS5;Q08043;H0YJ11;H0YJW3;G3V2N5;B7Z4P8	O43707;D6PXK4;E7EV83	Alpha-actinin-4	ACTN4		10	1,586316494	0,133152542	-1,656826655
Q00839;Q00839-2;B3KX72;Q5RI18	Q00839;Q00839-2;B3KX72	Heterogeneous nuclear ribonucleoprotein U	HNRNPU		8	0,8007398	0,205569231	-1,675301234
O15145;B4DM63;C9JZD1;F8VR50	O15145;B4DM63	Actin-related protein 2/3 complex subunit 3	ARPC3		8	1,445739362	0,139746032	-1,694018523
P62306;F8W0W6	P62306;F8W0W6	Small nuclear ribonucleoprotein F	SNRPF		2	0,321900754	0,421716738	-1,695537011
Q13492;A8MX97;Q13492-3;Q13492-2;F8VPG7;E9PN05;H0YE97;H0YEY8;H0YCY1;H0YD48;H0YEF7;H0YEH1;E9PJT1;E9PK13	Q13492;A8MX97;Q13492-3;Q13492-2;F8VPG7;E9PN05;H0YE97;H0YEY8;H0YCY1;H0YD48;H0YEF7;H0YEH1	Phosphatidylinositol-binding clathrin assembly protein	PICALM		4	0,472446934	0,330899471	-1,701793671

Q13501-2;Q13501;E9PFW8;E7ERP8;E7EMC7;C9JRJ8	Q13501-2;Q13501;E9PFW8;E7ERP8;E7EMC7	Sequestosome-1	SQSTM1		3	0,410550216	0,369470588	-1,704598427
Q12792;Q12792-3;F8VRG3;F8VS81;Q12792-4	Q12792;Q12792-3;F8VRG3;F8VS81;Q12792-4	Twinfilin-1	TWF1		2	1,712146079	0,1244	-1,738433202
P18827;E9PHH3	P18827;E9PHH3	Syndecan-1	SDC1		2	0,72794327	0,221422222	-1,741386255
Q3V6T2;B7ZM78;Q3V6T2-3;A6NC98;A6NC98-2;A6NC98-4;Q3V6T2-4;Q3V6T2-2	Q3V6T2;B7ZM78;Q3V6T2-3;A6NC98;A6NC98-2;A6NC98-4;Q3V6T2-4;Q3V6T2-2	Girdin;Coiled-coil domain-containing protein 88B	CCDC88A;CCDC88B		2	0,375592042	0,383074074	-1,74181207
Q8WWM7;Q8WWM7-6;Q8WWM7-5;Q8WWM7-4;A8K1R6;Q8WWM7-2;Q8WWM7-3;C9JXV8;Q8WWM7-7	Q8WWM7;Q8WWM7-6;Q8WWM7-5;Q8WWM7-4;A8K1R6;Q8WWM7-2;Q8WWM7-3;C9JXV8;Q8WWM7-7	Ataxin-2-like protein	ATXN2L		3	1,061806397	0,162636364	-1,772500912
O94973;O94973-2;E7ESQ4;E9PJJ7;C9J1S3;O94973-3;E9PPZ3;E9PS94;E9PQP4;E9PPY8;H0YEG0;H0YDE9;E7EVC3;E9PR62;E9PBJ2	O94973;O94973-2;E7ESQ4;E9PJJ7;C9J1S3;O94973-3	AP-2 complex subunit alpha-2	AP2A2		12	1,632175051	0,124244898	-1,802592278
Q969Q0;P83881;H0Y5B4	Q969Q0;P83881;H0Y5B4	60S ribosomal protein L36a-like;60S ribosomal protein L36a	RPL36AL;RPL36A		2	0,283606164	0,458627615	-1,845775366
O15144;E7EWG5;C9JTV5;G5E9J0;G5E9S7	O15144;E7EWG5	Actin-related protein 2/3 complex subunit 2	ARPC2		12	0,88073671	0,18336	-1,851837476
Q6WCQ1;Q6WCQ1-3;Q6WCQ1-2;H0Y7E2;H0Y2S9;C9J9W5;A8MW37	Q6WCQ1;Q6WCQ1-3;Q6WCQ1-2	Myosin phosphatase Rho-interacting protein	MPRIP		19	1,288494011	0,135409836	-1,874014695
Q15185;B4DP11;B4DDC6;B4DP21;B4DHP2	Q15185;B4DP11;B4DDC6;B4DP21;B4DHP2	Prostaglandin E synthase 3	PTGES3		2	0,813408658	0,179890909	-1,899197976
O75083;B4DY05;A8MVA9;O75083-3	O75083;B4DY05;A8MVA9;O75083-3	WD repeat-containing protein 1	WDR1		2	0,717079219	0,19984	-1,931005955
Q96D71;Q96D71-3;H0YDT0;Q96D71-2;H0YCR2;E9PMG1;H0YE89;Q8NFH8;H0YE73;B4DQQ8;Q8NFH8-3;Q8NFH8-2;Q8NFH8-4	Q96D71;Q96D71-3;H0YDT0;Q96D71-2;H0YCR2;E9PMG1	RalBP1-associated Eps domain-containing protein 1	REPS1		7	0,764786649	0,178070175	-1,982634226
O14976;E9PGR2;H0Y8H5;Q6P490	O14976;E9PGR2;H0Y8H5;Q6P490	Cyclin-G-associated kinase	GAK		5	1,1051893	0,142953846	-2,021126429
Q69YQ0;F5H1H6;F8WAN1;H0Y3Q3;C9J8U1;C9JLY8	Q69YQ0;F5H1H6;F8WAN1;H0Y3Q3;C9J8U1	Cytospin-A	SPECC1L;SPECC1L-ADORA2A		23	0,944946124	0,167189873	-2,036915938
P62913;P62913-2;Q5VVC9;Q5VVC8	P62913;P62913-2;Q5VVC9;Q5VVC8	60S ribosomal protein L11	RPL11		4	2,306171952	0,068	-2,039161841
Q08211;F5GXA5;REV__H0YA90;REV__Q7Z6J0-3	Q08211;F5GXA5	ATP-dependent RNA helicase A	DHX9		4	0,578391842	0,243694444	-2,049485127
Q14247;Q96H99;Q8N707;H0YEV2;H0YCD9;B4E358	Q14247;Q96H99;Q8N707	Src substrate cortactin	CTTN		4	1,400988388	0,118086957	-2,051763535
Q96N67;Q96N67-3;Q96N67-2;Q96N67-4;Q96N67-5;Q96N67-6;H0Y7L2;Q96NI0;F8W9H6;Q8NF50;A2A369;F8W9H3;E9PH09;Q8NF50-3;Q8NF50-2	Q96N67;Q96N67-3;Q96N67-2;Q96N67-4;Q96N67-5;Q96N67-6;H0Y7L2	Dedicator of cytokinesis protein 7	DOCK7		13	1,558440895	0,091609756	-2,054767132
P61513;C9J4Z3;G5E9R3;E9PEL3	P61513;C9J4Z3;G5E9R3;E9PEL3	60S ribosomal protein L37a	RPL37A		3	0,456656627	0,308022989	-2,05644242
Q9NR30;Q9NR30-2	Q9NR30;Q9NR30-2	Nucleolar RNA helicase 2	DDX21		2	0,93594854	0,168155844	-2,059725046
Q86XJ1;H0YIT6;G3V1N3	Q86XJ1;H0YIT6;G3V1N3	GAS2-like protein 3	GAS2L3		4	1,045164688	0,149391304	-2,0695858
P46777;B3KTM6	P46777;B3KTM6	60S ribosomal protein L5	RPL5		2	0,790643819	0,181278351	-2,089658817
P11387;Q969P6;E5RJ33;H0YBR3;H0YC03;E5RGE7;E5RGR4;E5RFE3;E5RGR2;E5RJ95;E5RFS0;E5RIC7;E7ES89	P11387	DNA topoisomerase 1	TOP1		3	1,016300102	0,143705882	-2,123803139
Q9UQR1;F5H173	Q9UQR1	Zinc finger protein 148	ZNF148		3	1,220251348	0,121923077	-2,136681636
Q9NSY1;H0Y9P1;C9JC56;Q9NSY1-2;Q9NSY1-3	Q9NSY1;H0Y9P1;C9JC56;Q9NSY1-2;Q9NSY1-3	BMP-2-inducible protein kinase	BMP2K		3	1,243680028	0,122980392	-2,143558502
P59998;F8WCF6;C9JWM7;F6TTL5	P59998;F8WCF6;C9JWM7	Actin-related protein 2/3 complex subunit 4	ARPC4;ARPC4-TTLL3		7	0,976651962	0,151042254	-2,156411489
P52907	P52907	F-actin-capping protein subunit alpha-1	CAPZA1		4	0,605311061	0,219794118	-2,162485917
F5H7S3;B7Z596;P09493-5;H0YNC7;H0YL42;H0YK20;H0YN06	F5H7S3;B7Z596;P09493-5;H0YNC7		TPM1		22	1,277106254	0,108888889	-2,2060558
Q92747;E9PF58;B4DLQ7	Q92747;E9PF58;B4DLQ7	Actin-related protein 2/3 complex subunit 1A	ARPC1A		3	0,886584053	0,169157895	-2,216102441
P62314	P62314	Small nuclear ribonucleoprotein Sm D1	SNRPD1		1	0,315706282	0,39018018	-2,224414984
Q9H0E2;F2Z2Y8;H0Y3H1;E9PNS3;B3KR28;E7EN89;B3KXC6	Q9H0E2;F2Z2Y8;H0Y3H1;E9PNS3;B3KR28;E7EN89;B3KXC6	Toll-interacting protein	TOLLIP		2	0,639312765	0,198852459	-2,260072072
P53814;P53814-5;E7ETT8;P53814-6;B5MC56;C9K039;P53814-2;B5MBZ4;C9JP19;C9JQZ8;B5MCI0	P53814;P53814-5;E7ETT8;P53814-6;B5MC56;C9K039	Smoothelin	SMTN		10	1,944103861	0,068714286	-2,30422322
P50402;Q5HY57	P50402;Q5HY57	Emerin	EMD		1	1,046342515	0,12962963	-2,327718337
O14974;F8VWB4;O14974-4;O14974-3;F5H1B6;O14974-2;F8W8Q6;F8W7P1;F8W9E8;F8VZN8;H0YHI8;H0YIS4;H0YIS3;H0YIM2;H0YIL7;F8VW28;H0YHL8	O14974;F8VWB4;O14974-4;O14974-3;F5H1B6;O14974-2;F8W8Q6;F8W7P1;F8W9E8;F8VZN8	Protein phosphatase 1 regulatory subunit 12A	PPP1R12A		22	0,858518791	0,170378378	-2,344146729
Q9HCJ0;G3XAB8;Q9HCJ0-2	Q9HCJ0;G3XAB8;Q9HCJ0-2	Trinucleotide repeat-containing gene 6C protein	TNRC6C		1	1,608394254	0,066971429	-2,353788376
Q9BPX5	Q9BPX5	Actin-related protein 2/3 complex subunit 5-like protein	ARPC5L		7	1,625927559	0,067411765	-2,362445513
H0YDQ3	H0YDQ3		LMO7		18	0,766816417	0,162382022	-2,410673618
Q14254;E7EMK3;E7EMK1	Q14254;E7EMK3;E7EMK1	Flotillin-2	FLOT2	+	2	3,081445395	0,018352941	-2,584759394
Q01130;F5GYB8;Q8NAK9;Q9BRL6;H0YG49;Q9BRL6-2	Q01130;F5GYB8;Q8NAK9;Q9BRL6;H0YG49;Q9BRL6-2	Serine/arginine-rich splicing factor 2;Serine/arginine-rich splicing factor 8	SRSF2;SRSF8		2	0,699660502	0,171032967	-2,616909981
P09651;F8VXY0;F8VRQ1;F8W6I7;P09651-3;P09651-2;F8VSB5;F8W1S7;Q32P51;F8VZ49;F8VYN5;F8VTQ5;H0YH80;F8W646	P09651;F8VXY0;F8VRQ1;F8W6I7;P09651-3;P09651-2;F8VSB5;F8W1S7;Q32P51;F8VZ49;F8VYN5;F8VTQ5	Heterogeneous nuclear ribonucleoprotein A1;Heterogeneous nuclear ribonucleoprotein A1, N-terminally processed;Heterogeneous nuclear ribonucleoprotein A1-like 2	HNRNPA1;HNRNPA1L2		11	1,153041365	0,091348837	-2,642175674
Q04837;E7EUY5;C9K0U8	Q04837;E7EUY5;C9K0U8	Single-stranded DNA-binding protein, mitochondrial	SSBP1		6	0,59539781	0,185220339	-2,701362769
Q92614;Q92614-4;F8W6U1;Q92614-3;Q92614-2;F8W6Y3;Q92614-5;E9PQN8;B4DFN7;H0YEF8;E9PPV5;H0YEV9	Q92614;Q92614-4;F8W6U1;Q92614-3;Q92614-2;F8W6Y3;Q92614-5;E9PQN8	Unconventional myosin-XVIIIa	MYO18A		27	0,736067639	0,164243902	-2,7059865
Q9UJC5	Q9UJC5	SH3 domain-binding glutamic acid-rich-like protein 2	SH3BGRL2		1	0,665876818	0,178086957	-2,754388968
P26599;P26599-2;Q9BUQ0;A6NLN1;O95758;O95758-2;O95758-1;B1ALY5;O95758-5;O95758-4;B1ALY2;B1ALY6	P26599;P26599-2;Q9BUQ0;A6NLN1	Polypyrimidine tract-binding protein 1	PTBP1		8	0,60643125	0,17827027	-2,789180756
P27694	P27694	Replication protein A 70 kDa DNA-binding subunit;Replication protein A 70 kDa DNA-binding subunit, N-terminally processed	RPA1		6	1,322299881	0,067	-2,815467993
Q07955;Q07955-3;Q07955-2	Q07955;Q07955-3;Q07955-2	Serine/arginine-rich splicing factor 1	SRSF1		2	0,861535915	0,129931034	-2,822558562
O43164;C9JUD6;O43164-2	O43164;C9JUD6;O43164-2	E3 ubiquitin-protein ligase Praja-2	PJA2		3	1,527679014	0,062782609	-3,09835132
P62910;D3YTB1;F8W727	P62910;D3YTB1;F8W727	60S ribosomal protein L32	RPL32		2	0,55337598	0,182393162	-3,145627658
P19532;B5MDQ7;P19484;O75030;O75030-9;O14948;O75030-5;B1AKB4;B7Z757;B1AKB5;C9JBI8;O14948-3;O14948-4;O14948-2;D3K197;P19484-2;O75030-10;E9PFN0;O75030-4;O75030-3;O75030-8;O75030-7;O75030-6;O75030-2	P19532;B5MDQ7;P19484;O75030;O75030-9;O14948;O75030-5;B1AKB4;B7Z757;B1AKB5;C9JBI8;O14948-3;O14948-4;O14948-2;D3K197;P19484-2;O75030-10;E9PFN0;O75030-4;O75030-3;O75030-8;O75030-7;O75030-6;O75030-2	Transcription factor E3;Transcription factor EB;Microphthalmia-associated transcription factor;Transcription factor EC	TFE3;TFEB;MITF;TFEC		2	1,058860499	0,081684211	-3,166210016
P39019	P39019	40S ribosomal protein S19	RPS19		4	0,90217011	0,115574468	-3,233526707
Q9UBP9;E9PB86;B8ZZ72;H0Y6R1;B8ZZL1;Q9UBP9-2	Q9UBP9;E9PB86;B8ZZ72;H0Y6R1	PTB domain-containing engulfment adapter protein 1	GULP1		6	0,681739254	0,169150685	-3,332801342
P62847;E7ETK0;P62847-2;P62847-3;E7EPK6	P62847;E7ETK0;P62847-2;P62847-3;E7EPK6	40S ribosomal protein S24	RPS24		1	0,654046961	0,1657	-3,346473535
Q9UHB6;Q9UHB6-4;B4DN52;F8VQE1;Q9UHB6-2;F8VRN8;F8VS07;Q9UHB6-3;F8VTU2;F8VVQ7	Q9UHB6;Q9UHB6-4;B4DN52;F8VQE1;Q9UHB6-2;F8VRN8;F8VS07;Q9UHB6-3	LIM domain and actin-binding protein 1	LIMA1		20	1,192420997	0,069125	-3,356943925
P46782	P46782	40S ribosomal protein S5;40S ribosomal protein S5, N-terminally processed	RPS5		3	1,25300622	0,065733333	-3,396877845

Q5VU58	Q5VU58	Tropomyosin alpha-3 chain	TPM3		28	1,379362305	0,0632	-3,415946007
Q9Y281;G3V5P4;F8WDN3;G3V2U0	Q9Y281;G3V5P4	Cofilin-2	CFL2		6	0,641070977	0,164144578	-3,434606393
P05386	P05386	60S acidic ribosomal protein P1	RPLP1		2	0,602895239	0,169955556	-3,509923776
P22695	P22695	Cytochrome b-c1 complex subunit 2, mitochondrial	UQCRC2	+	2	1,923461809	0,023111111	-3,56835866
P08708;P0CW22;H0YN73;H0YN88;H0YK46	P08708;P0CW22;H0YN73;H0YN88;H0YK46	40S ribosomal protein S17;40S ribosomal protein S17-like	RPS17;RPS17L		1	0,746155265	0,132210526	-3,613298416
P62244	P62244	40S ribosomal protein S15a	RPS15A		3	0,709327285	0,1439375	-3,623578389
Q7Z4V5;Q7Z4V5-2;C9JEE1	Q7Z4V5;Q7Z4V5-2;C9JEE1	Hepatoma-derived growth factor-related protein 2	HDGFRP2		2	0,689985309	0,145253731	-3,745971282
Q9NUQ6;Q9NUQ6-2;B8ZZZ7;B4DT67;Q4ZFW0;F8WAV0;C9IZC3;C9J8M7;F2Z2S1;C9JKE4;F8VZ02;F8VT91;C9JGM8;F8W6C2	Q9NUQ6;Q9NUQ6-2;B8ZZZ7;B4DT67	SPATS2-like protein	SPATS2L		4	1,240616482	0,063	-3,909059207
P46778;G3V1B3	P46778;G3V1B3	60S ribosomal protein L21	RPL21		4	0,658198554	0,152685714	-3,928734938
P18077;C9K025;F8WBS5;F8WB72	P18077;C9K025;F8WBS5;F8WB72	60S ribosomal protein L35a	RPL35A		2	0,858690354	0,092571429	-4,04088974
Q9NX58	Q9NX58	Cell growth-regulating nucleolar protein	LYAR	+	2	2,007656576	0,014571429	-4,050779661
P49715	P49715	CCAAT/enhancer-binding protein alpha	CEBPA	+	2	2,287375939	0,017	-4,323873043
Q58FF8	Q58FF8	Putative heat shock protein HSP 90-beta 2	HSP90AB2P		6	0,875221084	0,081945946	-4,4893864
P61163;F5H3I4;B4DXP9	P61163;F5H3I4;B4DXP9	Alpha-centractin	ACTR1A	+	1	3,865579413	0	-5,404199282
P61353	P61353	60S ribosomal protein L27	RPL27		4	0,775112478	0,083179487	-5,645527045
Q9Y3U8	Q9Y3U8	60S ribosomal protein L36	RPL36	+	2	2,826366065	0	-6,176116943
